# Drivers of stunting reduction in Senegal: a country case study

**DOI:** 10.1093/ajcn/nqaa151

**Published:** 2020-08-10

**Authors:** Samanpreet Brar, Nadia Akseer, Mohamadou Sall, Kaitlin Conway, Ibrahima Diouf, Karl Everett, Muhammad Islam, Papa Ibrahima Sylmang Sène, Hana Tasic, Jannah Wigle, Zulfiqar Bhutta

**Affiliations:** Centre for Global Child Health, Hospital for Sick Children, Toronto, Canada; Centre for Global Child Health, Hospital for Sick Children, Toronto, Canada; Dalla Lana School of Public Health, University of Toronto, Toronto, Canada; Université Cheikh Anta Diop, Dakar, Senegal; Centre for Global Child Health, Hospital for Sick Children, Toronto, Canada; Agence Nationale de la Statistique et de la Démographie, Dakar, Senegal; Centre for Global Child Health, Hospital for Sick Children, Toronto, Canada; Centre for Global Child Health, Hospital for Sick Children, Toronto, Canada; Agence Nationale de la Statistique et de la Démographie, Dakar, Senegal; Centre for Global Child Health, Hospital for Sick Children, Toronto, Canada; Centre for Global Child Health, Hospital for Sick Children, Toronto, Canada; Dalla Lana School of Public Health, University of Toronto, Toronto, Canada; Centre for Global Child Health, Hospital for Sick Children, Toronto, Canada; Dalla Lana School of Public Health, University of Toronto, Toronto, Canada; Center of Excellence in Women and Child Health, the Aga Khan University, Karachi, Pakistan

**Keywords:** stunting, linear growth, children, nutrition, exemplar, Senegal, West Africa, mixed methods

## Abstract

**Background:**

Senegal has been an exemplar country in the West African region, reducing child stunting prevalence by 17.9% from 1992 to 2017.

**Objectives:**

In this study, we aimed to conduct a systematic in-depth assessment of factors at the national, community, household, and individual levels to determine the key enablers of Senegal's success in reducing stunting in children <5 y old between 1992/93 and 2017.

**Methods:**

A mixed methods approach was implemented, comprising quantitative data analysis, a systematic literature review, creation of a timeline of nutrition-related programs, and qualitative interviews with national and regional stakeholders and mothers in communities. Demographic and Health Surveys and Multiple Indicator Cluster Surveys were used to explore stunting inequalities and factors related to the change in height-for-age z-score (HAZ) using difference-in-difference linear regression and the Oaxaca-Blinder decomposition method.

**Results:**

Population-wide gains in average child HAZ and stunting prevalence have occurred from 1992/93 to 2017. Stunting prevalence reduction varied by geographical region and prevalence gaps were reduced slightly between wealth quintiles, maternal education groups, and urban compared with rural residence. Statistical determinants of change included improvements in maternal and newborn health (27.8%), economic improvement (19.5%), increases in parental education (14.9%), and better piped water access (8.1%). Key effective nutrition programs used a community-based approach, including the Community Nutrition Program and the Nutrition Enhancement Program. Stakeholders felt sustained political will and multisectoral collaboration along with improvements in poverty, women's education, hygiene practices, and accessibility to health services at the community level reduced the burden of stunting.

**Conclusions:**

Senegal's success in the stunting decline is largely attributed to the country's political stability, the government's prioritization of nutrition and execution of nutrition efforts using a multisectoral approach, improvements in the availability of health services and maternal education, access to piped water and sanitation facilities, and poverty reduction. Further efforts in the health, water and sanitation, and agriculture sectors will support continued success.

## Introduction

**FIGURE 1:**
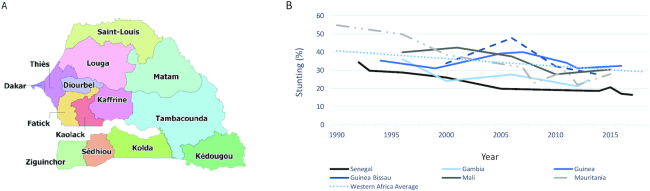
Map of Senegal (A). The prevalence of stunting in children <5 y old in Senegal and its neighboring countries in the West African Region, 1990–2018 (B). Source: (1).

Globally, the prevalence of childhood stunting has decreased by 17.3%, from 39.2% to 21.9%, between 1990 and 2018 (1). However, the West African region is lagging far behind the global average, achieving only an 11.5% stunting decline (1). Senegal, a Sub-Saharan African country bordered by the North Atlantic Ocean in the west (**Figure 1**A), has stood out as a low-income exemplar country (2). Between 1992 and 2017, Senegal achieved a 17.9% decline in the prevalence of stunting (1, 3). Compared with its neighboring countries, Senegal has maintained the lowest burden of stunting, with continued progress over the past 2 decades (Figure 1B). With a rapidly growing population, Senegal had an estimated 15.9 million inhabitants as of 2017, with over half (53.3%) residing in rural areas (4, 5). This population comprises 6 major ethnic groups: Wolof (37.1%), Pular (26.2%), Serer (17%), Mandinka (5.6%), Jola (4.5%), and Soninke (1.4%) (6). Although Senegal is largely a peaceful country, a low-level separatist conflict in the southern Casamance area occurred between 1982 and 2014, leading to 3000–5000 deaths and many internal displacements (7, 8). The environment of Senegal mainly consists of the sandy plains of the western Sahel and is prone to climate shocks, with a series of floods, erratic rainfall, droughts, and locusts that have affected hundreds of thousands of Senegalese since the 1990s (2, 9). Consequently, Senegal is highly food insecure and ranked 66 out of 119 countries on the 2018 Global Hunger Index (10). Despite these hardships, Senegal has achieved stable economic growth (**Supplementary Appendix 1**) following the devaluation of the CFA franc in 1994 and moderate poverty reduction with an increase in remittance income (11). Additionally, the nation has made modest improvements in several areas since the early 1990s, including overall literacy, gender equality, access to improved sanitation and clean water (Supplementary Appendix 1), reduction in total and adolescent fertility rates, and increased agricultural production and consumption.

Efforts have been made by previous cross-sectional and prospective national and subnational studies to uncover the determinants of stunting in Senegal. Only 1 published national study decomposed the roles of various factors that contributed to the 20.4% change in mean height-for-age z score (HAZ) from 1993 to 2011 using data from demographic and health surveys (DHS) (12). The authors of this national study found the following factors to explain 66% of the change in the mean HAZ: healthcare (33.5%), assets (16.1%), parental schooling (8.8%), mother's height (8.1%), and piped water (6.0%). Moreover, several studies have found urban residence, female sex, children aged <2 y, complementary feeding beginning at 6 mo, and children not having anemia or HIV to be associated with lower stunting among Senegalese children aged <5 y (13–26). Findings regarding the effects of household wealth; household size; child diet diversity; intestinal parasites; parental education; maternal height, age, and parity; prenatal consultations; health facility births; migration; and climate and economic shocks are mixed (12, 14, 17, 21, 23, 25–34). See **Panel 1** (12–17, 19, 21–28, 30, 31, 33–48) for a summary of relevant literature and **Supplementary Appendix 2** for the detailed literature review.

Panel 1 Systematic Literature Review of Stunting Determinants

Our literature review found that the basic causes of stunting in Senegal included climate shocks including rain, droughts and extreme cold (31, 42), economic shocks including periods of increasing food prices (2009–2011) (31), rural residence, household size, household wealth, and parental education. All studies were in agreement that children residing in rural areas were at a greater risk of stunting as compared to their urban counterparts (13–15, 43). However, these region-wide differences disappear when comparing children from similar wealth quintiles (14). Girls were found to have some gains with catch-up growth when they migrated for a short period of time to an urban area (Dakar) from a rural area (Niakhar) (17). A study examining migration patterns found that during periods of heat waves and droughts, individuals from areas where there is acute malnutrition are more likely to migrate compared to those from areas with chronic malnutrition (30). Two subnational studies found household size was not associated with chronic child malnutrition (21, 28) though in the regions of Fatick, Kaolack and Kolda, increasing household size was found to negatively affect HAZ (44). Four national studies and one subnational study found household wealth was associated with under-5 stunting (12, 14, 28, 43, 45) though three subnational studies found no association between wealth or housing quality with chronic child malnutrition (22, 29, 44). National studies found that maternal and parental education were important contributors of stunting reduction (12, 43). A study including three regions found maternal and parental education improved child HAZ (44) while one urban study and four studies from rural areas found maternal education had no effect (17, 21, 22, 28, 29). Through the use of proxy measures such as women's social status in the household including their age difference with respect to the head of the household and whether they are in a polygamous relationship, no association was found with child stunting (22, 44). No nutrition programs that were evaluated reduced the risk of stunting (21, 46, 47), however, the PRN reduced the risk of children being underweight (48).

Literature indicates that the underlying causes of stunting were lack of access to clean water, maternal and child healthcare, duration of breastfeeding, and age of introduction to complementary feeding. Two national studies did not find piped water, open defecation, or access to a toilet to be associated with stunting (12, 43). Conversely, improved piped water access was a contributor of stunting reduction between 1993 and 2011 (12). National studies have shown that 4+ antenatal care visits and giving birth in medical facilities were the largest contributors to stunting reduction between 1993 and 2014 (12, 43). Two subnational studies failed to find an association between prenatal consultation and place of birth with stunting – likely due to small sample sizes (21, 22). Nonetheless, a study conducted in three regions found that the presence of a health post but not of an NGO improved child HAZ (44). Two subnational studies found weaning children past the age of two increased the risk of stunting though another found no association between the breastfeeding duration and child HAZ (20, 49–51). Four studies in rural areas supported complementary feeding to commence no earlier than six months of age in order to support optimal child growth (23, 24, 52, 53). A subnational study found no association between complementary feeding before 6 months and introduction of water before 3 months with the risk of stunting in children between 6 and 23 months (21).

Immediate causes of stunting in Senegal include anemia (25, 54), intestinal parasitosis (33, 34), maternal height, parity, and child age and sex. Most subnational studies did not find evidence of the importance of maternal height or BMI (17, 22, 29), and maternal age (21, 22). A national study found maternal height was positively associated with child HAZ and contributed to the stunting decline between 1993 and 2011 while a subnational study concluded that being a teenage mother decreased child HAZ (12, 22, 44). Short birth interval has increased the risk of stunting in two studies while the number of children in a family was not associated with HAZ in four studies (12, 22, 29, 43, 44). Six studies found that boys are at a greater risk of being stunted (17–20, 43, 44) and four found no association between child sex and chronic malnutrition (21, 28, 29, 55). The risk of chronic malnutrition increases with age, up to 36 months, in all studies except two (17, 18, 20–22, 29, 44, 55). Not surprisingly, HAZ decreases more rapidly for boys leading to greater sex differences with increasing age.

Due to Senegal's progress across several sectors, a multifactorial stunting reduction success story is likely, warranting the exploration of these factors using a mixed-methods approach. However, to our knowledge, no previously published national studies have executed this approach to examine a wide array of stunting determinants by using both quantitative and qualitative methods or over a time period between 1992/93 and 2017. The vast majority of previous studies were based on subnational cross-sectional surveys and were not generalizable to the population of Senegal. Hence, in our study we aimed to do the following: *1*) quantitatively examine determinants of stunting reduction in Senegal and decompose long-term stunting change into relative contributions from key drivers; *2*) generate a systematic landscape of the major stunting-relevant policies and programs in Senegal, focusing on both nutrition-specific and nutrition-sensitive initiatives; and *3*) explore national- and community-level perspectives on Senegal's nutrition evolution (focused on progress in stunting) and the major contributing factors behind it.

## Methods

### Study design

**FIGURE 2:**
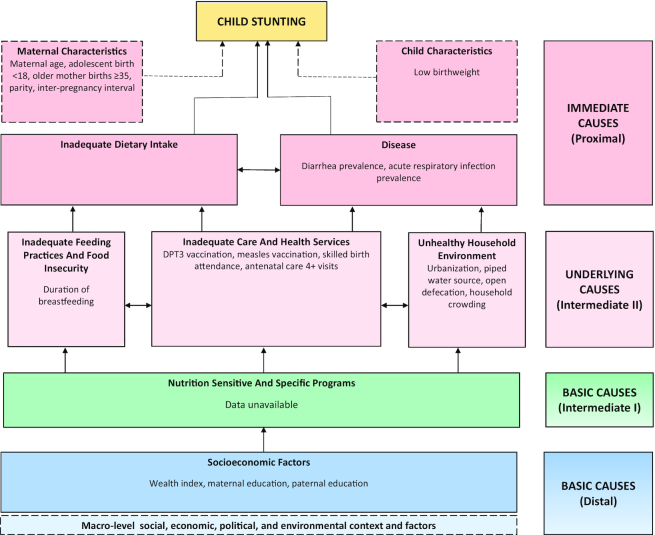
Conceptual framework showing distal, intermediate, and proximal determinants of stunting. Note: Maternal height, maternal BMI, maternal anemia, and vitamin A supplementation have been removed from analyses as these data were not available in all survey rounds, particularly in the DHS 1992/93 and 2017. Complementary feeding and inadequate dietary intake variables (including: minimum dietary diversity, grains, legumes, dairy, flesh foods, eggs, vitamin A–rich fruits and vegetables, other fruit and vegetables) were considered for inclusion, however, were only measured for the 6–23-mo old population; however, this subgroup was not analyzed. The total mean HAZ change for the 6–23-mo age group was too small for our model's results to be meaningful, and analysis resulted in a model with unstable results. Exclusive breastfeeding was also considered for inclusion, however it was only measured for the < 6-mo-old population, which was omitted from analysis due to small sample size. DHS, demographic and health survey; DPT3, diphtheria, pertussis, and tetanus; HAZ, height-for-age *z* score.

In this mixed methods study we applied several complementary approaches to inform study objectives, including a systematic literature review, a retrospective quantitative analysis, a program and policy analysis from 1990 to 2017, and qualitative data collection and analyses. An adapted conceptual framework (**Figure 2**) was designed based on the UNICEF nutrition framework and Lancet nutrition framework to guide all analyses (49, 50). Refer to the methods paper in this series [Akseer et al. (56)] for information on the development of a framework. Our analyses focused on Senegal's periods of stunting decline, which was gradual from the early 1990s to 2000, faster between 2000 and 2005, and then followed by a more marginal decline.

Ethics approval for the study, inclusive of primary data collection, was obtained through the Ministry of Health Ethics Board of the Cheikh Anta Diop University. Ethics approval for the broader stunting case study was also obtained through the Research Ethics Board at the Hospital for Sick Children (SickKids), in Toronto, Canada.

### Systematic literature review

We conducted a systematic scoping literature review between November 2017 and December 2018 of published peer-reviewed and grey literature to synthesize information on contextual factors, national and subnational interventions, policies, strategies, programs, and initiatives that may have contributed to reductions in child stunting in Senegal from 1990–2017. Using the search categories of *1*) stunting (e.g., stunting, linear growth, linear growth stunting, HAZ, height, height-for-age, LAZ, length, length-for-age, undernutrition, malnutrition, nutr*), *2*) child (e.g., child*, infan*), and *3*) Senegal (Senegal*), we searched >15 online databases and grey literature sources. After deduplication, 1728 records were found, of which 44 articles remained after full text review. Full methods and results of the systematic review are included in Supplementary Appendix 2.

### Quantitative methods

#### Data Sources

Senegal has had multiple sequential DHS performed over the past 3 decades, including serial DHS since 2012. We selected DHS that were considered good quality and that aligned with our study period (and stunting prevalence reduction trajectories) as the primary quantitative datasets used in this study. Available anthropometry data for children <5 y old by survey round are presented in **Table 1**.

**TABLE 1:** Sample size by survey year based on valid child anthropometric data^1^

	Year of survey			
Age group, *n*	DHS 1992/93	MICS 2000	DHS 2005	DHS 2017
<6 mo	383	838	269	737
6–23 mo	834	1892	636	2286
≥24 mo	623	1495	450	2053
<36 mo	1567	3445	1139	4013
<5 years	1840	4225	1355	5076

^1^Based on index (youngest) child data. DHS, demographic and health survey; MICS, multiple indicator cluster survey.

#### Outcomes and Covariables

We studied child HAZ and stunting prevalence (HAZ < −2SD) estimated using WHO child growth standards as the main study outcomes (51). Potential determinants or “covariables” were selected in line with our conceptual framework (Figure 2), as those that were distal, intermediate, and proximal factors to the child stunting outcome. Covariables were identified as individual- or household-level factors from DHS and Multiple Indicator Cluster Surveys (MICS). Although we searched for ecological variables (at the region level), we were unable to track meaningful and available indicators.

#### Statistical Analysis

We estimated child HAZ kernel density plots and HAZ compared with age plots (“Victora curves”) (52) using smoothed local polynomial regressions to examine population shifts in growth faltering and growth trajectories by period of life. We estimated piecewise linear splines to quantify the slopes and inflection points of growth trajectories (53). We used previously published standardized methods for understanding stunting prevalence by wealth quintile (Q1–Q5), maternal education, area of residence (urban or rural) and child gender (54, 55). Household asset data were used to estimate wealth scores (later organized into quintiles) using principal components analysis. To account for the cumulative distribution of the wealth index, we also calculated slope index of inequality (SII) and concentration index (CIX) to measure absolute and relative socioeconomic inequalities (54, 55), respectively. We examined relative declines in child stunting prevalence for each region in Senegal using compound annual growth rates (CAGRs).

To understand potential determinants of stunting reduction, we conducted two complementary sets of multivariable analyses to study relationships between child HAZ (outcome) and covariables. Both analyses used a series of step-wise linear regression models and hierarchical modelling of distal, intermediate, and proximal level variables as suggested by Victora 1997 (49). We harmonized variables in the DHS 1992/93 and DHS 2017 rounds and applied difference-in-difference methods with time*covariable interaction terms to examine if change in a proposed predictor of HAZ leads to a change in HAZ. Oaxaca-Blinder decomposition methods were applied to statistically decompose changes in mean child HAZ between 1992/93 and 2017 into potential determinants (covariables). Please see **Supplementary Appendix 3** and the Methods paper in this series [Akseer et al. (56)] for full methods detail. All analyses were conducted with Stata 14.0 and accounted for survey design and weighting.

### Policy and program review

Key nutrition-specific and -sensitive policies and programs that took place between 1990 and 2017 were assembled into a timeline using an iterative approach. The Senegal study principal investigator and research team members proposed a timeline based on a systematic desk review of literature. This timeline was reviewed by expert stakeholders and iterated on until agreement between the Senegal research team and country experts was reached.

### Qualitative methods

Qualitative interviews were conducted to gain perspectives of key national stakeholders, regional stakeholders (childcare and health workers), and mothers in the community. Participants were identified and selected using purposive sampling strategies (57), including snowball sampling in the regions of Louga, Diourbel, and Kaolack due to their substantial progress in reducing under-5 stunting (57, 58). First, 21 interviews were conducted with experts in Dakar who were officials from the Ministry of Health and other specialized agencies, international nongovernmental organizations (NGOs), and professors. Second, 20 interviews were conducted with resource persons, including nursery school teachers, health staff (i.e., doctors, nurses, midwives, and community health workers), NGO workers, imams, and village chiefs. In each of the 3 regions, 2 interviews with resource persons were conducted in rural areas and 2 in urban areas. Last, in each region, 2 focus groups were held with mothers who gave birth between 1992 and 1997 and 2 focus groups with younger women who gave birth between 2012 and 2017. National and regional interviews were conducted in French while focus groups were conducted in the main local language of Wolof, and were audiorecorded, transcribed for analysis, and translated into English. Data generated during focus group discussions and semistructured interviews were analyzed using our study framework. Thematic analysis was conducted to explore key themes that emerged based on stunting determinants, including socioeconomic status, migration, hygiene and sanitation, and nutrition and eating behaviors. Responses from national stakeholders, regional stakeholders, and mothers at the community level were analyzed separately. Full qualitative analysis methodology can be found in **Supplementary Appendix 4**.

## Results

### Descriptive analyses

#### HAZ Kernel Density Plots and Victora Curves

**FIGURE 3:**
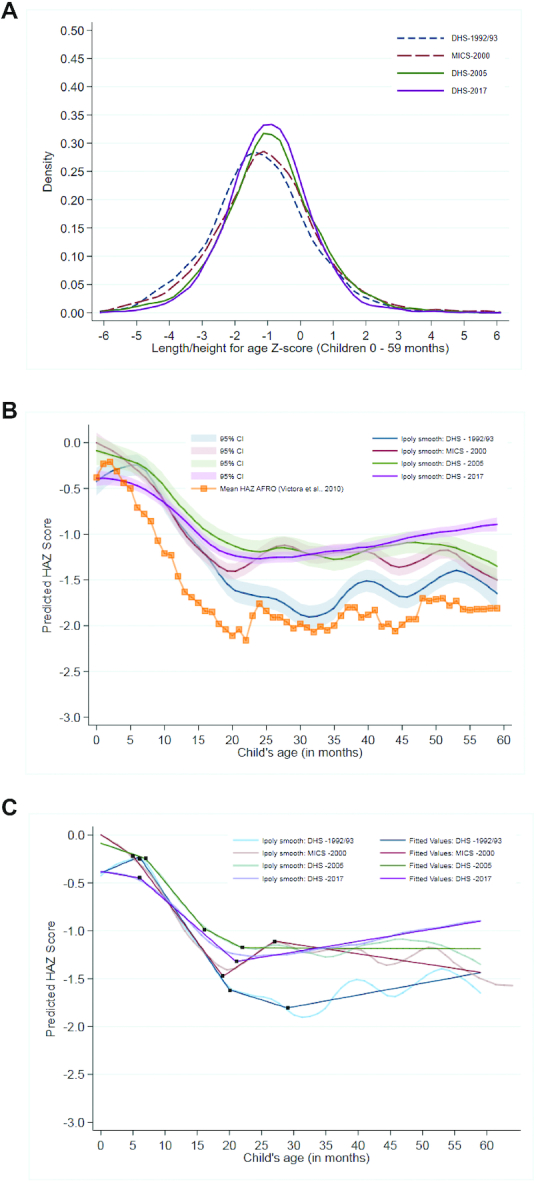
Kernel density plot for HAZ distribution in children < 5 years, 1992/93–2017 (A). Victora curve using data from the 1992/93, 2000, 2005, 2017 surveys among children < 5 years, including AFRO mean HAZ curve (B) (Source: Victora et al., 2010). Victora curve using data from the 1992/93, 2000, 2005, 2017 surveys among children <5 y old with linear splines (C). HAZ, height-for-age *z* score.

Population-wide gains in HAZ for children under 5 y old have been achieved between 1992/93 and 2017 as depicted in the HAZ kernel density plot in **Figure 3**A. From 1992/93 to 2000, the mean HAZ of children slightly improved, from −1.25 SDs to −1.04 SDs. From 2000 to 2005 (−0.92 SDs) to 2017 (−0.97 SDs), further improvements in mean HAZ were achieved alongside a decrease in inequalities. Examination of the growth faltering process between birth to 5 y of age is suggestive of improvements in both maternal and child nutrition between 1992/93 and 2017 as presented by the Victora growth curves (Figure 3B). Although predicted HAZ at birth, indicative of maternal nutrition, initially increased between 1992/93 to 2000 and 2000 to 2005, in 2017 it reverted to its 1992/93 starting point. However, the postnatal growth faltering process improved, with a less steep decline in HAZ between 6 and 24 mo of age between 1992/93 and 2017, indicating improvements in child nutrition. Piecewise linear splines overlaid on the Victora growth curves revealed that the growth faltering period began to shorten and the rate of decline in HAZ began to slow down over time (Figure 3C, **Supplementary Appendix 5**). In 1992/93, the growth faltering period lasted between 6 and 20 mo, with HAZ decreasing at a rate of 0.10 SD per mo (95% CI: −0.108, −0.091) and continued, although at a slower rate of 0.02 SD per mo (95% CI: −0.031, −0.010) between 20 and 29 mo (Supplementary Appendix 5). By 2017, the rate of growth faltering was reduced to 0.06 SD per mo (95% CI: −0.060, −0.057) and occurred between 6 and 21 mo, with HAZ improving after 21 mo of age (Supplementary Appendix 5).

#### Equity Analysis

**FIGURE 4:**
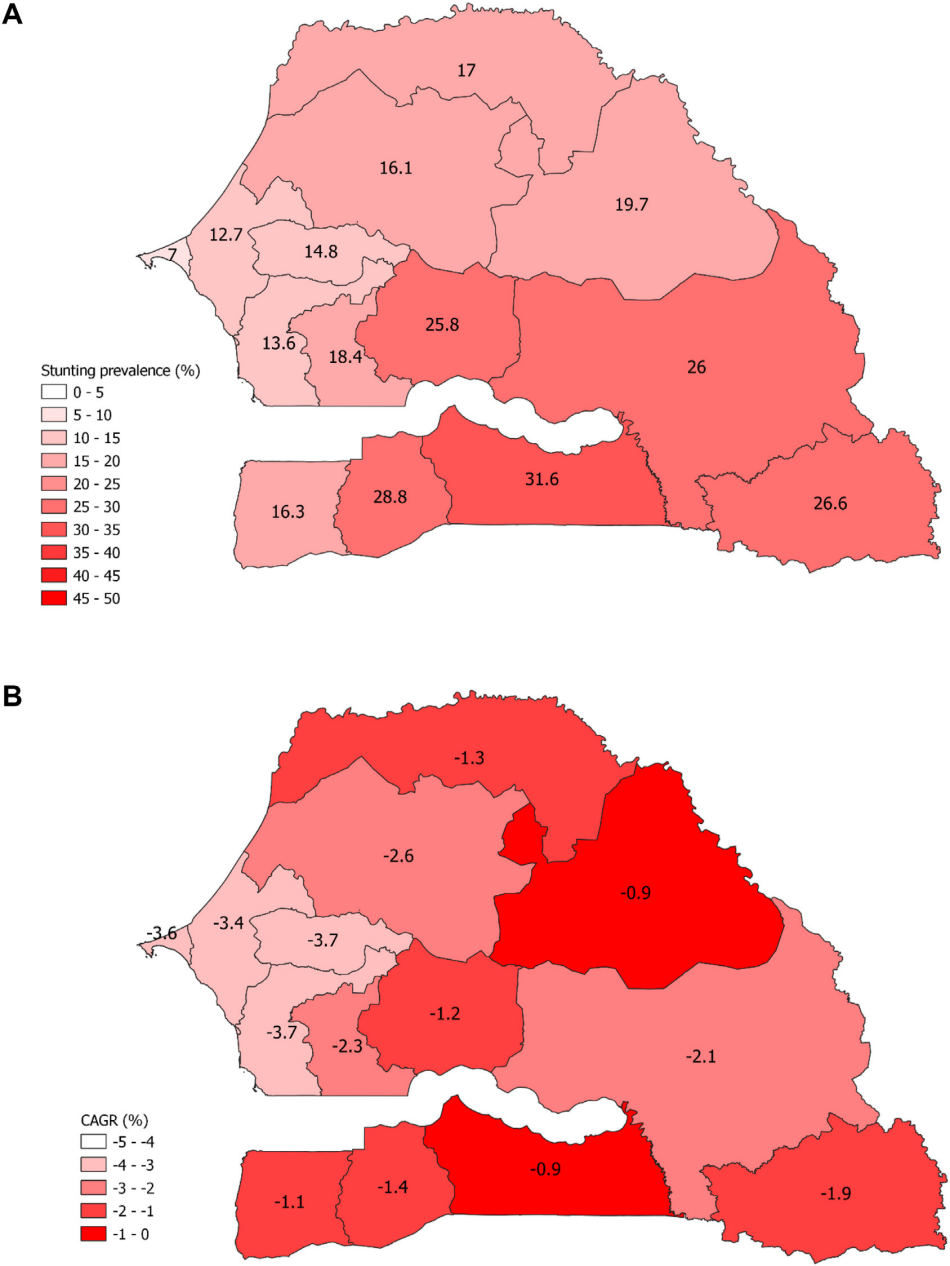
Subnational stunting estimates for children <5 y old in Senegal in 1992/93 (A). CAGR by region, 1992/93–2017 (B). CAGR, compound annual growth rate.

**FIGURE 5:**
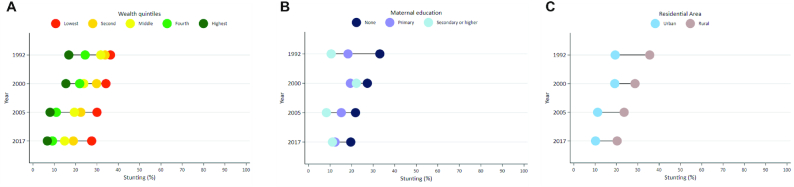
Stunting prevalence by wealth quintile, 1992/93–2017 (A). Stunting prevalence by maternal education, 1992/93–2017 (B). Stunting prevalence by residential area, 1992/93–2017 (C).

Subnational variations exist with regard to the annual reduction in the prevalence of stunting, as measured by the CAGR, from 1992/93 to 2017 (**Figure 4**A-B and Supplementary Appendix 5). Fatick had the largest annual reduction in stunting (CAGR −3.7) followed by Diourbel (CAGR −3.7) and Dakar (CAGR −3.6). The poorest performing regions were Matam (CAGR −0.9), Kolda (CAGR −0.9), and Zinguinchor (CAGR −1.1). Equity analyses examined disparities in stunting reduction among wealth quintiles, maternal education groups, residential areas (**Figure 5**A-C), gender, and wealth quintiles by residential area (Supplementary Appendix 5) between 1992/93 and 2017. Inequalities across wealth quintiles remained stagnant, with a 19.6% gap in the prevalence of stunting between the poorest and richest wealth quintiles in 1992/93 and a 20.8% gap in 2017. Measures of absolute and relative socioeconomic inequalities, SII and CIX, revealed that wealth discrepancies increased over time, with the wealthiest making faster progress in reducing the burden of stunting (Supplementary Appendix 5). When disaggregated by urban and rural residence, it was found that wealth inequalities were largest among the rural population. Children of mothers with no education have made the greatest reductions in the prevalence of stunting, and the gap between children whose mothers have secondary or higher education and no education decreased from 22.6% in 1992/93 to 8.5% in 2017. Urban and rural disparities have decreased from 16% in 1992/93 to 10% in 2017, with children living in rural areas making greater progress. No meaningful differentials exist by child gender, and these have not changed from 1992/93 to 2017.

#### Multivariable Analyses

**FIGURE 6:**
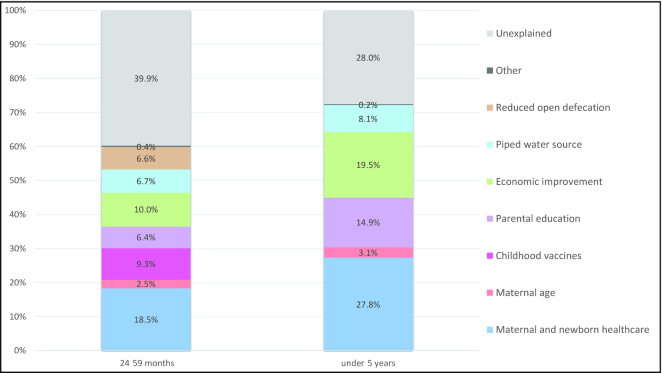
Decomposing predicted changes in HAZ (i.e., percentage contribution of determinant domains) for children <5 y old from 1992/93–2017. *The 6–23-mo age category results are not presented due to the model results being unstable and only explaining 0.21 SD decline in HAZ and the <6-mo old group due to small sample sizes. *Indicators included maternal and newborn healthcare (SBA and ANC ≥4), parental education (maternal and paternal education), reduced open defecation, maternal age (adolescent pregnancy, <18 years of age), economic improvement (wealth index), childhood vaccines (DPT3 vaccine), and piped water source. *Other category includes child age, gender, and region for all age groups in addition to reduction in household crowding (0.4%) for the 24–49-mo old age group. *Parental education breakdown: children 24–59 mo: maternal: 4.0%, paternal: 2.4%; and children <5 y old : maternal: 7.5%, paternal: 7.4%. ANC, antenatal care; DPT3, diphtheria, pertussis, and tetanus; HAZ, height-for-age *z* score; SBA, skilled birth attendant.

Decomposition of the determinants of stunting decline between 1992/93 and 2017 for children 24–59 mo and <5 y old explained 60.1% and 72.0% of child HAZ gains, respectively (**Figure 6**). Ranking of coefficients indicated that the largest contributor of child HAZ improvement between 1992/93 and 2017 was maternal and newborn child health [use of skilled birth attendant (SBA) and 4+ antenatal care (ANC) visits] predicting 18.5% and 27.8% for children 24–59 mo and children <5 y old, respectively (Supplementary Appendix 5). Other factors found to contribute to the change in HAZ in both age groups (24–59 mo and <5 y) included economic improvement (10.0%, 19.5%); parental education (6.4%, 14.9%), with a greater importance placed on maternal education; having access to a piped water source (6.7%, 8.1%); and maternal age (2.5%, 3.1%). Childhood vaccines (DTP3) (6.3%), reduction in open defecation (6.6%), and reduction in household crowding (0.4%) only contributed to the change in HAZ for children between 24 and 59 mo old. Results were not presented for children between 6 and 23 mo old due to minimal changes in HAZ and for children <6 mo of age due to sample size limitations.

A multivariable mixed-effect regression model was conducted to complement the decomposition analysis for the following age groups: <6 mo, 6–23 mo, 24–59 mo, and <5 y. Factors found to influence child HAZ in ≥1 age group included presence of an SBA, 4+ ANC visits, proportion of mothers <18 y old, interpregnancy interval, total number of children, maternal age, acute child respiratory infections, open defecation, wealth index, and urban living (Supplementary Appendix 5).

#### Policy and Program Review

**FIGURE 7:**
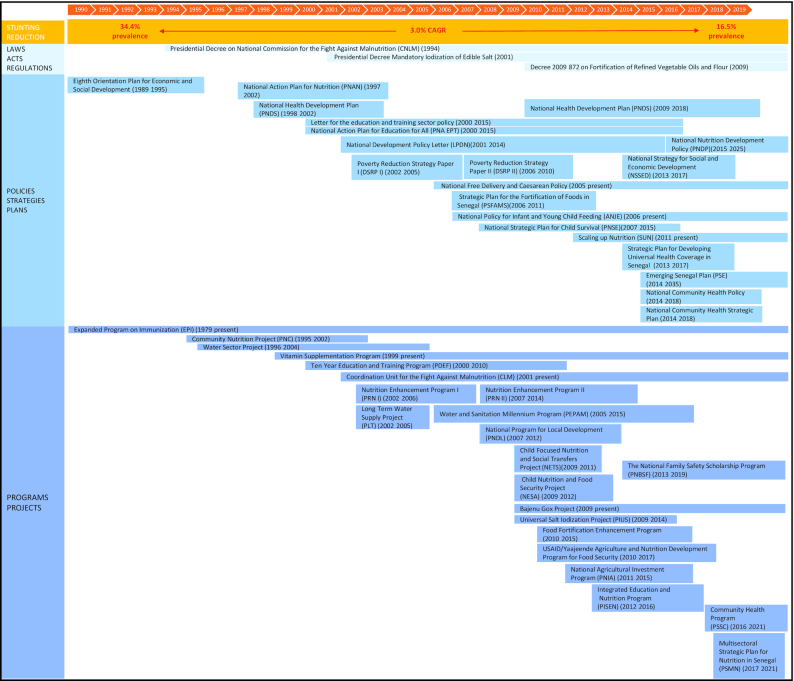
Overview of laws, policies, programs, and enablers between 1990 and 2019 in Senegal.

A comprehensive list of nutrition-specific and -sensitive policies identified between 1990 and 2018 is shown in **Figure 7** and **Panel 2** (41, 59–65). These programs and policies were summarized and ranked based on their impact on stunting reduction (**Supplementary Appendix 6**). Senegal made major strides in improving health sector development, organizing poverty reduction initiatives, implementing campaigns for improving population education, and safe water and sanitation projects. The government's early adoption of coordinating bodies for nutrition and multisectoral action plans for fighting malnutrition [e.g., the 2001 Cellule de Lutte Contre la Malnutrition, Nutrition Enhancement Program (PRN)] have also been pivotal.

Panel 2 Policy and Program Review

Following the 1994 devaluation of the CFA franc and the cumulative effects of climate shocks, the nutrition situation in the country was troublesome (53). This led to the government prioritizing nutrition by creating the Commission Nationale de Lutte contre la Malnutrition (CNLM) which led to the creation of the Community Nutrition Program (PNC) from 1995–2001. The PNC was largely a World Bank-funded program managed by the Agency for Public Works and Employment (AGETIP) (54). This community-based program targeted children under-3 and pregnant and lactating women in urban areas through growth monitoring, promotion of health services, nutrition education, food supplements, and installing water stand pipes. Due to its high costs, the PNC was not sustainable, however it helped improve coordination across multiple sectors (55). In 2001, the Cellule de Lutte Contre la Malnutrition (CLM) replaced the CNLM and further facilitated making nutrition a priority and fostering multisectoral action in Senegal's fight against malnutrition. The CLM was responsible for overseeing a number of large-scale programs including the Nutrition Enhancement Program (PRN); salt iodization, food fortification, and cash transfer programs (55). The PRN (2002–2014) was created to achieve the objectives of the Nutrition Development Policy Letter (57). The PRN targeted children in urban and rural areas nationally through community-based interventions and aimed to strengthen the country's institutional and organizational capacity for implementing and evaluating nutrition programs. This program effectively achieved its targets for community based interventions which were found to decrease the risk of underweight children (41, 58, 59).

The health sector's performance, sustainability and governance were targeted by the National Health Development Plan (60). Key poverty reduction strategies and programs (e.g., the Poverty Reduction Strategy Paper I & II and National Strategy for Social and Economic Development) and other nutrition-sensitive efforts (e.g., the National Action Plan for Education for All; the Water Sector Project, the Long-Term Water Supply Project, and the Water and Sanitation Millennium Project) have targeted vulnerable populations through the establishment of infrastructure, systems, and behaviours at the community-level.

#### Qualitative Inquiry Results

The qualitative analysis highlights diverse contextual and distal policy and program efforts and underlying and immediate causes of stunting decline from the perspectives of national and regional stakeholders and mothers, including improvements in political context and stability, women's empowerment and education, poverty reduction, household access to clean water and sanitation, and improved food security and dietary intake related to greater household income. See **Panel 3**for a summary of the qualitative findings. A full report of the qualitative interviews and analyses can be found in **Supplementary Appendix 7**.

Panel 3 Qualitative Inquiry Results

**National Expert Stakeholders**

National stakeholders credited political stability and lack of large-scale conflicts, moderate poverty reduction, and women's education and empowerment to facilitating improvements in chronic child malnutrition. Political will through the creation of the CNLM and the CLM leading to multisectoral action was a key driver of stunting reduction.

“...The government set up since 200[1] a structure to promote a multi-sectoral approach in the fight against malnutrition. This unit brings together all the technical ministries that have a role to play in the fight against the determinants of malnutrition. This structure is highly set up at the level of the Prime Minister's Office, which facilitates the coordination of sectoral interventions. So the state of Senegal has [from] very early [on] understood the advantage of this multi-sectoral approach to the fight against malnutrition...” [CLM representative]

The PNC, led by the CNLM, the PRN, iodized salt program and the food security program (NESA), led by the CLM, were central programs that reduced undernutrition. Several nutrition sensitive policies and programs facilitated cross- and multi-sectoral collaboration including the education, health, and hydraulics sectors. Support from bilateral and multilateral donors for nutrition programs/projects was integral in the success in reducing stunting. However, challenges in the implementation of programs included: inequitable coverage and access to programs, competition and dynamics between actors and institutions, limited monitoring and accountability of community actors, limited involvement of local authorities, limited sustainability, inadequate support and training for nutrition experts, challenges with health promotion and social behaviour change communication, and failure of donors to align with national priorities. The country has had moderate improvements in access to basic resources including drinking water and sanitation facilities, access to health services through the role of community health workers and their promotion of exclusive breastfeeding, and training and availability of nutrition experts. Consequently, decreased fertility, increased spacing between births and the reduction of communicable infections were expressed as important factors that led to stunting gains with further efforts required to address micronutrient deficiencies including iodine deficiency and anemia.

**Regional Stakeholders**

Regional stakeholders agreed with the national stakeholders on the positive effects of poverty reduction, maternal education and programs such as the CLM, the PRN and other donor-led initiatives on stunting reduction. They also indicated improvements in access to health services and hygiene practices such as handwashing.

"There are families who know and apply the rules of hygiene inside the house. There are others who apply little or no hygiene, but there have been changes in the meantime." [Health Post, Dialene, Kaolack]

However, cultural practises and poverty have limited progress towards diet diversity and exclusive breastfeeding despite recommendations provided by health workers.

“Most moms do not ask for advice about breastfeeding. This is due to their ignorance. On the other hand, young mothers start asking questions about exclusive breastfeeding, because they have a higher level of education.” [ASBEF NGO Supervisor]

**Mothers in Communities**

Focus groups with mothers revealed poverty reduction as essential to food security which has seen some improvements. Although the availability of food has improved, it is dependent on geographic location and seasonality. However, challenges with water, sanitation and hygiene were observed across the three regions and age groups.

“We cannot have good health without good sanitation, also in terms of food it is difficult for us to find fruits and vegetables, garbage in the street is also a problem, it is difficult to find fruit. The salinization of water is [also] a problem in this region.” [Mother, FGD 1992-1997 Diourbel, urban]

Mothers mentioned improvements in the availability of health posts as most had received 4+ ANC visits, had SBA deliveries, had their children vaccinated, and had nutrition-related advice provided to them.

“The diet has improved because during immunization sessions, you are advised the foods that must be given to the child.” [Mother, FGD 1992-1997, Kaolack]

## Discussion

### Summary

Using a mixed-methods approach, in this study we identified factors related to Senegal's stunting decline in transitionary periods such as 1992/93–2005, when stunting declined significantly, to more stagnant periods such as from 2005 to 2017. To begin, the political stability experienced by Senegal fostered an environment supportive of health and nutrition policies. Specifically, as early as 1994, the government demonstrated the political will to institutionalize nutrition and have multiple sectors involved with the creation of the CNLM. Improvements in community health service availability, maternal education through increased primary and secondary school enrollment, access to piped water and sanitation facilities, and poverty reduction have been observed over our study period and supported the reduction in stunting.

### Strengths and limitations

This mixed-methods study, to our knowledge the first of its kind performed in Senegal, incorporated robust quantitative analyses; multilevel perspectives from qualitative interviews and focus groups; an in-depth program and policy review; and the first systematic review of existing literature. A number of our study strengths were attributed to the quantitative methods. First, combining the DHS and MICS surveys allowed for an analysis over periods of peaceful democratic transitions (i.e., 2000–2012), political commitment toward achieving the Millennium Development Goals (MDG), and substantial prioritization of cross- and multisectoral nutrition-focused efforts. Second, our use of piecewise linear splines on the Victora growth curves allowed for identification of inflection points and comparison of the rate of HAZ change per month across age groups and time periods. Third, a comprehensive set of predictors of stunting were included, allowing us to account for possible pathways in the hierarchal regression models. However, the effects of food security, agriculture, intrauterine growth, maternal height and BMI, and nutrition and health programs could not be quantitatively examined due to data unavailability for our full time period. Proxies were used where possible, i.e., 24-hour recall was used to measure food security. A post hoc decomposition analysis revealed that maternal height contributed to nearly 6% of the explainable (57%) stunting decline from 1992/93 to 2005, suggesting that maternal nutrition may also have an important role. It should be noted that although a wide range of confounders were controlled for, there were possibilities for residual confounding and interactions between factors within and between hierarchal levels in our decomposition models. Last, the DHS 2010/11 could not be used in our analysis due to poor data quality, as there were >20% flagged implausible values for child anthropometry data. Accordingly, we used the most recent Senegal DHS (2017) as an endpoint for our decomposition analysis, which has not previously been used. To increase our confidence in the surveys we used, the quality of anthropometry data was rigorously examined for accuracy of height and age data (see paper on quality metrics in the Supplement).

### Existing evidence

Senegal's overall political stability has ensured a stable and secure context, making the country attractive to bilateral donors for nutrition, health, and agriculture programs (66). Only one small scale conflict has been documented, the Casamance conflict, involving the southwestern regions. This conflict led to declines in agricultural production, destruction of infrastructure and livelihood assets (e.g., due to mines in fields and orchards), and increased poverty in the Zinguinchor region (9, 67). The results of our subnational equity analysis found that the southern regions had the highest prevalence of stunting between 1992/93 and 2017 and made the slowest progress in stunting reduction, particularly in Kolda. These results are supported by the literature, where higher food insecurity and a higher prevalence of stunting have been observed in the southern regions compared with their counterparts (9, 68, 69).

The evolution of nutrition policy in Senegal has occurred over the following series of phases: *1*) establishing a foundation (1950–1970s); *2*) implementation of a curative approach (1970–1990s); *3*) institutionalization of nutrition (1990–2000s); *4*) intensification and decentralization (2000–2010s); and *5*) adoption of a multisectoral approach (2010–present) (61). Nutrition became a national priority through the initiation of the CNLM and its reform, the CLM. These organizations played a key role in reducing chronic malnutrition by implementing and monitoring major nutrition initiatives such as the PNC and the PRN and promoting multisectoral action (61). Despite the financial and technical support of donors, the Senegalese government created a nutrition-specific budget line in 2001, resulting in an increase from 0.02% of the national budget in 2000 to 0.12% in 2015 directed toward nutrition programs (70). The results of a national study reinforced the certainty that the role of the Senegalese government in prioritizing nutrition was essential to Senegal's success story in reducing stunting (71).

Access to and use of reproductive, maternal, newborn, and child health (RMNCH) services have remarkably improved from 1992 to 2017, with a 4-fold increase in the proportion of women attending 4+ ANC visits and a slightly smaller increase in SBA births (11). These improvements may in part be attributed to the National Health Development Plan, which improved health system performance and integrated reproductive health services (65, 72). Additionally, there has been an increasing number of community health huts since 1977, funded by USAID, providing a range of basic maternal and child health services, including immunizations and growth monitoring (73). Our analysis revealed that maternal and newborn healthcare and childhood vaccines largely contributed to the stunting decline. The beneficial effects of ANC visits, as mentioned by mothers in focus groups, was that they presented an opportunity to receive advice related to infant and child nutrition and dietary diversity. A previous national study supported our findings of the importance of 4+ ANC visits and being born in a medical facility contributing to a large part in the stunting decline from 1993 to 2011 (12). No studies in Senegal explored the positive effects of vaccines on chronic malnutrition in children; however, vaccination coverage has increased since the early 1990s due to free vaccines provided by the Expanded Program on Immunization (74).

Female literacy has modestly improved and may be attributed to several national efforts, including the National Action Plan for Education for All, the Letter for the Education and Training Sector Policy, and the Ten-Year Education and Training Plan (11). Children of educated mothers are less likely to be stunted since they may have had higher rates of exclusive breastfeeding, appropriate antenatal and postnatal care, adequate vaccinations, appropriate diarrhea and acute respiratory infection care, and better access to clean water, sanitation and, hygiene sanitation (WASH) services. Additionally, maternal education supports women's empowerment, lowers fertility rates, decreases adolescent pregnancies, and decreases the risk of early marriage. We found maternal and paternal education and decreased early age of pregnancies led to improvements in child growth. Findings from previous studies supported the relationship between improvements in parental education and decreased child stunting in Senegal, though rural subnational studies found no evidence of an association, likely due to low literacy rates in rural areas (12, 20, 21, 25, 33, 34). Reductions in stunting were particularly prominent among the least educated mothers who had much scope for improvement and were also targeted in government community-based programs.

Senegal has had moderate improvements in sanitation facilities and access to piped water over the past few years, achieving the MDG target of 7.c by focusing on larger population centers. WASH initiatives in the country led to improved management and coverage of potable water, and in more recent years, the sewer system. In our study, improved access to piped water and reduction in open defecation contributed to the stunting decline between 1992/93 and 2017; and an earlier study of stunting determinants between 1993 and 2011 also found piped water to be a key driver of change (12). The relative importance of WASH interventions to stunting can be explained largely by reduction in infectious disease (such as diarrhea and pneumonia) that likely occurred from cleaner water and sanitized environments. Literature in low- and middle-income countries has also linked better WASH conditions to stunting through direct biological mechanisms and indirect social and economic mechanisms (75). Still, the unequal targeting of WASH initiatives in rural Senegalese communities has led to concerns regarding lack of access to improved toilet facilities, local waste disposal, and safe drinking water at the community level.

Senegal has experienced steady GDP growth following the devaluation of the CFA franc and a reduction in the poverty headcount ratio at national poverty lines ($1.90 per d) from 68% (1991) to 38% (2011) (11). Remittances due to labor migration, largely from Europe and the United States, have played a key role in improving the socioeconomic conditions of households, with most of the income being spent on food, education, and healthcare (76). Poverty reduction has been a national priority through the implementation of the Poverty Reduction Strategies, which aimed to achieve the MDGs. Improvement in household wealth has reduced the burden of stunting, with other national studies supporting our finding while subnational studies failed to find an association (12, 14, 26, 33, 37). This difference may be attributed to a lack of variation in household wealth in subnational studies and considerable gaps in the prevalence of stunting between the poorest and richest wealth quintiles widening over time in Senegal.

### Remaining challenges

Inequalities in the prevalence of stunting have persisted across wealth quintiles, maternal levels of education, and area of residence. Children from the wealthiest families, those whose mothers have secondary or higher levels of education, and those living in urban areas are less likely to be stunted than their counterparts.

While the health system in Senegal has been improving, challenges in equitable targeting of resources remain. The 1996 decentralization reform (Law 96-06 on the local government code) led to the transfer of financial resources, responsibility, and autonomy for 9 policy areas, including education and health (77, 78). However, the transfer of power to local governments may have been limited due to the inequitable or inadequate distribution of financial resources (78). Mandatory and voluntary health insurance and subsidized health services for vulnerable populations only cover a small subset of the population (23–28%), leading to financial challenges in accessing health services for the majority of the poor and rural populations (78). Additionally, the low density of health providers has also led to inequalities in health services provision in poor rural areas. The country is currently aiming to improve access to health services for all populations through developing a universal health coverage program (79).

Senegal is highly reliant on agricultural production; however, early agriculture programs have struggled to meet their targets and have not effectively contributed to the long-term growth of the sector (69). Recent efforts to improve resilience strategies to protect against climate shocks that cause food insecurity should be expanded upon and improved to benefit vulnerable populations. Child wasting, as it is linked to acute food shortages and the environment, is also high at the national level (9%) and has been a challenge for several decades, particularly in some regions. The government should prioritize interventions to mitigate premature death and concurrent stunting and wasting in these regions.

## Conclusion

Senegal's success in reducing the burden of stunting is credited to the enduring prioritization of nutrition in national development plans, implementation of national nutrition-specific and -sensitive interventions, and the targeting of the most vulnerable populations. The execution of a multisectoral approach has been essential in facilitating the prioritization, coordination, and implementation of nutrition efforts across diverse sectors and corresponding stakeholders. Future studies may focus on subnational drivers of stunting reduction, examine other outcomes such as wasting occurring concurrently with stunting, and perform cost-effectiveness analysis of programs to determine where future investments would be useful.

## Supplementary Material

nqaa151_Supplemental_File

## Notes

Funded by a grant to the Centre for Global Child Health from Gates Ventures. The funder had no role in the design, implementation, analysis, or interpretation of the data.

Data described in the manuscript, code book, and analytic code will be made available upon request pending application and approval.

KC to JW authors organized alphabetically.

Supplementary Appendix 1–7 are available from the “Supplementary data” link in the online posting of the article and from the same link in the online table of contents at https://academic.oup.com/ajcn/.

Abbreviations used: ANC, antenatal care; CAGR, compound annual growth rate; CIX, concentration index; CLM, Cellule de Lutte Contre la Malnutrition; CNLM, Commission Nationale de Lutte Contre la Malnutrition; DHS, demographic and health surveys; GDP, gross domestic product; HAZ, height-for-age *z* score; MDG, millennium development goal; MICS, multiple indicator cluster survey; NGO, nongovernmental organization; PRN, nutrition enhancement program; PNC, community nutrition program; SBA, skilled birth attendant; SII, slope index of inequality; WASH, water, sanitation and hygiene.

**Disclosure Statement: Published in a supplement to the American Journal of Clinical Nutrition. The Guest Editor for this supplement was Mark Manary, and has no disclosures. Publication costs for this supplement were defrayed in part by the payment of page charges by Gates Ventures. The opinions expressed in this publication are those of the authors and are not attributable to the sponsors or the publisher, Editor, or Editorial Board of the *American Journal of Clinical Nutrition*.

The Supplement Coordinator for the supplement publication was Nadia Akseer, Gates Ventures/Hospital for Sick Children, Toronto, Canada. Supplement Coordinator disclosure: no conflicts to disclose. The Stunting Exemplars research Principal Investigator was Zulfiqar A Bhutta, Hospital for Sick Children, Toronto, Canada. Principal Investigator disclosure: no conflicts to disclose.
