# Supplementary material for: Drivers of stunting reduction in Senegal: a country case study
Source: Am J Clin Nutr. 2020 Aug 10;112(Suppl 2):860S–874S. doi: 10.1093/ajcn/nqaa151 (PMC7487429; doi:10.1093/ajcn/nqaa151)
Supplement: nqaa151_Supplemental_File [file nqaa151_supplemental_file.docx]

Drivers of stunting reduction in Senegal: a country case study

Authors: Samanpreet Brar, Dr. Nadia Akseer, Dr. Mohamadou Sall, Kaitlin Conway, Ibrahima Diouf, Karl Everett, Muhammad Islam, Papa Ibrahima Sylmang Sène, Hana Tasic, Jannah Wigle, Dr. Zulfiqar Bhutta

**Online Supplementary Material**

**List of Appendices**

[**Supplementary Appendix 1:** Descriptive Analysis of Contextual Factors 4](#_Toc38052045)

[**Supplementary Appendix Figure 1A:** Trends in GDP per capita, poverty and urbanization, 1990-2017 4](#_Toc38052046)

[**Supplementary Appendix Figure 1B:** Trends in key contextual indicators, 1995-2017 4](#_Toc38052047)

[**Supplementary Appendix 2:** Systematic Literature Review 5](#_Toc38052048)

[**Supplementary Appendix Figure 2**: Literature review flow diagram 6](#_Toc38052049)

[**Supplementary Appendix 3:** Multivariable Analyses Methods 26](#_Toc38052050)

[**Supplementary Appendix 4:** Qualitative Data Collection and Analyses Methods 29](#_Toc38052051)

[**Supplementary Appendix Table 1:** Inclusion Criteria 30](#_Toc38052052)

[**Supplementary Appendix Figure 3**: Regional map of Senegal displaying study sites for subnational key informant interviews and focus group discussions. 31](#_Toc38052053)

[**Supplementary Appendix 5:** Quantitative Results 33](#_Toc38052054)

[**Supplementary Appendix Figure 4A**: Spline analysis of inflection points of change in the slope of HAZ, 1992/93 33](#_Toc38052055)

[**Supplementary Appendix Figure 4B**: Spline analysis of inflection points of change in the slope of HAZ, 2000 33](#_Toc38052056)

[**Supplementary Appendix Figure 4C**: Spline analysis of inflection points of change in the slope of HAZ, 2005 34](#_Toc38052057)

[**Supplementary Appendix Figure 4D**: Spline analysis of inflection points of change in the slope of HAZ, 2017 34](#_Toc38052058)

[**Supplementary Appendix Figure 5A**: 2000 stunting prevalence by region 35](#_Toc38052059)

[**Supplementary Appendix Figure 5B**: 2005 stunting prevalence by region 35](#_Toc38052060)

[**Supplementary Appendix Figure 5C**: 2017 stunting prevalence by region 36](#_Toc38052061)

[**Supplementary Appendix Figure 6A:** Stunting prevalence by gender, 1992/93 – 2017 36](#_Toc38052062)

[**Supplementary Appendix Figure 6B**: Stunting prevalence by residential area and wealth quintiles, 1992/93 – 2017 37](#_Toc38052063)

[**Supplementary Appendix Figure 7A**: Change in absolute SII by year in Senegal 37](#_Toc38052064)

[**Supplementary Appendix Figure 7B**: Change in relative CIX by year in Senegal 38](#_Toc38052065)

[**Supplementary Appendix Table 2:** Descriptive trends in stunting determinants in 1992/93 - 2017 in children <5 years 39](#_Toc38052066)

[**Supplementary Appendix Table 3:** Decomposition analysis for children among <5 years from 1992/93-2017 44](#_Toc38052067)

[**Supplementary Appendix Figure 8A:** Decomposing predicted changes in HAZ among children <5 years (i.e. relative ranking of product coefficients for determinant domains) from 1992/93-2017 44](#_Toc38052068)

[**Supplementary Appendix Table 4:** Decomposition analysis for children among <24 months from 1992/93-2017 45](#_Toc38052069)

[**Supplementary Appendix Figure 8B:** Decomposing predicted changes in HAZ among children <24 months (i.e. relative ranking of product coefficients for determinant domains) from 1992/93-2017 45](#_Toc38052070)

[**Supplementary Appendix Table 5:** Difference-in-differences multivariable regression for children under-5 years from 1992/93 - 2017 46](#_Toc38052071)

[**Supplementary Appendix Table 6:** Difference-in-differences multivariable regression for children aged 24-59 months from 1992/93 - 2017 54](#_Toc38052072)

[**Supplementary Appendix Table 7** Difference-in-differences multivariable regression for children aged 6-23 months from 1992/93 - 2017 60](#_Toc38052073)

[**Supplementary Appendix Table 8**: Difference-in-differences multivariable regression for children under 6 months from 1992/93 - 2017 66](#_Toc38052074)

[**Supplementary Appendix 6:** Program and Policies 72](#_Toc38052075)

[**Supplementary Appendix Table 9:** Description of Acts/Law/Regulations, Policies, and Program from 1990-Present 72](#_Toc38052076)

[**Supplementary Appendix 7:** Qualitative Results 87](#_Toc38052077)

[**Supplementary Appendix Table 10A**: Summary of national stakeholders 88](#_Toc38052078)

[**Supplementary Appendix Table10B**: Summary of regional stakeholders 101](#_Toc38052079)

[**Supplementary Appendix Table10C:** Summary and comparison of mothers in communities 108](#_Toc38052080)

[**Supplementary Appendix References** 118](#_Toc38052081)

# **Supplementary Appendix 1:** Descriptive Analysis of Contextual Factors

## **Supplementary Appendix Figure 1A:** Trends in GDP per capita, poverty and urbanization, 1990-2017

Source: (1)

## **Supplementary Appendix Figure 1B:** Trends in key contextual indicators, 1995-2017

Source: (1–3)

# **Supplementary Appendix 2:** Systematic Literature Review

**Methods**

Using the search categories of 1) stunting (e.g. stunting, linear growth, linear growth stunting, HAZ, height, height-for-age, LAZ, length, length-for-age, undernutrition, malnutrition, nutr*), 2) child (e.g. child*, infan*), and 3) Senegal (Senegal*), we searched more than 15 online databases and grey literature sources. We searched: MEDLINE, Embase, AMED, CAB Abstracts, CINAHL, Cochrane CENTRAL, Campbell Collaboration, EPPI Centre Trials Register (TRoPHI), 3ie, JOLIS, African Journals Online, WHOLIS, LILACS, Scopus, and Web of Science. Additional searches for grey literature were conducted using Google, a hand search of reference lists of relevant reviews, and directly searching organizational websites including national, regional and headquarter websites for UNICEF, WHO, UNDP, WFP, FAO, World Bank Group Open Knowledge Repository, Nutritional International, Global Alliance for Improved Nutrition, International Food Policy Research Institute, Government of Senegal including the Ministry of Health and Social Action, and Ministry of Agriculture and Rural Development.

The exported set of records were de-duplicated and screened for relevance. Records were included if they met all of the following inclusion criteria:

i) included an under-5 population in Senegal;

ii) published between 1990-2017;

iii) examined one or more of the determinants of chronic undernutrition (e.g. determinants, risk factors, policies, programs, interventions, or initiatives); and

iv) examined effects on child growth or a reduction in stunting

Of the 2,718 articles initially found in this process, 44 were ultimately included in the literature review based on title/abstract and full-text screening. This included 40 published peer-reviewed articles and 4 pieces of grey literature. Additional targeted searches were completed for more information on key topics to supplement and expand on important factors in the Senegal stunting narrative (**Supplementary Appendix Figure 2**).

## **Supplementary Appendix Figure 2**: Literature review flow diagram


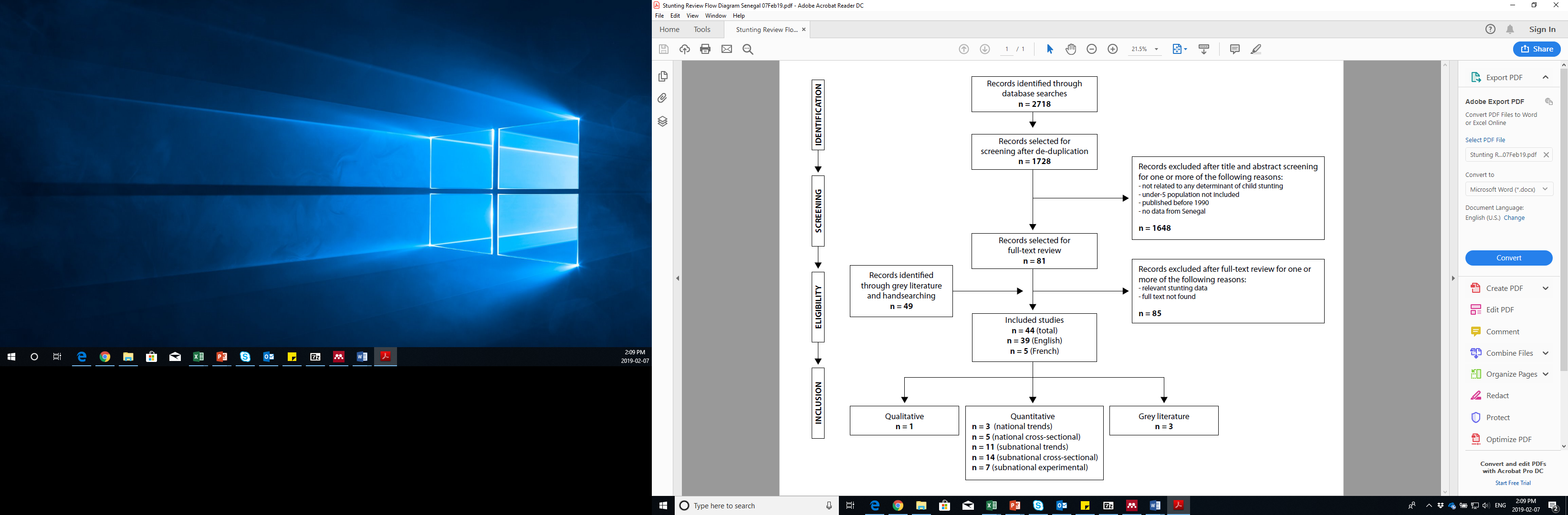


**Full Literature Review**

Factors that have contributed to a reduction in stunting in Senegal are presented in relation to an adapted version of UNICEF’s conceptual framework for malnutrition (4). Over the last several years, key events such as the of the introduction of the CLM, poverty reduction through remittance incomes, improvements in water and sanitation, health service decentralization, and health system strengthening have targeted the basic, underlying and immediate causes of malnutrition in children. Along with this, an increase in coordinated, multi-sectoral nutrition-specific and nutrition sensitive policies and programs have allowed for a more focused approach to improving the nutritional status. The aim of this literature review is to summarize, systematically, the existing evidence on determinants of child stunting decline in Senegal.

**Basic causes (Distal)**

*Environmental Context and Social Demographics*

Climate Shocks

Senegal is bounded by the Atlantic Ocean, Mauritania, Mali, Guinea, and Guinea-Bissau and completely surrounds The Gambia (5). It is divided up into five climatic zones: sahelian, sahelo-sudanian, sudanian, sudano-guinean, and Guinean (6). The sahelian zone is made up of a tree or shrub steppe and contains the north of the Saint-Louis region. The sahelo-sudanian zone is made up of a tree and dry steppe and is made up of the regions of Dakar, Thiès, Diourbel, Louga, and Matam. The sudano-guinean zone is made up of forests and very thick savannas and includes the north of Ziguinchor and Kolda and the south of Tambacounda. The sudanian zone has a savanna landscape and includes the regions of Fatick and Kaolack and the north and center of the Tambacounda region. Lastly, the Guinean is the most humid zone and is made up of the north areas of Ziguinchor and the Kolda region. Senegal is also divided into seven agro-ecological zones: the Senegal valley, the Niayes, Northern Groundnut Basin, Southern Groundnut Basin, the Sylvopastoral Zone, Eastern Senegal and Upper Casamance, and Lower and Middle Casamance (6). Agriculture is one of the main contributors to the GDP, contributing to 15.4% of GDP and 53.4% of employment in 2017 (7,8). The major food staples include millet, sorghum, maize, and rice. Cereal crops are mainly imported, while cotton and groundnut are the major exports. Senegal is prone to climate shocks including heat waves, droughts, and floods which all diminish agricultural production. Nutrition status measured using a variety of anthropometric measures has been found to vary between seasons where it declines at the end of the rainy season for farmers and at the end of the dry season for pastoralists in West Africa (9). Along with climate shocks, economic shocks also cause food insecurity throughout the country. In early 2008 and from 2009-2011, periods of increased domestic and international food prices caused acute food shortages and resulted in food insecurity across Senegal (10). In the latter period, there were significant increases in food purchase prices, loss of key income sources, and droughts which further compounded food insecurity (p<0.01). Additionally, the mean height-for-age (HAZ) of children (12 to 60 months of age) living in rural households in 8 regions of Senegal significantly decreased from 2009 (n=1694) to 2011 (n=2116) (difference: 0.26, p<0.01). The authors assessed the effects of economic and climate shocks on child HAZ and stunting. They found increases in purchase prices (b=0.30, p<0.01) increased child HAZ while extreme cold (b=-0.80, p<0.01) and decreases in sale prices, i.e. the price at which the farming households sell their products, (b=-0.71, p<0.05) decreased child HAZ. No evidence was found of droughts (b=-0.18), crop pests (b=-0.18), loss of key income sources (b=0.39) and the death of productive (b=0.46) and unproductive household members (b=0.04) affecting child HAZ. The authors also examined interactions between economic and climate shocks with the year 2011 to account for structural improvements such as nutrition and agricultural programs that improved between survey rounds. They found that despite structural improvements, droughts had a negative effect on child HAZ (b=-2.33, p<0.05). Interaction terms between increased prices (b=0.25) and increased prices and drought combined (b=0.96) with the year 2011 were not significant predictors of child HAZ. An interaction between droughts and increased prices was also not associated with child HAZ (b=-0.65). Results for the binary outcome of stunting were similar where extreme cold (b=0.14, p<0.05) and decreases in sale prices (b=0.15, p<0.05) increased the risk of stunting while increases in purchase prices (b=-0.05, p<0.05) decreased the risk of stunting. Droughts (b=0.03), crop pests (b=0.01), loss of key income sources (b=-0.10), the death of productive (b=-0.04) and unproductive household members (b=-0.01) were not associated with the risk of stunting. Interactions between the year 2011 and increased prices (b=-0.04), drought (b=0.21), increased prices and drought combined (b=-0.13), and an interaction between increased prices and droughts (b=-0.28) were also not associated with the risk of stunting. In summary, the results of this study suggest climate shocks including extreme cold and droughts may increase the risk of chronic child malnutrition. Economic shocks such as decreases in sale prices may increase the risk of stunting as farming households’ incomes are reduced (10).

Urbanization

Senegal is slowly becoming more urbanized, though only having a 12% increase in urban areas from 1990 to 2017. People living in urban areas tend to be higher on the socioeconomic hierarchy as compared to their rural counterparts. This may be attributed to a number of factors including better access to health services, schools, and diversity of foods. National data collected by the DHS and the MICS have shown the prevalence of stunting in urban areas to be less than that of rural areas. Using DHS data (1993-2014), living in rural areas was found to decrease HAZ (b=-0.11, p<0.01) and increase the odds of stunting (b=0.03, p<0.05) but not of severe stunting (b=0.01) of preschool children while adjusting for confounders (11). Findings were similar using the 1997 DHS data where 36.6% of children under the age of 3 were living in urban areas and HAZ scores significantly differed between regions (n=2497) (urban: -0.67, rural: -1.16, p<0.01) (12). Using the 2000 MICS data, a study found the difference in stunting rates (OR: 0.51, 95% CI: 0.45,0.58, p<0.05) and mean HAZ (Urban: -0.73 vs. Rural: -1.16, p<0.05) to significantly differ between regions, with rural areas being at a greater disadvantage for children under the age of 5 (n=8319) (13). No region based differences in HAZ scores existed when comparing children from the same wealth quintile. When accounting for wealth, rural children were no longer at a greater risk of being stunted (OR: 0.92, 95% CI: 0.49,1.70) (13). These findings may infer that children living in rural areas are likely at a disadvantage of chronic malnutrition leading to stunting if they are also experiencing poverty. A subnational study had consistent findings with the national studies when comparing chronic malnutrition in urban and rural areas for children under-5 (14). They compared children living in Pikine, an urban town close to Dakar, in 1986 (n=2049) to those living in a peanut producing rural area (250 km from Dakar) in 1984 (n=3797) and found HAZ scores were worse in the rural area (mean=-0.58, SD: 1.28 and mean=-0.94, SD: 1.33, p<0.0001, respectively) (14). Most studies are in agreement that children from rural areas are at a greater risk of stunting. These region-wide differences with regards to stunting rates may be attributed to dissimilarities in diet and lifestyle factors of children living in rural settings as compared to urban settings.

Household Size

A number of subnational studies assessed the number of household members as predictors of stunting. In the Podor Health District, a cross-sectional study assessed the bivariate association between having a family size greater than 10 and stunting (15). They found no relationship between family size and stunting for children between the ages of 6 and 23 months (n=374) (OR: 0.65, 95% CI: 0.38,1.10, p= 0.1) (15). A study including 110 rural villages in Senegal with households with at least one child under 24 months of age had consistent findings of household size not being associated with child length-for-age (LAZ) (b=0.00, SE: 0.01) (16). In Pikine, the number of living children was also not associated with child HAZ (n=751)(17). However, a study including the regions of Fatick, Kaolack and Kolda found increasing household size to negatively affect HAZ (b=-0.01, SE: 0.003, p<0.01)(18). The findings of these studies may be mixed as the effect of household size on the risk of chronic malnutrition of family members may be dependent on the ages of family members and whether they are able to produce income. In farming households, it may be beneficial to have larger household sizes to help with labour. However, in times of food insecurity caused by climate shocks, urban and rural household may experience equal difficulty with feeding their large families.

*Migration*

Labour migration to send back remittances has been one solution for households to compensate for the food insecurity caused by climate shocks (19). A study looked at drivers of international migration and food security measured using child stunting and wasting as proxies. They found that the odds of international migration was associated with excessive precipitation (OR= 3.37, p<0.01), while food insecurity, heat waves, cold snaps, droughts and the simultaneous occurrence of drought and heat wave months have no statistically meaningful impact (19). An interaction between child wasting and heat waves (OR=0.25, p<0.01), droughts (OR=0.03, p<0.01), and the simultaneous occurrence of drought and heat wave months (OR= 0.05, p<0.001) was found to be associated with international migration. There were no significant interactions between child stunting and climate shocks. These findings suggest that people from areas where there is acute malnutrition are more likely to move but people from areas with chronic malnutrition are not likely to move when they experience heat waves and droughts (19). This may be due to households that are experiencing chronic malnutrition not having the available financial means or the physical capacity to migrate in times of shocks. It is more common for young people with low educational attainment to migrate to the city from rural centers during the dry season and return back home during July to October, the period of field work (20). A study conducted in the rural area of Niakhar examined weather catch-up growth among a sample of youth in 2001 (who were originally recruited when under the age of 5 in 1983-1984) was associated with migration to Dakar, the Capital of Senegal (n=2874). Height increment from preschool age (1 to 5 years) to late adolescence (18 to 23 years) was found to be non-linearly associated with duration of migration to Dakar for only girls. Girls who migrated to Dakar for a short duration of time had the greatest height increments, whereas those who migrated for longer durations had similar height increments as girls who had never migrated; this may be attributed to shorter periods of rest and workloads in the city and corresponding sleep patterns (20). No relationship between age of migration and height increments was found (20). Factors associated with the degree of preschool stunting (none, mild, marked) for youth migrating to all destinations included any migration (%) and age at first migration for both genders, total duration of migration for only females while number of migrations was not associated for both genders. Factors associated with the degree of preschool stunting for youth migrating to Dakar includes any migration (%) for males, age at first migration and total duration of migration for females with number of migrations not being associated for both genders. Sex differences in predictors of stunting may be related to females migrating at a younger age, migrating more often, and for longer durations as compared to males (20). In summary, girls had some gains with catch-up growth when they migrating for short period to an urban area, Dakar, from a rural area in Niakhar but boys were not shown to have great benefits.

*Wealth*

National data collected by the DHS and the MICS have shown the prevalence of stunting to vary by wealth, with the poorest wealth quintiles experiencing the highest rates of stunting. The 2012-2013 DHS showed that children from the poorest wealth quintile were at a 2.3 times greater risk of stunting as compared to those in the richest wealth quintile (21). In the 2000 MICS survey, children from the poorest (OR=3.19, 95% CI: 1.79, 5.78, p<0.05) and middle wealth quintiles (OR=1.93, 95% CI: 1.06, 3.50, p<0.05) were more likely to be stunted as compared to those from the wealthiest quintile (n=8319) (13). A study that examined predictors of stunting reduction found wealth accumulation to be the most important predictor, followed by healthcare and parental education (11). From 1993 to 2014, improvements in asset index (1-10) predicted 24% of the change in mean HAZ and 16% of the change in the prevalence of stunting and was a significant predictor of HAZ (b=0.07, SE: 0.01, p<0.01), stunting (b=-0.01, SE: 0.003, p<0.01), and severe stunting (b=-0.01, SE: 0.002, p<0.01) among a national study of children under-5 (11). A second national retrospective study also found improved asset index to be associated with greater HAZ among under-5 children (b=0.05, SE: 0.01, p<0.01) (22). A 29% change in mean HAZ from 1993 to 2011 (-1.44 to -1.14) was found and 16.1% of the change in HAZ (of the total 66% explained by the model) was attributed to the asset index (22).

Unlike the national studies where there was agreement on the impact of wealth on child HAZ, findings were mixed among subnational studies. In the regions of Fatick, Kaolack and Kolda, data from a 2004 study found wealth index was not associated with child HAZ (b=0.03, SE: 0.02, p<0.10)(18). Findings were consistent in a study in Pikine where wealth index was not associated with child HAZ(17). A study conducted in 110 rural villages in Senegal including households with at least one child under 24 months of age, explored predictors of HAZ for data collected in 2008 to 2009 – they found that children from middle and upper wealth quintiles [third (b=0.36, SE: 0.12, P<0.01), fourth (b=0.40, SE:0.15, p<0.05), fifth (b=0.43, SE:0.17, p<0.05)] as opposed to the poorest wealth quintile had significantly lower LAZ scores (n=1908)(16). This finding suggests no difference between the poorest and second poorest wealth quintiles but families from the middle to the highest wealth quintiles are better able to supply nutritious diets for their infants. A family’s wealth may determine their ability to afford proper housing. In flourishing villages in Senegal, houses may be made up of cement as compared to straw or other unprocessed materials. A study in a rural area of Senegal examined the association between quality of housing (mud-bricks vs. cement) and chronic child malnutrition (23). They found no evidence that the quality of housing improves HAZ or linear growth among 12 to 42 month old children (n=500) (23). In summary, wealth is likely to play a role in reducing the risk of stunting in children living in Senegal with the largest differences being between the poorest and middle to wealthiest families.

*Maternal Education and Empowerment*

Parental Education

Maternal education plays an important role in child nutrition both as an indicator of socioeconomic status (SES) but also due to educated women being more knowledgeable of nutritious diets, being more likely to use antenatal care services, and being less likely to have adolescent pregnancies. In survey datasets, child stunting prevalence is consistently higher among mothers with no education as compared to those with primary and or those with secondary or higher levels of education. While controlling for other factors that may affect SES such as father’s education, geography, access to piped water and toilet attenuate, maternal education was found to be significantly associated with HAZ scores of children in a number of countries including Senegal (24). Along with mothers’ education being associated with HAZ and stunting, fathers’ education also plays a positive role in child HAZ (b=0.03, SE: 0.01, p<0.01 and b=0.01, SE: 0.004, p<0.05, respectively), stunting (b=-0.005, SE: 0.002, p<0.01 and b=-0.004, SE: 0.001, p<0.01) and severe stunting (b=-0.002, SE:0.001, p<0.10, b=-0.002, p<0.01) in Senegal (11). Mothers’ education was found to predict 6% of the change in child HAZ and 3% of the risk of stunting while fathers’ education predicted 1% and 2%, respectively, from 1993 to 2014 (11). A second national Senegalese study had similar findings where fathers’ education in single years (b=0.01, SE:0.005, p<0.01) and mother's education in single years (b=0.03, SE:0.008, p<0.01) was associated with child HAZ while adjusting for confounders (22). Together, parental schooling predicted 8.8% of the 66% of change in child HAZ explained from 1993 to 2011 (22). A study including the regions of Fatick, Kaolack and Kolda found mothers having primary (b=0.18, SE: 0.06, p<0.01) or secondary education (b=0.34, SE: 0.14, p<0.05) as compared to no education having improved child HAZ (18). Only secondary education for fathers as compared to no education improved child HAZ (b=0.28, SE: 0.08, p<0.01) (18). In the urban area of Pikine, maternal education, professional status, and length of residence to Pikine were found to not be associated with stunting among children up to 36 months of age (n=751)(17). Overall, the results of these studies suggest that parental education is protective against child stunting.

Subnational studies of rural areas found no evidence of the importance of maternal education on reducing the risk of child chronic malnutrition. In the rural area of Niakhar, no association between maternal education and HAZ was found but maternal education did positively influence children’s educational attainment (20). In the Podor Health District, a cross-sectional study found no association between the risk of stunting and maternal education (literate vs. illiterate) (OR= 0.80, 95% CI: 0.47,1.37, p=0.4) and maternal employment (OR= 0.94, 95% CI: 0.45,1.97, p=0.9) among children between 6 and 23 months of age (n=374) (15). Findings were constant among 12 to 42 month old children living in a rural area of Senegal of maternal schooling (none vs. any) not influencing child HAZ or linear growth (23). These findings were further supported by a study including 110 rural villages where mothers having some schooling as compared to none was not associated with child LAZ (b=-0.04, SE: 0.08) (16). The findings of these subnational studies imply that maternal education may not be as protective against chronic child malnutrition in rural areas as compared to urban areas. Although educated women are more likely to use health services, in rural areas poor access to healthcare may lead to more equal usage of healthcare services by both educated and uneducated women.

Women Empowerment

Women empowerment is thought to be important to child health as women are the primary care providers and have a level of control over the type of healthcare their children receive. Senegalese woman make the decisions over what foods are cooked, while men have greater decision-power over their children and wife’s health, children’s education, daily expenditure, and visits to relatives (25). On average, Senegalese women’s relative decision making power is among one of the lowest in the world (26). Beyond proxy measures of women’s bargaining power, no studies examining women’s empowerment as a predictor of stunting in their children were found in Senegal. However, this association is plausible as a study in three rural communities of the Saint-Louis region of Senegal found woman’s bargaining power to increase their child’s nutritional status, measured via circumference of the left upper arm (25). Bargaining power was assessed on a survey conducted in 2009 including questions regarding who makes decisions concerning the wife’s health, the children’s health, children’s schooling, daily and large expenditures, the food cooked, visits to the wife’s relatives, and whether the wife can go without the permission of the husband (25). Younger mothers especially in relation to the head of the household may indicate their social status and power to make decisions in a household. A study including three regions (Fatick, Kaolack and Kolda) of Senegal used the age difference between a mother and the head of household (greater than or less than 20 years) as an indicator for social status, and found it was not associated with child HAZ (b=-0.06, SE:0.04). Furthermore, this study found no evidence that the presence of a NGO (b=0.17, SE: 0.09, p<0.10), health post (b=-0.14, SE: 0.09, p<0.10), having both a NGO and a teenage pregnancy, or having both a health post and a teenage pregnancy (b=0.07, SE: 0.11) modified the effect of mothers’ social status on child HAZ (18). Additionally, polygamous relationships may determine a woman’s social standing in the household. A study in Pikine examined weather polygamous marriage was associated with HAZ among children up to the age of 36 months (n=751) in 2009-2010 but found no association (17). Although, proxy measures of women’s bargaining power such as their social status in the household including their age difference with respect to the head of the household and weather they are in a polygamous relationship were not found to be associated with child stunting, more research is needed using other measures of women’s empowerment in Senegal.

**Basic causes (Intermediate I)**

*Programs*

Community Nutrition Project (PNC)

The Community Nutrition Project provides three nutritional interventions: food supplementation, nutrition education, and growth monitoring and promotion. It was evaluated via the administration of two surveys: one in 1995 prior to program implementation for children between the ages of 6 and 35 months and their mothers (n=1807) and the second 18 months post program implementation (n=1689)(27). The neighbourhood of Keur Cheikh Ibra was examined as the intervention area while Champ de Course and Keur Yelli were less poor control neighbourhoods all located in Diourbel. The reduction in the prevalence of stunting and severe stunting between the baseline and the second survey did not significantly differ between the control and intervention zones (PR= 1.22, p=0.21 and PR=0.92, p=0.79) for children between the ages of 6 to 35 months. Results were consistent across age groups. The reduction in the prevalence of stunting did not differ between the intervention and control groups (PR=0.95, p=0.90) and among children who had ever been exposed to PNC interventions vs those who had never been exposed (PR=1.19, p=0.63) for children between the ages of 24 to 35 months (15.8%, n=385 versus 10.1%, n=148, p=0.11) (27). The results of this study suggests that in the first 18 months of implementation, the PNC was not effective in reducing child stunting in the one neighbourhood evaluated. However, it is possible that results may vary across villages and the PNC may have had a greater impact after five years of implementation.

The Nutrition Enhancement Program (PRN)

The Nutrition Enhancement Program (PRN), beginning in 2002, was the flagship program on the CLM in Senegal. From 2004-2006, there was an assessment of the proportion of underweight children living in villages where services were provided to determine the effectiveness of this large-scale community growth promotion program (28). Service providers targeted rural areas and included NGO’s, mobilised community health workers and nutrition workers. The services provided included: growth monitoring and counselling to all mothers of young children in selected communities, encouraging pregnant women and caregivers to seek preventive health care such as antenatal and postnatal care and coordinating with health personnel for delivery of essential health services, such as vaccination, deworming (500mg mebendazole every 6 months for children between 12 and 59 months) and micronutrient supplementation on a biannual basis. Vitamin A dosages were 100 000 IU for children between 6 and 11 months and 200 000 IU for children between 12 and 59 months. In 2004, when service provision of the PRN began, children in villages receiving PRN services were more likely to be underweight, receive vitamin A in last six months, and take oral rehydration salts to treat diarrhea as compared to children in non-intervention villages. By 2006, children in intervention villages were less likely to be introduced to liquids early, more likely to receive vitamin A in the last six months, be dewormed in last 6 months, be in a household with bednets and take oral rehydration salts for diarrhea. Mothers in PRN service coverage villages were more likely to take iron supplements and malaria pills as compared to mothers in nonintervention villages in 2006. Children living in intervention villages were less likely to be underweight with the risk of being underweight increasing between the ages of 6 and 24 months. Mothers having primary schooling and fathers having secondary schooling decreased the risk of children being underweight (28). The first phase of the PRN seems to have been effective in providing essential health services to mothers and children, however its effectiveness in reducing stunting was not evaluated in this study.

Small-Scale Programs

The MVP

A multicounty initiative called the MVP is a multisector rural-development project implemented in 9 sub-Saharan countries over a 10-year period (29). It provides a concurrent package of interventions in agriculture, health, education, and infrastructure sectors. The interventions were initially delivered to approximately 1000 households and 300 households were sampled from for this study from 2005/2006 to 2008/2009. The prevalence of childhood stunting after three years of project initiation was evaluated. In Senegal, mean HAZ (p=0.66) and stunting (p=0.16) did not significantly improve from baseline to the 3-year follow-up (29).

Counterpart International’s Maternal and Child Health Nutrition Program

Food aid programs provide food for children in times of emergencies in Senegal (30). A randomized trial was conducted using children who were eligible for Counterpart International’s Maternal and Child Health Nutrition program funded by the USAID Food-for-Peace program in Podor, Saint Louis (30). Children were assigned either potato granule-soy protein concentrate or corn-soy blend as part of a monthly ration and were followed for 4 months from late 2008 to early 2009. The study included 345 Senegelese children between the ages of 18 and 57 months of which 178 were assigned corn-soy blend supplemental rations. The results of the trial found that while mean HAZ increased in both groups there was no significant difference in mean HAZ between the two supplement types. Thus, potato granule-soy protein is an excellent alterative to corn-soy blend as it is more easily digested due to having a lower fiber content and it requires less fuel to prepare. Targeting undernourished children with food-aid programs over a period of four months seems to be beneficial to child growth (30).

Terre des Hommes’s Health Program

In the Podor Health District, there are health programs funded by Terre des hommes (TDH) (15). A study examined the relationship between stunting and intervention zones and found no difference in the risk of stunting for children between the ages of 6 and 23 months (n=374) (OR= 0.83, 95% CI: 0.49,1.41, p=0.5) (15).

**Underlying causes**

*Sanitation and Access to Safe Water*

Access to clean water and sanitation facilities are important predictors of child health and nutrition. Only 68.3% of the population of Senegal has access to a public tap or piped water near their household although it has improved by 26.2% since 1992. The number of sanitation facilities have doubled from 1992 to 2017 but still only half of the population have sanitation facilities (50.6%). Consequently, engagement in open defecation has gradually reduced from 38.9% in 1992 to 14.9% in 2017 (31,32). Two national studies examined these WASH indicators as risk factors of stunting for children under-5 in Senegal. Kampman et al. found that from 1993-2014, piped water and open defecation were not associated with HAZ, stunting, or severe stunting (b=-0.23, SE: 0.04, b=-0.01, SE:0.01, b=-0.01, SE: 0.01 and b=0.02, SE:0.06, b=0.02, SE: 0.02, b=0.02, SE:0.01, respectively) (11). Headey et al. had consistent findings of no association between the proportion of villages with households with toilets (b=-0.10, SE:0.07) or households with piped drinking water (b=0.06, SE: 0.05) and child HAZ (22). However, the authors conducted a decomposition analysis to determine the percent contribution of these factors to change in HAZ over time and found 6% of the 66% of predicted change in HAZ from 1993 to 2011 was attributed to improved piped water access (22). A older subnational study had opposing results when comparing water sources in 2001(15). In the Podor Health District using the river or pond as a source of drinking water decreased the odds of stunting (OR=0.33, 95% CI:0.15,0.72, p=0.005) among 6 to 23 month old infants (n=374). This may be due to families who use tap water being more likely to live in crowded semi-urban conditions leading to higher exposer to poor socio-economic and dietary conditions, or to non-diarrheal infectious diseases leading to poor growth (15). Although, no studies found that having access to sanitation facilities or to clean water were associated with child HAZ, piped water access was found to reduce the stunting rate in the under-5 population over time.

*Access to Health Services*

Antenatal visits provide an opportunity for mothers to ask questions to their healthcare providers that they might have as well as present an opportunity for them to receive interventions that may improve their or their baby’s health. The WHO recommends four or more antenatal visits for optimal health outcomes. In 2017, while nearly all Senegalese women receive at least one antenatal visit (96.3%), only half received four or more (57%) (32). National studies have shown the importance of antenatal visits and giving birth in health facilities as preventative factors of chronic malnutrition in children under-5. Kampman et al. found that having 4+ antenatal visits (ANC) increased HAZ scores (b=0.13, p<0.01) and reduced the risk of stunting (=-0.03, p<0.01) and severe stunting (b=-0.01, p<0.01) while adjusting for confounders (11). Children being born in a medical facility also increased HAZ (b=0.10, p<0.01) and reduced the risk of stunting (b=-0.04, p<0.01), and severe stunting (b=-0.02, p<0.01). Together, these healthcare indicators predicted 14% of the change in mean HAZ and 15% of the change in the prevalence of stunting from 1993 to 2014 (11). A second national study supported these finding of 4 of more ANC visits (b=0.15, SE:0.04, p<0.01) and children being born in a medical facility (b=0.16, SE:0.04, p<0.01) being positively associated with HAZ for children under 5 (22). Combined, these indicators were found to be the most important factors and contributed to half of the explained change in HAZ in the decomposition analysis from 1993 to 2011 (33.5% out of 66%)(22). However, a subnational study of a rural area found no evidence of prenatal consultation being protective against child stunting (OR= 0.59, 95% CI: 0.33,1.06, p=0.08) among children between the ages of 6 and 23 months (n=374)(15). Moreover, a subnational study in Pikine found the place of birth (hospital, health center, clinic, or home) of children up to 36 months of age was not associated with the risk of stunting (n=751) (17). Differences in these findings may be attributed to the smaller sample size of the subnational studies as well as children between the ages of 36 to 59 months not being included.

A study conducted in the regions of Fatick, Kaolack and Kolda examined weather having a health post, a NGO or an NGO that provides services that may improve nutrition such as child sanitation, nutrition, and alphabetization is related to child HAZ (18). The presence of a health post (b=0.17, SE: 0.06, p<0.01) improved child HAZ while the presence of a NGO (b=-0.05, SE: 0.06) or a NGO with nutritional activities (b=-0.005, SE: 0.06) did not have an impact of child HAZ while adjusting for confounders. These findings may be a result of a lack of information of how long the NGOs have been present in the villages and who actually uses the health services they provide (18).

*Inadequate Care*

Measles immunizations have fluctuated throughout the years but dramatically improved from 51% of children being immunized in 1990 to 90% in 2017 (33). In 2009, Dakar experienced a measles outbreak due to failure of the immunization program (34). Of the total of 767 cases reported, 67.4% were children under the age of five (34). Although no studies were found that examined the association between measles and stunting, a randomized control trial was conducted comparing vaccines for measles in 1990. The trial compared child mortality between children who received the Edmonston-Zagreb high titre vaccine (EZ-HT) at 5 months of age as compared to those who received the standard Schwarz vaccine at 10 months to prevent measles (35). The results of the trial found that although the EZ-HT vaccine was effective in preventing measles, it increased the risk of child mortality and so was not recommended to be used. A follow-up study found that although children who had received the EZ-HT vaccine were not at a greater risk of stunting, they were at a 2.85 times greater risk of wasting and a 1.36 times greater risk of being underweight as compared to the children who had received the Schwarz vaccine (95% CI: 1.34, 6.06, p=0.007 and 95% CI: 1.10, 1.68, p=0.005, respectively) (35).

*Food Security*

Senegal is a highly food insecure country ranking 66 out of 119 countries in the 2018 Global Hunger Index (36). The southern and central regions including Ziguinchor, Kolda, and Kédougou are the most food insecure(37). Thus, regional variations exist with regards to the prevalence of stunting with the southern regions experiencing the highest levels (38,39). Food insecurity in the south is likely attributed to the long-term Casamance conflict which began in 1982. The conflict led to declines in agricultural production, destruction of infrastructure and livelihood assets and increased poverty in Ziguinchor (37). The central regions of the country, referred to as the “groundnut basin”, are largely dependent on the agricultural economy and are prone to climate shocks. Climate change is one of leading contributors of food insecurity in Senegal where years with lower rainfall result in lower crop production (37). Efforts have been made as early as the 90s to reduce the burden of droughts and floods in areas near the Senegal river. An irrigation system was built in 1989 to gain control over water for agriculture and human requirements. Two dams were built: an antisalinization dam in the Diama delta and a reservoir at Manantali on the Bafing tributary of the Senegal River in Mali (40). The irrigation system increased the production of foods such as rice and significantly decreased the amount of food purchased, food produced by flood recession agriculture and increased food produced by modern irrigated agriculture from 1990 to 1991 (41). A study conducted in this area between February 1990 and July 1991 found the prevalence of stunting decreased slightly the first two years and then increased again after 1993 and did not greatly change overall from 1990 to 1995 (23.5% vs. 21.5%) (40).

*Infant and Young Child Feeding*

Duration of Breastfeeding

Exclusively breastfeeding children for six months and continued breastfeeding up to two years is the recommendation by WHO for optimal child nutritional health. In Senegal, the prevalence of exclusive breastfeeding has drastically improved from the early 90s to present but remains alarmingly low [5% (1993) to 36% (2016)](42). Qualitative interviews with Senegalese mothers of the Niakhar area in 1997 (n=485 mother-child pairs) revealed that the average duration of breastfeeding was approximately 2 years and reasons for weaning earlier included that the child “ate well from the family plate” (60%), the child was “tall and strong” (46%), and maternal pregnancy (35%) (43). Reasons for weaning later than 2 years of age included that the child was weak (33%), there was a food shortage (25%), the child had an illness (24%), and refusal of family food (14%). Children weaned both prior to or past the age of 2 (at the age of 24-33 months) for being “small and weak” had a lower mean HAZ (p<0.0001) but those weaned past the age of 2 for non-nutrition related reasons had a similar mean HAZ as compared to their earlier weaned counterparts. These children who were weaned past the age of two were found to have a greater prevalence of moderate and severe stunting due to being “small and weak” and non-nutrition related reasons as compared to their counterparts [31.6% and 23.7% (p<0.0001), respectively] (43). A study followed these children (n=443) from 2 months to nearly 3 years of age to examine the effect of breastfeeding duration on child HAZ while accounting for HAZ at 2 months(44). They found duration of breastfeeding (<22 months, 22-23.9 months, 24-25.9, over 26 months) was not a significant predictor of HAZ at 3 years when controlling for HAZ during infancy and sociodemographic characteristics (p=0.58). The authors also explored the relationship between linear growth in height and duration of breastfeeding. Children who were breastfed for two years grew faster in length compared to their counterparts. However, of the children breastfed after three years, only those with poor housing grew faster as compared to their earlier weaned counterparts (b=0.57, SE:0.29, p<0.05). Overall, the longer children were breastfed, the faster they grew up to three years of age. Differences in results between these two studies may be attributed to the previous study not controlling for HAZ during infancy which is necessary when accessing causation. Moreover, the previous study did not control for potential confounding variables such as sex, maternal age, education, housing which are associated with child HAZ and breastfeeding duration.

Early Introduction of Complementary Feeding

A number of studies in rural areas supported complementary feeding to commence no earlier than six months of age. From 1992-94 in Niakhar, early complementary feeding was found to be strongly associated with lower length-for-age (p=0.02) for 2-3 month old infants as compared to those predominantly breastfed (45). Additionally, infants who received complementary feeding at 6-7 months had similar linear growth to those predominantly breastfed. The results of this study provide no evidence for complementary feeding prior to six months being beneficial to child growth (45). A trial was conducted in the rural area of the peanut basin in Central Senegal (150 km from Dakar) in 1993 examining the effect of supplements for 4 month olds on linear growth faltering (46). Children were randomly allocated to a cereal-based precooked porridge to be eaten twice daily for 3 months or the control group (n=110). The results found that supplemented infants grew faster between 4 to 5 months as compared to control infants (p<0.01). The supplemented infants grew on average 0.48 cm in height more than the non-supplemented infants between the ages of 4 to 7 months old (p=0.02). However, this change in effect may be attributed to the greater growth potential of the supplemented group as these infants started off on average 0.58 cm shorter then control group infants leading to both groups being of equal height at 7 months (46). A study conducted in the rural Fatick region assessed mothers’ 24 hour recall of dietary patterns of 2 to 10 month old infants (n=1174) in 1991 (47). They found that LAZ was lower for 2 to 3.9 month infants who were supplemented as compared to those who were exclusively breastfed (LAZ: -0.81 vs. -0.29, p<0.01). Mothers may supplement breast milk with millet gruel or the family diet. Supplementing with millet gruel was found to have a negative effect on length-for-age (LAZ) for infants between 2 to 3.9 months (p=0.001) but not those above 4 months and supplementing with the family diet had no effect on LAZ. Mothers are likely to supplement breast milk for their infants that they perceive to have low growth or inadequate breast-milk intake, however this in turn has a negative effect on the infant’s growth. Supplementing with millet gruel may cause diarrhea due to the high bacteria contamination leading to impeding growth. Indeed, this study found that infants who were given millet gruel prior to 6 months of age had a lower HAZ as compared to those who received it after 6 months (p<0.05) (47). A study from Niakhar found mothers to prolong breastfeeding by a median of 2.3 months for their stunted infants (n=4515) (p<0.0001). At 9-10 months, the prevalence of stunting among infants breastfeed for over 30 months was 4 times greater as compared to those breastfed under 18 months (48). The results of the 1989 DHS survey found breastfeeding infants between the ages of 9 and 36 months to be associated with a shorter stature (length/height) (b=-5.37, SE: 1.38, p<0.05)(49). Furthermore, there was a significant interaction between age and breastfeeding (b=0.17, SE:0.06, p<0.05). One possible explanation for this outcome may be that children weaned later may develop a preference for breastmilk and may continue breastfeeding for longer and not have a well-balanced nutritious diet (49). A subnational study in Dakar examined the benefits of exclusively breastfeeding for 6 months with regards to energy intake (50). They found infants (n=59) who are exclusively breastfed for 6 months have a higher energy intake from breastmilk (derived from the energy content of breast milk and breast milk intake) than partially breastfed infants (p<0.01) but HAZ remained similar in both groups (50). In summary, all studies support beginning complementary feeding after 6 months of age for optimal child growth.

Sex Differences in Complementary Feeding Practices

A prospective follow-up study of infants born between 1990 to 1996 who are up to the age of 39 months was conducted in Niakhar (51). They found sex differences tend to exist with regards to early complementary feeding as 2 to 3 month old boys were found to be more likely to have been fed complementary food every day during the past week (15.8% compared with 11.2% for girls; p= 0.005) and to have eaten more than 2 meals in the past 24 hours (13.4% compared with 8.2% for girls; p< 0.001). In 9-10 month old infants, girls were more likely to have eaten complementary foods every day in the past week (65.1% compared to 81.2% for boys, p=0.04). Consequently, mean HAZ differed between sexes when comparing frequency of meals (0, 1, 2, 3 or more meals in the past 24 hours) with boys having a lower mean HAZ above the age of 6 months (51). This study provides one possible explanation for sex differences in HAZ seen in Senegal as boys are introduced to complementary foods at an earlier age than recommended whereas girls eat complementary foods more frequently at an appropriate age.

Early Introduction of Water

Along with complementary feeding, the early introduction of water is common in African countries (15). In the Podor Health District, a cross-sectional study assessed the effects of complementary feeding and the early introduction of water on the HAZ of children between 6 and 23 months (n=374). They found no association between the introduction of complementary feeding before 6 months of age (OR=0.79, 95% CI: 0.46, 1.35, p= 0.4) or the introduction of water before 3 months (OR= 0.68, 95% CI: 0.34, 1.36, p=0.3) with the risk of stunting. Moreover, no associations were found between child stunting and colostrum as first food (OR=0.75, 95% CI: 0.41,1.37, p=0.3), time of first breastmilk administration (before 30 minutes, 30 minutes-hour, or after an hour) (OR= 1.89, 95%CI: 0.73,4.85, p=0.2 and OR=0.94, 95% CI:0.54,1.66, p=0.8, respectively) and number of meals during the day (OR=0.95, 95%CI: 0.72,1.24, p=0.7) (15). In Senegal, it is a common religious practise to provide children with water as the first drink after birth. Thus, more research is needed to uncover the potential detrimental effects of water on child growth.

**Immediate causes (Proximal)**

*Disease*

Malaria

Senegal is part of the malaria belt and thus malaria control has been important to reduce child mortality in Senegal. The National Malarial Control program helped significantly reduced the prevalence of malaria from 2008 to 2010 however, there was an increase in the prevalence of stunting from 16% to 26% from the pre-intervention period to the post intervention period (52). In 2002, there was a randomized, double-blind, placebo-controlled intervention study of seasonal intermittent preventive treatment (IPT)5 of malaria conducted in a cohort of 2 to 59 month old children living in a rural area of Senegal. The results of this study found that the mean HAZ z-scores between trial (mean=-1.17, SD: 0.93) and control children (mean: -1.24, SD: 1.00; p = 0.25) were not significantly different (53). Moreover, only 36 to 47 month old malaria trial children had a lower prevalence of stunting as compared to the control non-trial children (19.4 % vs. 28.7%; p<0.05). No difference in height increments between the intervention group as compared to the control group were detected but seasonal variation in mean HAZ scores in at least one of these groups was present (54). The association between malaria and chronic child malnutrition in Senegal is unknown as no studies were found that examined this relationship.

Anemia

The prevalence of anemia remains alarmingly high and has only gradually decreased from 1990 (83.0%) to 2016 (67.9%) among children under the age of 5. A subnational study in Bonconto (500km from Dakar) examined risk factors for anemia (55). They found malaria and stunting placed children at a 5.2 times greater odds (95% CI: 1.1,28.4, p=0.04) and a 3.37 times greater odds (CI: 1.93, 5.99, p=0.001), respectively, of anemia. Stunted under-5 children as compared to non-stunted children were at a 3.1 (95% CI: 1.4, 6.8, p=0.004) times greater odds of anemia whereas stunted children between the ages of 5 to 10 years of age were at a 3.6 times greater odds of anemia (95% CI: 1.6,8.2, p=0.002) (55). A second study by Tine et al. had consistent findings with both malaria and stunting being risk factors for anemia (adjusted OR: 2, 95% CI: 1.2, 3.1, p=0.004 and adjusted OR: 6.3, 95% CI: 1.5, 53.5, p=0.03, respectively) (56). However, they found that the relationship between stunting and anemia is bidirectional. Moderate (adjusted OR: 1.7, 95% CI: 1.1, 2.8, p=0.02) and severe anemia (adjusted OR: 3.5 (95% CI: 1.9, 6.4), p<0.0001) were associated with stunting in children under-5 from Lamarame, a rural community 200 km from Dakar (56). Overall, subnational studies have found that anemia and stunting happen to occur alongside each other among under-5 children in Senegal.

Parasites

It is common for children living in rural areas of Senegal to be affected by parasitosis with the type of parasite varying between villages. In three rural villages of the Khombole district, intestinal parasitosis was associated with HAZ with 39.2% of stunted children (HAZ<-2) having intestinal parasites, while only 27% of non-stunted children had intestinal parasites (*X*^2^=4.35, p=0.03) (n=400) (57). In a second study of the same sample in 1997, stunting was associated with intestinal parasitosis, anemia (*X*^2^=10.8, p=0.001) and geophagy (*X*^2^=6.17, p=0.009) among children under 5 (58). However, one study found no evidence that the malaria parasite nor a number of intestinal parasites were associated with stunting in children under-5 from Lamarame, a rural community 200 km from Dakar (56). While evidence is mixed regarding the negative impact of intestinal parasites on child HAZ, more research is needed including studies with larger sample sizes.

Vitamin A Deficiency

Vitamin A is necessary for growth and deficiencies may lead to negative child outcomes including stunting, blindness, and mortality (59,60). Few subnational studies examined the association between vitamin A deficiency and stunting in Senegal. In Linguere, a rural area of Senegal, no association between retional status, an indicator used for vitamin A deficiency, and stunting or wasting was found (61). In the Podor Health District, a cross-sectional study found consistent findings of no association between the risk of stunting and vitamin A supplement use (OR= 0.79, 95% CI: 0.45,1.36, p=0.4) or the use of mosquito nets (OR= 1.44, 95% CI: 0.49,4.30, p=0.5), recent illness (OR= 0.92, 95% CI: 0.53,1.60, p=0.8) and recent diarrhoea (OR= 0.59, 95% CI: 0.27,1.31, p=0.2) among children between 6 and 23 months (n=374) (15). Propective studies are needed to examine whether vitamin A deficiency leads to stunting in Sengelese children.

HIV

The number of children up to the age of 14 living with HIV has increased 10-fold from 500 cases in 1990 to 5000 cases in 2017 (62). Treatment for HIV including antiretroviral therapy (ART) slows its progression and reduces the risk of negative outcomes such as stunting and wasting which are common among HIV-infected youth. One study found that at the time of ART initiation, children with HIV were at a 2.12 times greater risk of wasting (95% CI: 1.42, 3.14) and 1.85 times more likely of being underweight (95% CI: 1.27, 2.69) but not stunted (adjusted OR: 1.22, 95% CI: 0.83 to 1.77). However, after 24 months of ART treatment, children with HIV were 2.16 times more likely to be wasted (95% CI: 1.58, 2.97), 1.83 times more likely to be stunted (95% CI: 1.31, 2.06) and 1.28 times more likely to be underweight (95% CI: 0.93, 1.78) (63). A second study found age of enrollment into the study [2-5 vs. 5-10 and 10-16 vs. 5-10, 9.7 (3.0–30.9) <0.001 and 5.6 (2.1–14.9) <0.001, respectively] and being stunted at the time of antiretroviral treatment (ART) initiation [adjusted OR: 11.6 (5.4–25.0) <0.001] to be risk factors of stunting among HIV infected youth and did not find the mother being alive and ART treatment initiation before 5 years of age to be predictors (64). The findings of these two studies are suggestive of HIV being a risk factor for stunting, however further research is needed to detangle this relationship.

*Inadequate Dietary Intake*

A well-balance nutritious diet is necessary for growth and the prevention of stunting. A 2009 prospective cohort study in the Sine region was conducted that examined 2 visits to assess food consumption patterns of 6 to 36 month Senegalese infants (n=1060) (65). The results showed that dietary diversity and food variety were positively associated with HAZ in 6 to 24 month old infants. The overall infant and young child feeding index score (compromised of dietary diversity, meal frequency, breastfeeding, bottle-feeding, and frequency of food consumption) was positively associated with HAZ for 6-12 month (p<0.001) and 18 to 24 month old infants (p=0.02). However, breastfeeding was negatively associated with HAZ for infants between 12 to 30 months. Linear growth among infants between the ages of 18 and 24 months was positively associated with the infant and young child feeding index along with meal frequency but not with dietary diversity, food variety or breastfeeding for infants of all ages(65). In a rural area of Senegal, the young child feeding index was not found to be associated with either height-for-age or with linear growth, measured 7 months before (n=500) among 12 to 42 month old infants (23). They also found continuing breast-feeding was negatively associated with height-for-age (p<0.05) but positively associated with linear growth (p< 0.01). Frequent consumption of fruit, milk, fish/meat and cereal were not associated with HAZ or linear growth, however vegetable consumption was positively associated with HAZ p<0.05) (23). A cross-sectional study in a village in Senegal (Potou) examined whether animal source consumption and owning livestock reduced the risk of chronic malnutrition in children (n=293 children) (66). Consumption of beef and eggs was found to be associated with better HAZ scores while other animal source products including milk/milk products, sheep/goat meat, poultry meat and owning livestock were not (66). The inconsistencies in findings among these studies may be attributed to differences in the measurement tools used to access dietary intake patterns and differences in the samples, i.e. different villages, age groups, and sample sizes.

*Maternal Characteristics*

Parity

The number of children a mother has may affect their children’s growth due to the division of food amongst children. Moreover, having multiple children over a short period of time may lead to the mother’s body being unable to provide adequate nutrients to the younger siblings. A national study using 1993 to 2011 DHS data found no association between the total number of children ever born (b=-0.007, SE:0.01) and child HAZ while controlling for confounders (22). Another national study supported this finding of the total number of children a family not being associated with HAZ scores and the risk of being stunted or severely stunted among children under-5 (11). Preceding birth interval however, was found be associated with HAZ (b=0.04, SE: 0.008, p<0.01), stunting (b=0.01, SE: 0.002, p<0.01) and severe stunting (b=-0.01, SE: 0.002, p<0.01) (11). A study including three regions (Fatick, Kaolack and Kolda) of Senegal had a similar finding of being a twin being associated with lower HAZ (b=-0.69, SE: 0.17, p<0.01) (n=4047) (18). While no studies found evidence of the number of children a mother increasing the children’s risk of stunting, short birth interval does seem to be an important risk factor.

Maternal Height and Age

Maternal height and a mother’s age when she gives birth may affect their child’s growth. A national study found mother's height (b=0.04, SE:0.003, p<0.01) was positively associated with child HAZ while controlling for confounders (22). In their decomposition analysis, maternal height predicted 8.1% of the total explainable (66%) change in HAZ from 1993 to 2011(22). A subnational study from a rural area of Senegal found maternal height, number of children in a household under-5 and maternal BMI to not predict HAZ or linear growth among 12 to 42 month old infants (n=500) (23). A second subnational study had consistent findings of maternal height not being associated with child HAZ in Niakhar, a rural area in Senegal (p<0.001)(20). Moreover, in Pikine maternal height and age were not associated with stunting among children up to 36 months of age (n=751)(17). A fourth subnational study in the Podor Health District also found no association between the risk of stunting and maternal age (>20 years) (OR: 1.48, 95% CI: 0.55,3.94, p=0.4) and maternal ethnicity (Halpular) (OR: 1.47, 95% CI: 0.69,3.15, p=0.3) among children between 6 and 23 months (n=374) (15). A study including three regions (Fatick, Kaolack and Kolda) of Senegal examined the effect that being a teenage mother, under the age of 20, has on child HAZ. The study found that being a teenage mother decreased child HAZ for children between the ages of 0 to 35 months (b=-1.9, SE: 0.05, p<0.01). The effect that being a teenage mother had on child has was not modified by the mother’s social status in the household defined as having a large age gap (i.e. more than a 20-year age gap from head of household) (b=-0.11, SE: 0.13). Furthermore, this study found no evidence that the presence of a NGO (b=0.08, SE: 0.10), health post (b=0.10, SE: 0.11), having both a NGO and a large age gap (b=0.19, SE: 0.10, p<0.10), or having both a health post and a large age gap (b=0.07, SE: 0.11) modified the effect of teenage preganancy on child HAZ (18). These findings imply that being a teenage mother has a strong negative impact on child HAZ and cannot be significantly decreased by the presense of NGOs or health posts. Although most of subnational studies did not find evidence of the importance of maternal height, age and other maternal characteristics, the one national study did find that maternal height played a large role in reducing the risk of child stunting.

*Child Characteristics*

Sex

The majority of studies are in agreement of boys being at a greater risk of being stunted. In a national study using 1993-2014 DHS data, boys were found be at a higher risk of having a lower HAZ (b=-0.15, p<0.01), being stunted (b=0.05, p<0.01), and being severely stunted (b=0.02, p<0.01) as compared to girls (11). Results of the 1989 DHS survey also showed sex (b=1.23, SE: 0.34, p<0.05) differences with regards to stature (length/height) among infants between the ages of 9 and 36 months (49). In Niakhar, a prospective cohort study of infants born between 1990 to 1996 was conducted to examine sex differences with regards to the prevalence of stunting(51). They found that the prevalence of stunting was higher for boys as compared to girls for infants between the ages of 2 and 39 months (p<0.001). The risk of being stunted more than once during infancy was also higher among boys (24.5% as compared to 19.4% among girls, p<0.001). As such, mean HAZ significantly differed between sexes with the exception of infants between the ages of 12 to 17 months. HAD (height-for-age difference) defined as the difference between the measured height and the age and sex-specific median height significantly differed between sexes for children in the following age groups: 2 to 3 (p=0.01), 4 to 5 (p<0.001), 6 to 7 (p=0.01), 24 to 29 (p<0.01), 30 to 35 months (p=0.03). HAZ and HAD significantly decreased with increasing age for both sexes but more rapidly for boys leading to greater sex differences with increasing age. These sex differences may be attributed to earlier introduction of complementary feeding among boys found in this study and or biological differences between sexes (51). A second study in Niakhar examined sex differences with regards to HAZ and stunting (20). They found that females had slightly greater HAZ but there was no difference in the prevalence of stunting between genders. In the regions of Fatick, Kaolack and Kolda (n=4047), males having a lower HAZ score as compared to females was supported (18). A study in the area of Sine had supported these finding of a lower HAZ among boys from infancy to 30 months of age (65). For infants under the age of 6 months, girls were found to have a larger increase in length/height [5.8 cm (SD:1.8) vs. 5.3 (SD: 1.6), p<0.05)] (65). A small number of subnational studies however found no association between sex and child stunting. In the Podor Health District, a 2001 cross-sectional survey found the odds of stunting was not associated with sex (OR:1.66, 95% CI: 0.98,2.83, p=0.06 ) among 6 to 23 month old infants (n=374)(15). Among the Mpal community in Saint Louis, sex was not associated with child HAZ among children under-5 in 1996 (67). A study including 110 rural communities in Senegal found males did not have a lower LAZ as compared to females (b=-0.11, SE: 0.06, p<0.10)(16). Lastly, a study in rural Senegal had consistent findings sex not being associated with HAZ or linear growth (23). Overall, all national studies and half of the subnational studies have found boys to have lower HAZ scores as compared to their female counterparts with sex difference increasing with age. More research is needed to uncover possible cultural and biologic reasons for sex differences with regards to chronic malnutrition in Senegal.

Age

The majority of studies are in agreement that the risk of stunting increases with age up to the age of 36 months. A study in Niakhar examined age differences with regards to HAZ and stunting and found that the greatest prevalence of stunting was between the ages of two and three (20). Results of the 1989 DHS survey also showed age (b=0.52, SE:0.05, p<0.05) differences with regards to stature (length/height) among infants between the ages of 9 and 36 months(49). A study in Pikine in 2009-2010 found HAZ to significantly increase with child age for children up to 36 months (n=751)(17). They also found that children born under 3 kg were more likely to be stunted (*X*^2^=5.3, p<0.05) (17). In the Podor Health District, a 2001 cross-sectional survey found the odds of stunting to increase with age (OR: 1.09, 95% CI: 1.03, 1.15, p<0.005) among 6 to 23 month old infants (n=374)(15). In the regions of Fatick, Kaolack and Kolda (n=4047), this finding of age being associated with HAZ with was supported (18). Two, small-scale subnational studies did not find increasing age to increase the risk of stunting. Among the Mpal community in Saint Louis, age not was associated with child HAZ among children under 5 in 1996 (67). A study in rural Senegal had consistent findings of age not being associated with HAZ or linear growth (23). In summary, increasing age up to 2 to 3 years is likely to increase the risk of stunting, however more national studies are needed to further examine this association.

# **Supplementary Appendix 3:** Multivariable Analyses Methods

We undertook two sets of hierarchical multivariable analyses as discussed below. Using complementary approaches, each of these analyses attempts to answer the same research question i.e. what are the main predictors of change in child linear growth in Senegal during 1992/93-2017? The linear regression based on panel datasets uses a difference-in-difference analysis framework where time*covariable interactions are used to assess factors impacting HAZ decline. This allows the analysis of multiple years of survey data and adjusts for baseline levels of covariables and varying hypothesized growth trajectories through the interaction term. The Oaxaca-Blinder decomposition is based on the same set of individual/household level data (with ecological variables). However, by design, the decomposition only uses two survey time points in a given analysis and thus “ignores” in-between survey rounds and any intermittent fluctuations in the predictors. As has been suggested in previous decomposition analyses, we operationalize child HAZ as the linear growth outcome due to its greater statistical efficiency relative to the dichotomous child stunting variable. Each of the two multivariable regression-based analysis methods pose their own strengths and limitations – however, as sensitivity analyses, study inferences should be anchored in both and congruent findings between the methods strengthen the key messages.

Linear Multivariable Regression (Difference-in-Difference Analysis)

We undertook linear multivariable regression analyses, and included all covariables and adjustment factors as fixed effects. We added interaction terms between each potential determinant and time (i.e. time*covariable interaction terms), which signify whether a change in a proposed predictor of HAZ leads to a change in HAZ over the studied time period. The four cross-sectional surveys used in this analysis were assembled into panel datasets, and difference-in-difference (DID) analyses were used. Univariate statistics were estimated using means/standard deviations and frequencies/proportions as appropriate. We used the interaction estimators in unadjusted and adjusted regression methods to estimate the DID effect. The general model specification included an interaction term between time and the various indicators. The multivariable regression models were adjusted for child age, sex and region. Effect estimates were reported with 95% confidence intervals. All statistical analyses were performed using Stata version 14.0. The complex sampling design of DHS surveys was taken into account by using the STATA's svyset function. Standard errors were estimated using the Taylor series linearization method, which incorporates sampling weight, primary sampling unit, and stratum appropriate to the DHS sample design. 

To examine the association between HAZ and various indicators, we conducted a series of step-wise linear regression models. A hierarchical modelling approach using distal, intermediate and proximal level variables was executed as suggested by Victora 1997 (68) to generate the final multivariable models. Variables within each level were selected from our general conceptual framework as defined in **Figure 2**. Step 1 was a series of bivariate regressions to determine crude associations between indicators in our conceptual framework and HAZ outcome. Step 2 was to use all candidate variables for multivariable model building (i.e. with p-value ≤0.20) irrespective of their direction to move forward for multivariable modeling. Selected variables are entered into backward stepwise elimination modeling within their respective levels and those with p-values <0.15 are retained. At each step, the crude and adjusted associations between the indicator and HAZ was analyzed for statistical significance. Multicollinearity among adjustment variables was evaluated using variance inflation factors (VIF) where VIF>3 were considered suspect for collinearity.

Oaxaca-Blinder Decomposition

We also undertook the commonly used Oaxaca-Blinder decomposition methods (69,70) to assess determinants of nutritional change over time in Senegal. These methods based on individual-level data have high statistical power and have been widely used to assess nutrition determinants in low and middle income settings (70–73).

We analyzed individual-level data from four rounds of Senegal’s DHSs: 1992/93, 2005, and 2017, as well as the 2000 MICS. Our analysis focused on the index mother-child pair from each survey round. Defined as the youngest child of the youngest mother in each household, selection of an index pair simplifies the model and interpretation, and is common practice in advanced analysis of DHS datasets. The total number of index pairs available from each survey were n=1840, n=4225, n=1355, and n= 5076 for DHS 1992/93, MICS 2000, DHS 2005, and DHS 2017, respectively. A flow chart outlining sample size breakdown during the index pair selection process is presented in the main paper. Given that the dietary needs/practices and growth trajectories of children in the first 1000 days of life vary notably from children beyond 2 years of age, it has been suggested that these two cohorts be analyzed separately to unmask true effects of environmental conditions and other factors on undernutrition. We conducted analyses for the entire under-5 year child population, the 24-59 month child population, and the 6-23-month population (74). There was an insufficient sample size in the <6-month population which prevented us from analyzing this age group. The 6-23-month population had too small a change in HAZ, thus rendering the results of analysis not meaningful. Thus, only the decomposition results for the under-5 and 24-59-month populations are presented.

We used the continuous formulation of HAZ (as opposed to categorical stunting) as the dependent outcome to strengthen statistical power of the analyses. Linear least square regression models -accounting for survey design and weights - were used to assess associations between $\boldsymbol{y}_{\boldsymbol{i,t}}$, our outcome variable measured for a child *i* at time *t*, a vector of time-varying determinants (**X**), time-invariant child age and sex control variables (**C**), and a survey round time variable (**T**) to capture any trend effects. Collectively, with the standard error term, the model is expressed in Equation 1.

$\boldsymbol{Y}_{\boldsymbol{i,t}}=\boldsymbol{\beta}\boldsymbol{X}_{i,k}+\boldsymbol{C}_{i}+\boldsymbol{T} + \varepsilon_{i,t}$ [Equation 1]

The conceptual framework and corresponding list of covariables, their data sources, and definitions used in decomposition assessment are included in Appendix 5. Applying the conceptual framework, we used a similar hierarchical modelling approach (as described for DID analysis) whereby we examined the distal, intermediate, and proximal level determinants of HAZ.

Equation 1 was applied to derive β coefficients for determinants (DHS 1992/93 – DHS 2017). To explain the relative contribution of each covariable over time to HAZ change, we used the Oaxaca- Blinder decomposition under the assumption that the β coefficients are the same across the two populations and the error term has the mean zero. Using the estimated parameters from Equation 1 and the (weighted) means of explanatory variables in the two time points, we applied Equation 2 (e.g. for years 1992/93 to 2017) to obtain the predicted change in HAZ due to the change in each determinant (75).

$$\Delta\bar{Y}_{i,t}=\beta\left( \bar{X}_{2017}-\bar{X}_{1992/93} \right)$$

The product coefficients for individual determinants were subsequently ranked to identify the relative contribution of each factor to HAZ change. Like determinants were also grouped into broader domains for interpretation. We examined variance inflation factors (VIF) to assess multicollinearity between variables whereby a VIF > 3 was considered suspect of high inter-variable correlation. For model building, a p-value <0.20 was considered statistically important and variables with p< 0.15 were retained in the final hierarchical multivariable models. All analyses were carried out in Stata version 14.0.

# **Supplementary Appendix 4:** Qualitative Data Collection and Analyses Methods

Qualitative Inquiry Process

The qualitative component of the case study aimed to understand the drivers of stunting reduction among children in Senegal through exploring the perspectives of key national stakeholders in the development and implementation of relevant policies and programs, the experiences of community health workers and mothers in the community. Specific qualitative research objectives included:

1. To explore nutrition-specific and –sensitive key events (policies/strategies/programs/guidelines) in Senegal that may have contributed to a reduction in child stunting;
2. To identify important contextual factors that have functioned as enablers/drivers and barriers to reduction of stunting in Senegal; and
3. To document community-level insight and experiences on the stunting transition in Senegal from community/volunteer health workers and mothers of young children.

The conceptual framework by Black et al., informed the development of an adapted framework (in main paper), the design of the in-depth interview and FGD guides, as well as analysis and interpretation of the qualitative data. Our qualitative data collection tools were also informed by existing literature and nutrition questionnaires; for example, the International Food Policy Research Institute’s nutrition-focused qualitative data collection toolkit was consulted and relevant tools were adapted to our research objectives as appropriate. Data was analyzed using key themes including: basic causes, underlying causes, and immediate causes of reduction in stunting and malnutrition.

Qualitative Research Design

We undertook three independent research activities to inform study objectives. At the first stage, national stakeholders were interviewed to provide insight and expertise on objectives 1 and 2. This top-down approach aimed to solicit macro-level perspectives and experiences in health and nutrition in Senegal. All the experts were interviewed in Dakar, where main administrative structures and institutions are located. To understand how individuals in the community received and implemented major nutrition-specific and –sensitive policy/program events and their experiences in the nutritional transition as a whole, we consulted childcare workers in the community (e.g. at schools, health facilities, etc.) and the mothers of these children. These latter two research activities largely informed objectives 2 and 3, but also shed light on objective 1.

*Sampling and Recruitment Strategy*

Participants were identified and selected using purposive sampling strategies (76), including snowballing sampling (77). National stakeholders were purposively selected due to their involvement in the design, implementation, monitoring or evaluation of nutrition-specific or –sensitive policies and programs (**Supplementary Appendix** **Table 6**). Key informants were asked to identify and refer the research team to other individuals with knowledge and expertise in the area of nutrition, policy, and stunting reduction. Participants were recruited by phone and a follow-up email was sent to request their participation in the study. Community health workers were purposively selected based on their experiences of working in communities for over five years, delivering primary health services, including nutrition-related services for communities. Community health workers also identified mothers of children born between 1992 to 1997 and 2012 to 2017. In the visited localities, the mothers of the different generations were identified thanks to the actors of the health system and mainly, the community health actors (health community workers).

## **Supplementary Appendix Table 1:** Inclusion Criteria

| **Type of Stakeholder** | **Inclusion Criteria** |
| --- | --- |
| National Stakeholders | - Key informants with extensive experience in and knowledge of design, implementation and evaluation of nutrition-specific and –sensitive policies and programs in Senegal. Examples include: national policymakers (e.g., Ministry of Health, Ministry of Gender, etc.), bilateral/multilateral organization (e.g., UNICEF, WHO), international/local NGOs (e.g., Hellen Keller International, Action Against Hunger). |
| Regional Stakeholders | - Paid/voluntary community stakeholders in Louga, Diourbel or Kaolack regions. Examples include: teacher, imam, chair of community health posts, traditional Chief, community health worker, midwives and local NGO representative. |
| Mothers in Communities | - Mothers of children born in 1992-1997; - Mothers of children born 2012-2017; and - Currently living in Louga, Diourbel or Kaolack regions. |

Semi-structured with regional respondents and focus group interviews with mothers were conducted in three regions: Louga, Diourbel and Kaolack. These three regions were selected based on their geographic location, ability to capture urban and rural perspectives, as well as the substantial progress made in these regions to reduce stunting among children. Focus communities were purposively selected, using convenience sampling. One rural and urban community was randomly selected within each focal region, and two FGDs were conducted in each. These sampling strategies helped to ensure that a range of diverse perspectives at national and community levels were captured. According to the 2013 population census, population estimates by region include: Diourbel (1,497,455), Kaolack (960,875) and Louga (874,193) and these regions represent the 3^rd^, 4^th^ and 6^th^ most populated regions respectively (78).


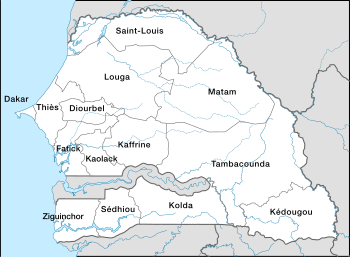


## **Supplementary Appendix Figure 3**: Regional map of Senegal displaying study sites for subnational key informant interviews and focus group discussions.

Research Methods

*In-Depth Interviews*

Firstly, 21 interviews were conducted with experts in Dakar. These experts were officials from the Malnutrition Control Cell (the main structure dedicated to the fight against malnutrition), from ministries directly or indirectly concerned by the issues of reducing malnutrition, including the Ministry of Health and experts from the specialized agencies of the Nations, international NGOs as well as professors of pediatrics at the University, active or retired. These individuals were purposively selected based on their expertise in nutrition, health and other sectors.

Secondly, 20 interviews were conducted in the three regions of Louga, Diourbel and Kaolack. These interviews targeted resource persons with several functions: nursery school teachers, health staff (doctors, nurses, midwives, community health workers, treasurers of health committees), NGO workers serving as implementation for anti-malnutrition programs, imams, and village chiefs. Interviews were conducted in both urban and rural areas. In each of the three regions, two interviews with resources persons (e.g., nurse, community health worker, kindergarten teacher, local NGO representative, etc.) were conducted in rural areas and two in urban areas.

*Focus Group Discussions*

Thirdly, 12 focus groups discussion were organized, with four groups per region. In each region, two focus groups were held with mothers who gave birth between 1992 and 1997 and 2 focus groups with younger women who gave birth between 2012 and 2017.

*Data Analysis*

Data generated during focus group discussions, and semi-structured interviews were analyzed using the UNICEF Nutrition Framework (68), Lancet Nutrition framework (79), and the adapted framework for the country case studies (**Figure 2**). These conceptual frameworks guided the qualitative analysis and interpretation of key determinants and contextual factors, as well as facilitators and barriers to nutrition-specific and –sensitive events. The qualitative analysis explored distal/basic causes (e.g., GDP, education, political context), nutrition-sensitive and -specific programs, underlying causes (e.g., inadequate feeding practices, and food insecurity, inadequate care and health services and unhealthy environment), and proximal/immediate causes (e.g., maternal characteristics, inadequate dietary intake, disease, and child characteristics). Responses from national, regional and mothers at community level were analyzed separately. Thematic analysis was conducted to explore key themes that emerged based on stunting determinants including socioeconomic status (e.g., living conditions), migration, hygiene and sanitation, and nutrition and eating behaviours.

All interviews were audio recorded with permission from research participants. Interviews were conducted in French, transcribed for analysis and translated into English. All in-depth interviews and FGDs were conducted by a team of four interviewers.

# **Supplementary Appendix 5:** Quantitative Results

## **Supplementary Appendix Figure 4A**: Spline analysis of inflection points of change in the slope of HAZ, 1992/93


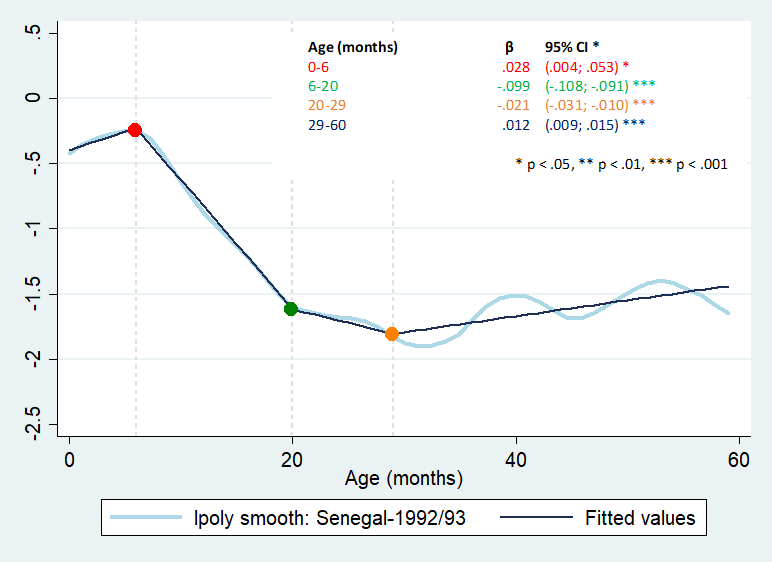


## **Supplementary Appendix Figure 4B**: Spline analysis of inflection points of change in the slope of HAZ, 2000


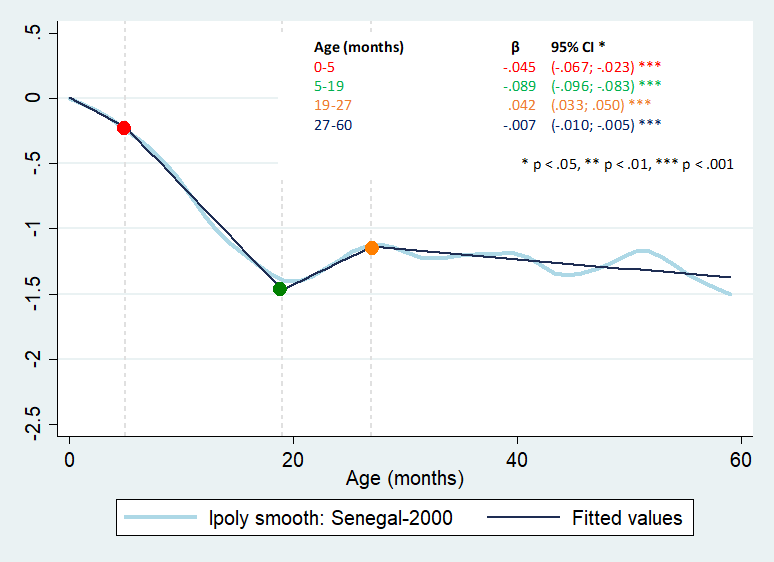


## **Supplementary Appendix Figure 4C**: Spline analysis of inflection points of change in the slope of HAZ, 2005


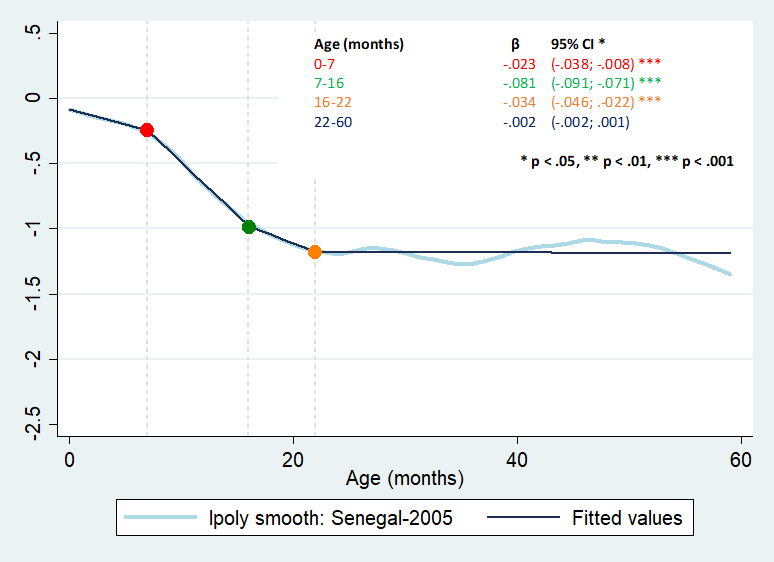


## **Supplementary Appendix Figure 4D**: Spline analysis of inflection points of change in the slope of HAZ, 2017


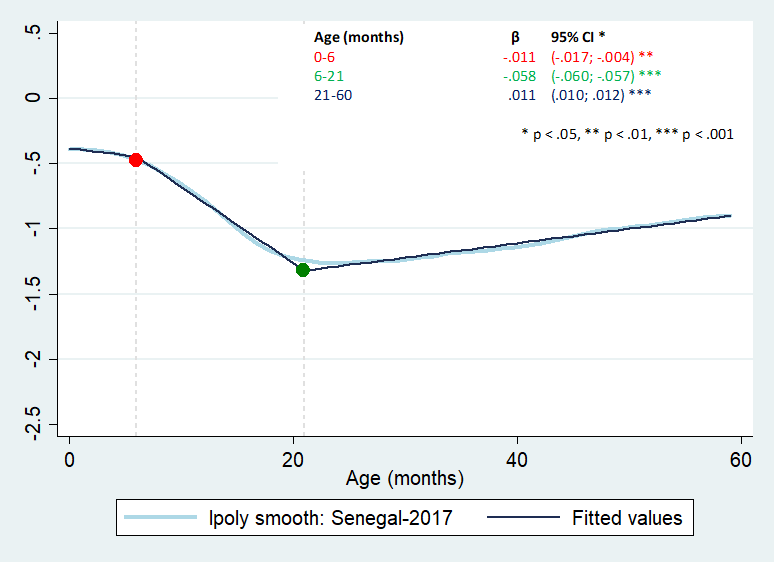


## **Supplementary Appendix Figure 5A**: 2000 stunting prevalence by region


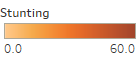

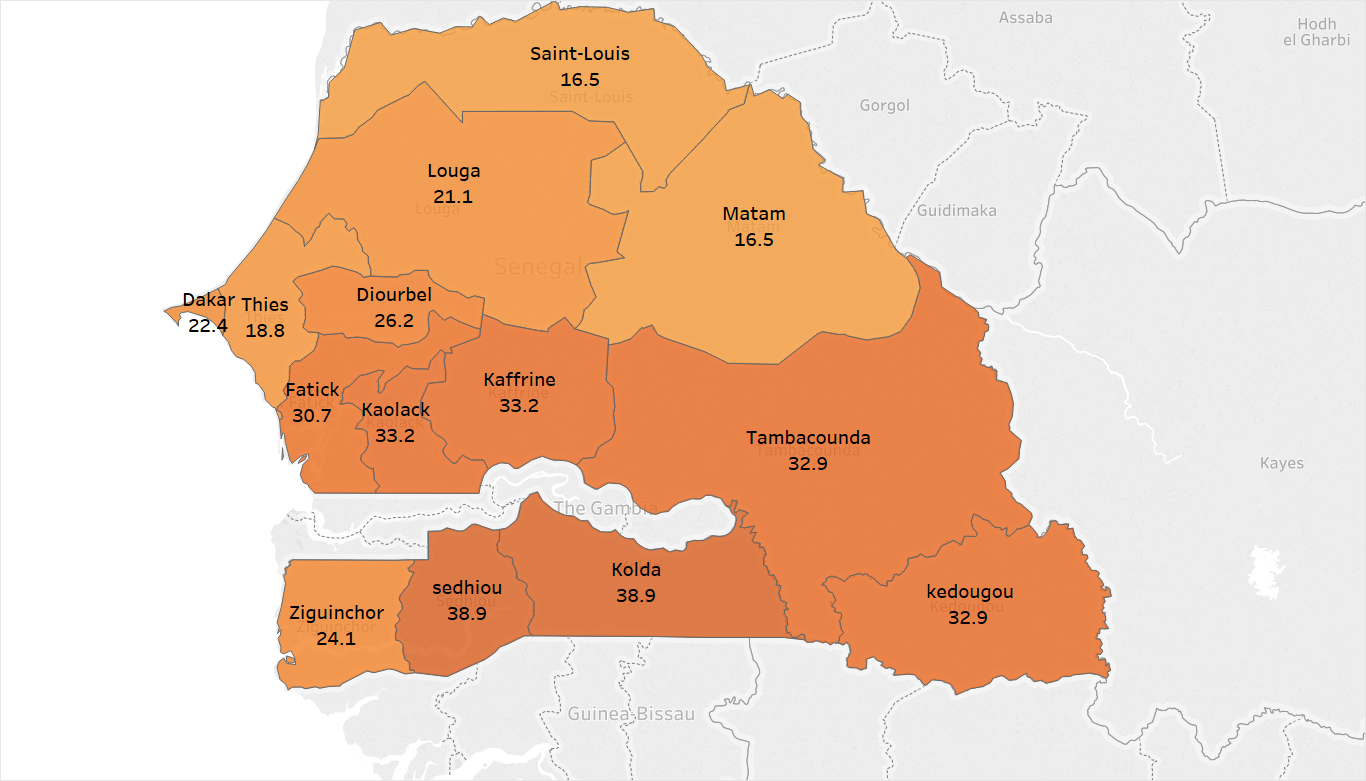


## **Supplementary Appendix Figure 5B**: 2005 stunting prevalence by region


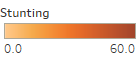

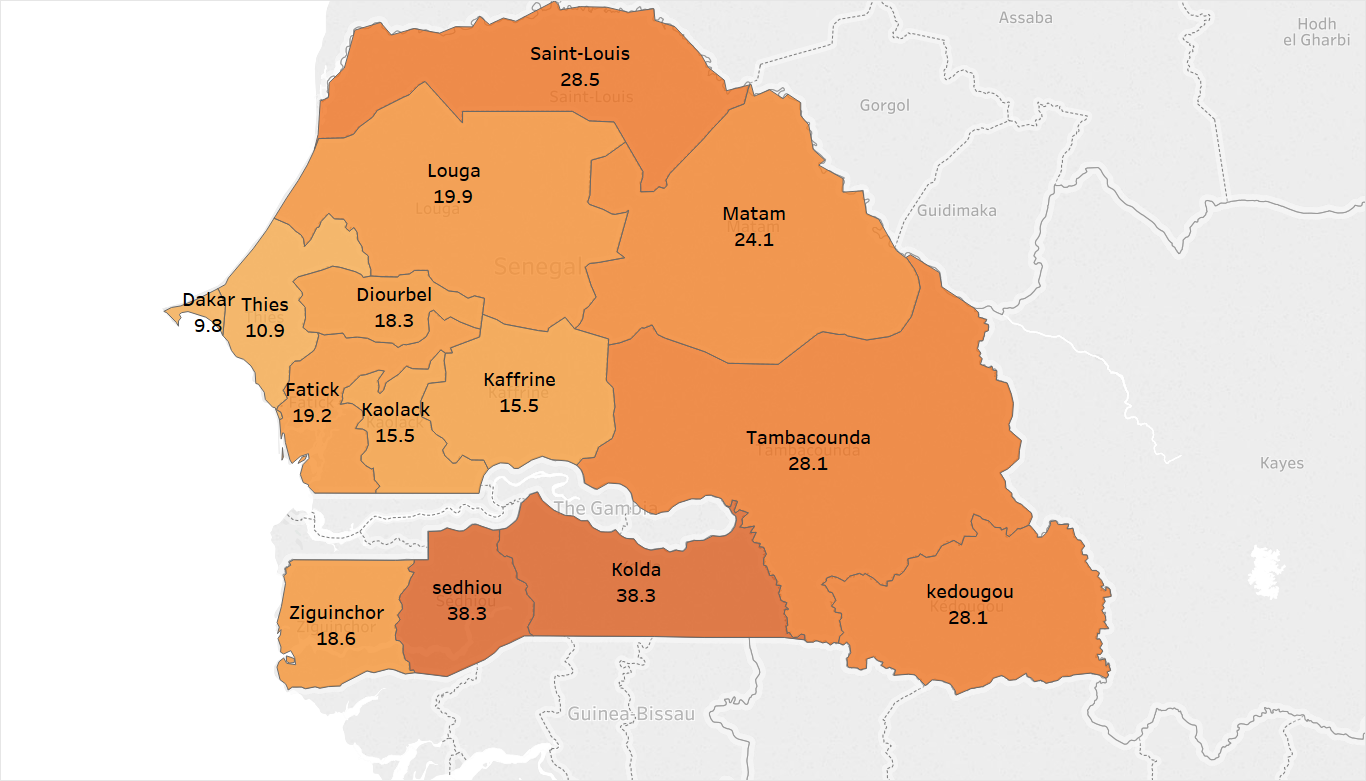


## **Supplementary Appendix Figure 5C**: 2017 stunting prevalence by region


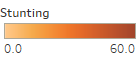

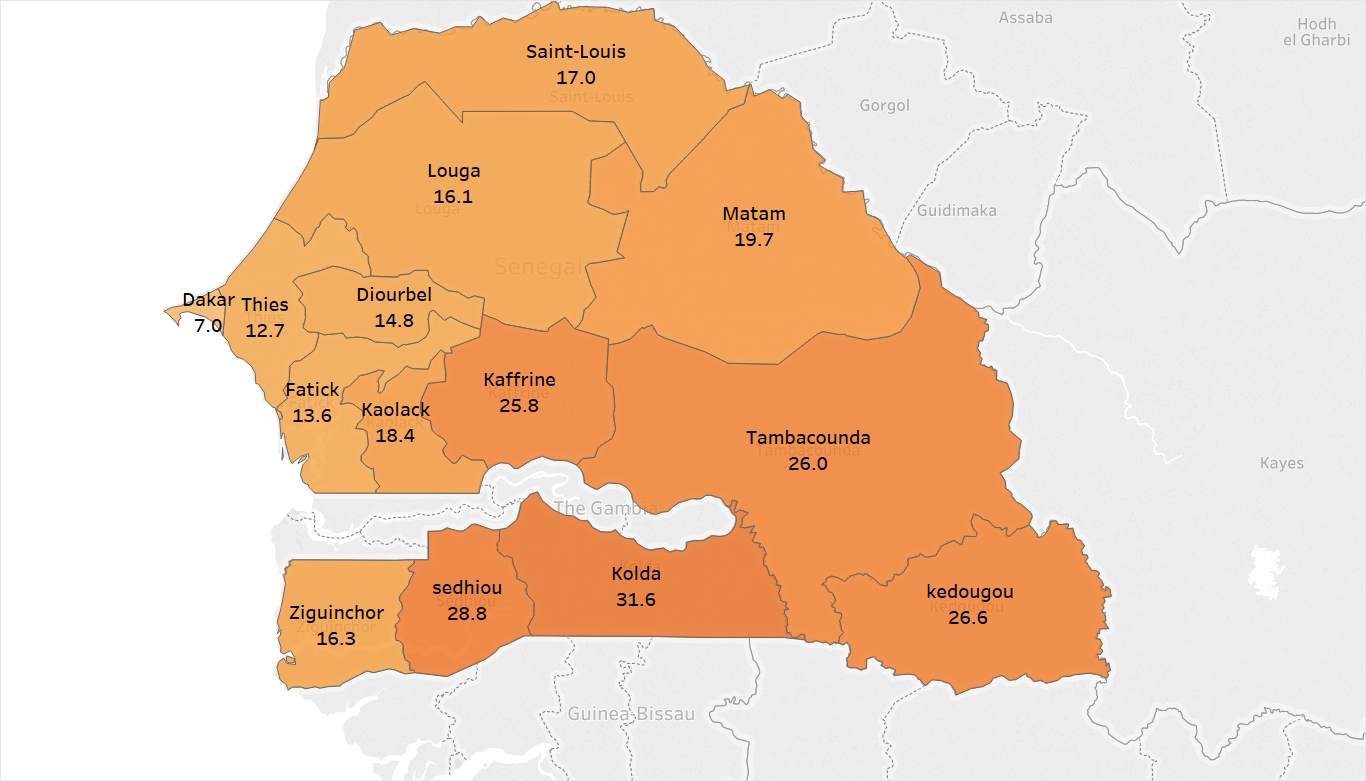


## **Supplementary Appendix Figure 6A:** Stunting prevalence by gender, 1992/93 – 2017


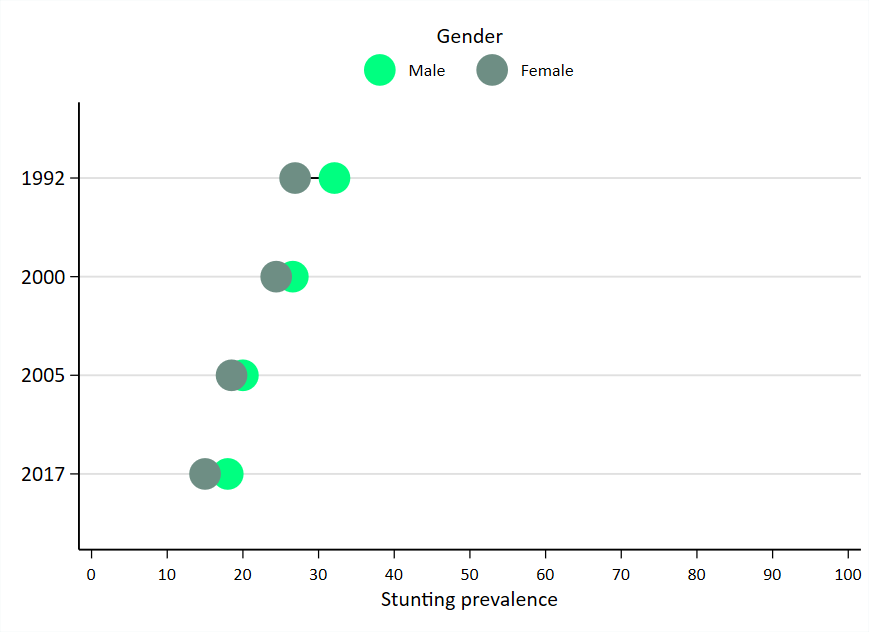


## **Supplementary Appendix Figure 6B**: Stunting prevalence by residential area and wealth quintiles, 1992/93 – 2017


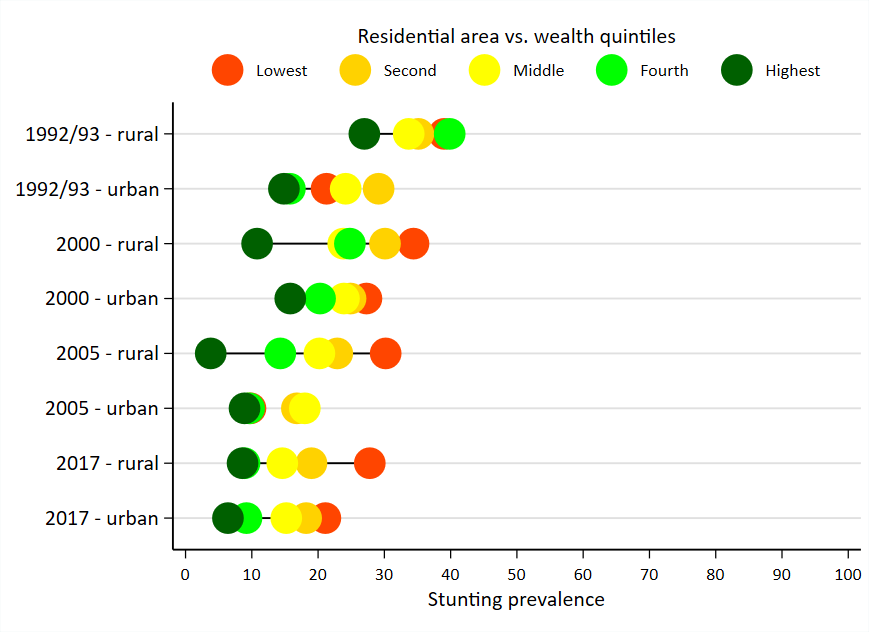


## **Supplementary Appendix Figure 7A**: Change in absolute SII by year in Senegal

## **Supplementary Appendix Figure 7B**: Change in relative CIX by year in Senegal

## **Supplementary Appendix Table 2:** Descriptive trends in stunting determinants in 1992/93 - 2017 in children <5 years

| **Domain/Indicator** | **DHS Survey Year** | | | | | | | |
| --- | --- | --- | --- | --- | --- | --- | --- | --- |
|  | **1992/93** | **2005** | **2017** | | **(2017 – 1992/93)** | | | |
|  | **(n = 1840)** | **(n = 1355)** | **(n = 5076)** | | **Change** | | **p-value** | |
| **Outcome** |  |  |  |  | |  | |  |
| Height for age z-score | -1.04 | -0.70 | -0.749 | | 0.29 | | <0.001 | |
|  |  |  |  | |  | |  | |
| Stunting | 24.95 | 17.71 | 15.034 | | -9.91 | | <0.001 | |
| % of children below -2sd |  |  |  | |  | |  | |
|  |  |  |  | |  | |  | |
| **Child Demographic** |  |  |  |  | |  | |  |
|  |  |  |  |  | |  | |  |
| Child sex (male) | 48.64 | 51.66 | 52.261 | | 3.62 | | 0.013 | |
| % of males |  |  |  | |  | |  | |
|  |  |  |  | |  | |  | |
| Child age | 18.80 | 19.21 | 22.542 | | 3.75 | | <0.001 | |
| (in months) |  |  |  | |  | |  | |
|  |  |  |  | |  | |  | |
| **Distal level** |  |  |  |  | |  | |  |
| **Basic causes & Income poverty** |  |  |  |  | |  | |  |
| Wealth Index (nine components using PCA) | 4.36 | 4.77 | 5.268 | | 0.91 | | <0.001 | |
| (0 - 10) |  |  |  | |  | |  | |
|  |  |  |  | |  | |  | |
| Mother year of education | 1.49 | 2.29 | 3.172 | | 1.69 | | <0.001 | |
|  |  |  |  | |  | |  | |
| Father year of education | 1.71 | 2.50 | 3.061 | | 1.35 | | <0.001 | |
|  |  |  |  | |  | |  | |
| **Intermediate level** |  |  |  |  | |  | |  |
| **Inadequate feeding practices and food insecurity** |  |  |  |  | |  | |  |
| Duration of breastfeeding | 13.03 | 13.55 | 10.817 | | -2.21 | | <0.001 | |
| (in months) |  |  |  | |  | |  | |
|  |  |  |  | |  | |  | |
| Early initiation of breastfeeding | 14.40 | 32.10 | 48.001 | | 33.60 | | <0.001 | |
| (% infants) |  |  |  | |  | |  | |
|  |  |  |  | |  | |  | |
| **Inadequate care and health services** |  |  |  |  | |  | |  |
| DPT vaccine | 71.24 | 81.77 | 90.911 | | 19.67 | | <0.001 | |
| (% infants with 3 doses) |  |  |  | |  | |  | |
|  |  |  |  | |  | |  | |
| Measles vaccine | 59.04 | 65.83 | 74.658 | | 15.62 | | <0.001 | |
| (% infants) |  |  |  | |  | |  | |
|  |  |  |  | |  | |  | |
| Live births attended by Skilled birth attendants | 51.30 | 55.34 | 72.470 | | 21.17 | | <0.001 | |
| (% women) |  |  |  | |  | |  | |
|  |  |  |  | |  | |  | |
| Antenatal care | 15.72 | 41.99 | 58.971 | | 43.25 | | <0.001 | |
| (% women with at least 4 visits) |  |  |  | |  | |  | |
|  |  |  |  | |  | |  | |
| Vitamin A supplementation | - | 75.17 | 61.494 | | - | |  | |
|  |  |  |  | |  | |  | |
|  |  |  |  | |  | |  | |
| **Unhealthy household environment** |  |  |  |  | |  | |  |
| Urbanization | 40.22 | 38.38 | 43.169 | | 2.95 | | 0.042 | |
| (% of urban population) |  |  |  | |  | |  | |
|  |  |  |  | |  | |  | |
| Open defecation | 38.79 | 24.99 | 14.931 | | -23.86 | | <0.001 | |
| (% population ) |  |  |  | |  | |  | |
|  |  |  |  | |  | |  | |
| Water source - piped | 45.26 | 56.88 | 70.485 | | 25.22 | | <0.001 | |
| (% of population) |  |  |  | |  | |  | |
|  |  |  |  | |  | |  | |
| Number of household members | 11.14 | 11.14 | 11.437 | | 0.30 | | 0.095 | |
|  |  |  |  | |  | |  | |
|  |  |  |  | |  | |  | |
| **Proximal level** |  |  |  |  | |  | |  |
| **Disease** |  |  |  |  | |  | |  |
| ARI infection | 33.64 | 31.90 | 22.072 | | -11.57 | | <0.001 | |
| (% under-5 population within last 2 weeks) |  |  |  | |  | |  | |
|  |  |  |  | |  | |  | |
| Diarrhea infection | 25.37 | 27.82 | 21.664 | | -3.70 | | 0.002 | |
| (% under-5 population within last 2 weeks) |  |  |  | |  | |  | |
|  |  |  |  | |  | |  | |
| **Child characteristics** |  |  |  |  | |  | |  |
| Low birthweight* | 8.60 | 11.73 | 11.774 | | 3.17 | | 0.019 | |
| (%, index child) |  |  |  | |  | |  | |
|  |  |  |  | |  | |  | |
| **Maternal characteristics** |  |  |  |  | |  | |  |
| Age | 27.98 | 28.18 | 28.984 | | 1.00 | | <0.001 | |
| (Mean, mothers 15-49) |  |  |  | |  | |  | |
|  |  |  |  | |  | |  | |
| Index births within last 5 years | 11.14 | 9.01 | 7.898 | | -3.24 | | <0.001 | |
| (% mothers <18 years) |  |  |  | |  | |  | |
|  |  |  |  | |  | |  | |
| Index births within last 5 years | 13.75 | 13.22 | 14.976 | | 1.23 | | 0.231 | |
| (% mothers >= 35 years) |  |  |  | |  | |  | |
|  |  |  |  | |  | |  | |
| Anemia during pregnancy | - | 57.96 | 52.140 | | - | |  | |
| (% women 15-49 years) |  |  |  | |  | |  | |
|  |  |  |  | |  | |  | |
| BMI level | 21.98 | 22.57 | - | | - | |  | |
| (Mean mothers 15-49 years) |  |  |  | |  | |  | |
|  |  |  |  | |  | |  | |
| Height | 162.36 | 162.93 | - | | - | |  | |
| (Mean mothers 15-49 years) |  |  |  | |  | |  | |
|  |  |  |  | |  | |  | |
| Parity | 4.08 | 3.49 | 3.312 | | -0.77 | | <0.001 | |
| (Total fertility rate) |  |  |  | |  | |  | |
|  |  |  |  | |  | |  | |
| Interpregnancy interval | 46.54 | 49.45 | 51.655 | | 5.11 | | <0.001 | |
| (in months) |  |  |  | |  | |  | |
|  |  |  |  | |  | |  | |

## **Supplementary Appendix Table 3:** Decomposition analysis for children among <5 years from 1992/93-2017

| **Factors** | **Estimated coefficient** | **Mean difference (2017 - 1992/93)** | **Predicted change in HAZ** | **Share of predicted change in (%)** | **Share of total change in HAZ (%)** |
| --- | --- | --- | --- | --- | --- |
| HAZ Score | - | 0.29 | 0.21 | 72.0% | Unexplained (28.0%) |
| Wealth index | 0.062 | 0.91 | 0.06 | 27.1% | 19.5% |
| Mother year of education | 0.013 | 1.69 | 0.02 | 10.4% | 7.5% |
| Father year of education | 0.016 | 1.35 | 0.02 | 10.3% | 7.4% |
| Skilled birth attendants | 0.069 | 0.21 | 0.01 | 7.0% | 5.0% |
| Antenatal care visits 4+ | 0.152 | 0.43 | 0.07 | 31.7% | 22.8% |
| Water source - piped | 0.093 | 0.25 | 0.02 | 11.3% | 8.1% |
| Early age pregnancy | -0.272 | -0.03 | 0.01 | 4.2% | 3.1% |
| Parity | 0.019 | -0.77 | -0.01 | -7.1% | -5.1% |
| Inter-pregnancy interval (in months) | 0.002 | 5.11 | 0.01 | 4.6% | 3.3% |
| Others | - | - | 0.00 | 0.2% | 0.2% |

## **Supplementary Appendix Figure 8A:** Decomposing predicted changes in HAZ among children <5 years (i.e. relative ranking of product coefficients for determinant domains) from 1992/93-2017

## **Supplementary Appendix Table 4:** Decomposition analysis for children among <24 months from 1992/93-2017

| **Factors** | **Estimated coefficient** | **Mean difference (2017 - 1992/93)** | **Predicted change in HAZ** | **Share of predicted change in (%)** | **Share of total change in HAZ (%)** |
| --- | --- | --- | --- | --- | --- |
| HAZ Score | - | 0.717 | 0.43 | 60.1% | Unexplained (39.9%) |
| Wealth index | 0.074 | 0.963 | 0.07 | 16.6% | 10.0% |
| Mother year of education | 0.019 | 1.517 | 0.03 | 6.6% | 4.0% |
| Father year of education | 0.015 | 1.154 | 0.02 | 4.0% | 2.4% |
| DPT vaccine | 0.616 | 0.108 | 0.07 | 15.4% | 9.3% |
| Skilled birth attendants | 0.151 | 0.162 | 0.02 | 5.7% | 3.4% |
| Antenatal care visits 4+ | 0.241 | 0.450 | 0.11 | 25.1% | 15.1% |
| Reduced open defecation | -0.203 | -0.232 | 0.05 | 10.9% | 6.6% |
| Water source - piped | 0.209 | 0.230 | 0.05 | 11.1% | 6.7% |
| Reduction in household crowding | -0.018 | -0.170 | 0.003 | 0.7% | 0.4% |
| Early age pregnancy | -0.333 | -0.055 | 0.02 | 4.2% | 2.5% |
| Others | - | - | -0.001 | -0.3% | -0.2% |

## **Supplementary Appendix Figure 8B:** Decomposing predicted changes in HAZ among children <24 months (i.e. relative ranking of product coefficients for determinant domains) from 1992/93-2017

## **Supplementary Appendix Table 5:** Difference-in-differences multivariable regression for children under-5 years from 1992/93 - 2017

| **Domain/Indicator** | **Outcome = HAZ**  **(Height for age z-score for under-5 children)** | |
| --- | --- | --- |
|  | **Period 1992/93 - 2017** | |
|  | **Bivariate regression coefficient** | **Final multivariable regression coefficient*** |
|  | b estimate (95% CI)  *p*-value | b estimate (95% CI)  *p*-value |
|  |  |  |
| **Distal level** | | |
| **Basic causes & Income poverty** | | |
| Wealth Index (six components using PCA) | 0.085 (0.073, 0.097) | 0.042 (0.029, 0.056) |
| (0 - 10) | <0.0001 | <0.0001 |
|  |  |  |
| Wealth Index# Year | 0.001 (-0.0004, 0.002) |  |
| (0 - 10) | 0.319 |  |
|  |  |  |
| Mother years of schooling | 0.054 (0.045, 0.064) | 2.49 (0.406, 4.57) |
|  | <0.0001 | 0.019 |
|  |  |  |
| Maternal education # year | -0.002 (-0.003, -0.0004) | -0.001 (-0.002, 0.0002) |
|  | 0.006 | 0.020 |
|  |  |  |
| Father years of schooling | 0.046 (0.037, 0.055) | _ |
|  | <0.0001 |  |
|  |  |  |
| Father education # Year | -0.001 (-0.002, -0.0003) | _ |
|  | 0.006 |  |
|  |  |  |
| **% of residual variance explained by covariates** | | **10.8%** |
| **Intermediate level** | | |
| **Inadequate feeding practices and food insecurity** | | |
| Duration of breastfeeding | -0.054 (-0.061, -0.047) | _ |
| (in months) | <0.0001 |  |
|  |  |  |
| Duration of breastfeed # Year | 0.002 (0.001, 0.002) | _ |
|  | <0.0001 |  |
|  |  |  |
| Early initiation of breastfeeding | -0.039 (-0.119, 0.040) | _ |
| (% infants) | 0.335 |  |
|  |  |  |
| Early initiation of breastfeeding # Year | -0.003 (-0.011, 0.005) | _ |
| (% infants) | 0.451 |  |
|  |  |  |
| **Inadequate care and health services** | | |
| Live births attended by Skilled birth attendants | 0.441 (0.359, 0.523) | _ |
| (% women) | <0.0001 |  |
|  |  |  |
| Live births attended by Skilled birth attendants # Year | -0.004 (-0.012, 0.004) | _ |
|  | 0.296 |  |
|  |  |  |
| Antenatal care | 0.365 (0.278, 0.452) | 0.185 (0.106, 0.263) |
| (% women with at least 4 visits) | <0.0001 | <0.0001 |
|  |  |  |
| Antenatal care # Year | -0.007 (-0.017, 0.002) | _ |
|  | 0.142 |  |
|  |  |  |
| total number of government hospitals per 10000 population | _ | _ |
|  |  |  |
|  |  |  |
| total number of government hospitals per 10000 population # Year | _ | _ |
|  |  |  |
|  |  |  |
| total number of primary health care centers per 10000 population | _ | _ |
|  |  |  |
|  |  |  |
| total number of primary health care centers per 10000 population # Year | _ | _ |
|  |  |  |
|  |  |  |
| total number of health posts or lower level health facilities per 10000 population | _ | _ |
|  |  |  |
|  |  |  |
| total number of health posts or lower level health facilities per 10000 population # Year | _ | _ |
|  |  |  |
|  |  |  |
| Outreach clinics held per 10000 population. | _ | _ |
|  |  |  |
|  |  |  |
| Outreach clinics held per 10000 population # Year | _ | _ |
|  |  |  |
|  |  |  |
| Number of mother group meetings held per 10000 population. | _ | _ |
|  |  |  |
|  |  |  |
| Number of mother group meetings held per 10000 population # Year | _ | _ |
|  |  |  |
|  |  |  |
| **Unhealthy household environment** | | |
| Urbanization | 0.484 (0.394, 0.573) | 0.122 (0.026, 0.218) |
| (% of urban population) | <0.0001 | 0.013 |
|  |  |  |
| Urbanization # Year | -0.003 (-0.011, 0.005) | _ |
|  | 0.489 |  |
|  |  |  |
| Open defecation | -0.360 (-0.448, -0.271) | _ |
| (% population ) | <0.0001 |  |
|  |  |  |
| Open defecation # Year | 0.002 (-0.006, 0.010) | _ |
|  | 0.600 |  |
|  |  |  |
| Water source - piped | 0.416 (0.331, 0.502) | _ |
| (% of population) | <0.0001 |  |
|  |  |  |
| Water source - piped # Year | -0.004 (-0.012, 0.004) | _ |
|  | 0.303 |  |
|  |  |  |
| Household crowding | 0.002 (-0.005, 0.008) | _ |
|  | 0.633 |  |
|  |  |  |
| Household crowding # Year | 0.0003 (-0.0003, 0.001) | _ |
|  | 0.314 |  |
|  |  |  |
| **% of residual variance explained by covariates** | | **11.2%** |
| **Proximal level** | | |
| **Disease** | | |
| Acute Respiratory incidence/ reports | 0.076 (-0.038, 0.190) | _ |
| (% under-5 population within last 2 weeks) | 0.189 |  |
|  |  |  |
| Acute Respiratory incidence # Year | -0.006 (-0.016, 0.003) | _ |
|  | 0.196 |  |
|  |  |  |
| Diarrhea incidence/reports | -0.067 (-0.156, 0.021) | _ |
| (% under-5 population within last 2 weeks) | 0.135 |  |
|  |  |  |
| Diarrhea incidence # Year | 0.001 (-0.007, 0.009) | _ |
|  | 0.827 |  |
|  |  |  |
| **Maternal characteristics** | | |
| Age | 0.001 (-0.004, 0.006) | _ |
| (Mean, mothers 15-49) | 0.676 |  |
|  |  |  |
| Age# Year | 0.0004 (-0.00002, 0.001) | _ |
|  | 0.058 |  |
|  |  |  |
| Index births within last 5 years | -0.355 (-0.475, -0.235) | -0.288 (-0.403, -0.173) |
| (% mothers <18 years) | <0.0001 | <0.0001 |
|  |  |  |
| Index birth within last 5 years # Year | 0.003 (-0.008, 0.014) | _ |
|  | 0.603 |  |
|  |  |  |
| Index births within last 5 years | -0.050 (-0.152, 0.052) | _ |
| (% mothers >= 35 years) | 0.334 |  |
|  |  |  |
| Index birth within last 5 years # Year | 0.004 (-0.007, 0.014) | _ |
|  | 0.496 |  |
|  |  |  |
| Anemia percentage among estimated pregnancies | _ | _ |
|  |  |  |
|  |  |  |
| Anemia percentage among estimated pregnancies # Year | _ | _ |
|  |  |  |
|  |  |  |
| BMI level | _ | _ |
| (Mean mothers 15-49 years) |  |  |
|  |  |  |
| BMI level # Year | _ | _ |
|  |  |  |
|  |  |  |
| Height | _ | _ |
| (Mean mothers 15-49 years) |  |  |
|  |  |  |
| Height # Year | _ | _ |
|  |  |  |
|  |  |  |
| Parity | -0.035 (-0.049, -0.020) | _ |
| (Total fertility rate) | <0.0001 |  |
|  |  |  |
| Parity # Year | -0.0004 (-0.002, 0.001) | _ |
|  | 0.520 |  |
|  |  |  |
| Interpregnancy interval | 0.004 (0.003, 0.005) | 0.002 (0.001, 0.004) |
| (in months) | <0.0001 | 0.001 |
|  |  |  |
| Inter pregnancy intervals # Year | 0.00004 (-0.0001, 0.0002) |  |
|  | 0.488 | -- |
|  |  |  |
| **% of residual variance explained by covariates** | | **11.6%** |
| **Time** | | |
| **Time** | | |
| Year | 0.010 (0.006, 0.014) | -0.014 (-0.023, -0.005) |
|  | <0.0001 | 0.002 |
|  |  |  |
| * Adjusted for child age, sex, and province |  |  |

## **Supplementary Appendix Table 6:** Difference-in-differences multivariable regression for children aged 24-59 months from 1992/93 - 2017

| **Domain/Indicator** | **Outcome = HAZ** | |
| --- | --- | --- |
|  | **(Height for age z-score for children aged 24-59 months)** | |
|  | **Period 1992/93 - 2017** | |
|  | **Bivariate regression coefficient** | **Final multivariable regression coefficient*** |
|  | b estimate (95% CI) | b estimate (95% CI) |
|  | *p*-value | *p*-value |
|  |  |  |
| **Distal level** | | |
| **Basic causes & Income poverty** | | |
| Wealth Index (six components using PCA) | 0.121 (0.107, 1.35) | 0.057 (0.037, 0.078) |
| (0 - 10) | <0.0001 | <0.0001 |
|  |  |  |
| Wealth Index#Year | 0.001 (-0.0004, 0.002) | _ |
| (0 - 10) | 0.319 |  |
|  |  |  |
| Mother years of schooling | 0.073 (0.060, 0.085) | 1.81 (-2.12, 5.73) |
|  | <0.0001 | 0.367 |
|  |  |  |
| Maternal education # year | -0.002 (-0.003, -0.0004) | -0.001 (-0.003, 0.001) |
|  | 0.006 | 0.373 |
|  |  |  |
| Father years of schooling | 0.061 (0.049, 0.073) | 0.938 (-1.97, 3.85) |
|  | <0.0001 | 0.527 |
|  |  |  |
| Father education # Year | -0.001 (-0.002, -0.0004) | -0.005 (-0.002, 0.001) |
|  | 0.006 | 0.523 |
|  |  |  |
| **% of residual variance explained by covariates** | | **18.6%** |
| **Intermediate level** | | |
| **Inadequate feeding practices and food insecurity** | | |
| Duration of breastfeeding | -0.048 (-0.066, -0.030) | _ |
| (in months) | <0.0001 |  |
|  |  |  |
| Duration of breastfeed # Year | 0.002 (0.001, 0.004) | _ |
|  | 0.004 |  |
|  |  |  |
| Early initiation of breastfeeding | -0.008 (-0.128, 0.111) | _ |
| (% infants) | 0.889 |  |
|  |  |  |
| Early initiation of breastfeeding # Year | -0.005 (-0.019, 0.008) | _ |
| (% infants) | 0.417 |  |
|  |  |  |
| **Inadequate care and health services** | | |
| Live births attended by Skilled birth attendants | 0.646 (0.535, 0.757) | 38.9 (11.6, 60.3) |
| (% women) | <0.0001 | 0.004 |
|  |  |  |
| Live births attended by Skilled birth attendants # Year | -0.021 (-0.032, -0.010) | -0.018 (-0.030, -0.006) |
|  | <0.0001 | 0.004 |
|  |  |  |
| Antenatal care | 0.359 (0.243, 0.476) | 33.1 (5.60, 60.6) |
| (% women with at least 4 visits) | <0.0001 | 0.018 |
|  |  |  |
| Antenatal care # Year | -0.019 (-0.032, -0.006) | -0.016 (-0.030, -0.003) |
|  | 0.003 | 0.019 |
|  |  |  |
| total number of government hospitals per 10000 population | _ | _ |
|  |  |  |
|  |  |  |
| total number of government hospitals per 10000 population # Year | _ | _ |
|  |  |  |
|  |  |  |
| total number of primary health care centers per 10000 population | _ | _ |
|  |  |  |
|  |  |  |
| total number of primary health care centers per 10000 population # Year | _ | _ |
|  |  |  |
|  |  |  |
| total number of health posts or lower level health facilities per 10000 population | _ | _ |
|  |  |  |
|  |  |  |
| total number of health posts or lower level health facilities per 10000 population # Year | _ | _ |
|  |  |  |
|  |  |  |
| Outreach clinics held per 10000 population. | _ | _ |
|  |  |  |
|  |  |  |
| Outreach clinics held per 10000 population # Year | _ | _ |
|  |  |  |
|  |  |  |
| Number of mother group meetings held per 10000 population. | _ | _ |
|  |  |  |
|  |  |  |
| Number of mother group meetings held per 10000 population # Year | _ | _ |
|  |  |  |
|  |  |  |
| **Unhealthy household environment** | | |
| Urbanization | 0.690 (0.585, 0.794) | _ |
| (% of urban population) | <0.0001 |  |
|  |  |  |
| Urbanization # Year | -0.011 (-0.021, -0.001) | _ |
|  | 0.030 |  |
|  |  |  |
| Open defecation | -0.663 (0.786, -0.541) | -0.156 (-0.303, -0.009) |
| (% population ) | <0.0001 | 0.037 |
|  |  |  |
| Open defecation # Year | 0.007 (-0.003, 0.018) | _ |
|  | 0.182 |  |
|  |  |  |
| Water source - piped | 0.670 (0.557, 0.782) | _ |
| (% of population) | <0.0001 |  |
|  |  |  |
| Water source - piped # Year | -0.020 (-0.307, -0.010) | _ |
|  | <0.0001 |  |
|  |  |  |
| Household crowding | -0.007 (-0.017, 0.002) | _ |
|  | 0.128 |  |
|  |  |  |
| Household crowding # Year | 0.001 (0.0002, 0.002) | _ |
|  | 0.019 |  |
|  |  |  |
| **% of residual variance explained by covariates** | | **19.1%** |
| **Proximal level** | | |
| **Disease** | | |
| Acute Respiratory incidence/ reports | -0.071 (-0.261, 0.119) | _ |
| (% under-5 population within last 2 weeks) | 0.462 |  |
|  |  |  |
| Acute Respiratory incidence # Year | -0.004 (-0.021, 0.013) | _ |
|  | 0.632 |  |
|  |  |  |
| Diarrhea incidence/reports | -0.201 (-0.343, -0.060) | _ |
| (% under-5 population within last 2 weeks) | 0.005 |  |
|  |  |  |
| Diarrhea incidence # Year | 0.003 (-0.013, 0.018) | _ |
|  | 0.737 |  |
|  |  |  |
| **Maternal characteristics** | | |
| Age | 0.0192 (0.012, 0.026) | _ |
| (Mean, mothers 15-49) | <0.0001 |  |
|  |  |  |
| Age# Year | -0.0001 (-0.001, 0.001) | _ |
|  | 0.829 |  |
|  |  |  |
| Index births within last 5 years | -0.536 (-0.742, -0.330) | -0.411 (-0.647, -0.176) |
| (% mothers <18 years) | <0.0001 | 0.001 |
|  |  |  |
| Index birth within last 5 years # Year | 0.002 (-0.017, 0.020) | _ |
|  | 0.850 |  |
|  |  |  |
| Index births within last 5 years | 0.119 (-0.005, 0.242) | _ |
| (% mothers >= 35 years) | 0.059 |  |
|  |  |  |
| Index birth within last 5 years # Year | -0.0001 (-0.014, 0.014) | _ |
|  | 0.993 |  |
|  |  |  |
| Anemia percentage among estimated pregnancies | _ | _ |
|  |  |  |
|  |  |  |
| Anemia percentage among estimated pregnancies # Year | _ | _ |
|  |  |  |
|  |  |  |
| BMI level | _ | _ |
| (Mean mothers 15-49 years) |  |  |
|  |  |  |
| BMI level # Year | _ | _ |
|  |  |  |
|  |  |  |
| Height | _ | _ |
| (Mean mothers 15-49 years) |  |  |
|  |  |  |
| Height # Year | _ | _ |
|  |  |  |
|  |  |  |
| Parity | -0.021 (-0.042, -0.001) | 0.033 (0.006, 0.061) |
| (Total fertility rate) | 0.037 | 0.018 |
|  |  |  |
| Parity # Year | -0.001 (-0.003, 0.001) | _ |
|  | 0.348 |  |
|  |  |  |
| Interpregnancy interval | 0.005 (0.003, 0.008) | 0.004 (0.001, 0.007) |
| (in months) | <0.0001 | 0.004 |
|  |  |  |
| Inter pregnancy intervals # Year | -0.008 (-0.431, 0.415) |  |
|  | 0.972 | -- |
|  |  |  |
| **% of residual variance explained by covariates** | | **20.2%** |
| **Time** | | |
| **Time** | | |
| Year | 0.027 (0.0222, 0.032) | 0.034 (0.026, 0.043) |
|  | <0.0001 | <0.0001 |
|  |  |  |
| * Adjusted for child age, sex, and province |  |  |

## **Supplementary Appendix Table 7** Difference-in-differences multivariable regression for children aged 6-23 months from 1992/93 - 2017

| **Domain/Indicator** | **Outcome = HAZ** | |
| --- | --- | --- |
|  | **(Height for age z-score for children aged 6-23 months)** | |
|  | **Period 1992/93 - 2017** | |
|  | **Bivariate regression coefficient** | **Final multivariable regression coefficient*** |
|  | b estimate (95% CI) | b estimate (95% CI) |
|  | *p*-value | *p*-value |
|  |  |  |
| **Distal level** | | |
| **Basic causes & Income poverty** | | |
| Wealth Index (six components using PCA) | 0.086 (0.069, 0.103) | 0.035 (0.014, 0.057) |
| (0 - 10) | <0.0001 | 0.001 |
|  |  |  |
| Wealth Index#Year | 0.001 (-0.0004, 0.002) | _ |
| (0 - 10) | 0.319 |  |
|  |  |  |
| Mother years of schooling | 0.55 (0.041, 0.070) | _ |
|  | <0.0001 |  |
|  |  |  |
| Maternal education # year | -0.002 (-0.003, -0.0004) | _ |
|  | 0.006 |  |
|  |  |  |
| Father years of schooling | 0.046 (0.032, 0.060) | _ |
|  | <0.0001 |  |
|  |  |  |
| Father education # Year | -0.001 (-0.002, -0.0004) | _ |
|  | 0.006 |  |
|  |  |  |
| **% of residual variance explained by covariates** | | **12.0%** |
| **Intermediate level** | | |
| **Inadequate feeding practices and food insecurity** | | |
| Duration of breastfeeding | -0.075 (-0.086, -0.063) | _ |
| (in months) | <0.0001 |  |
|  |  |  |
| Duration of breastfeed # Year | 0.002 (0.001, 0.003) | _ |
|  | <0.0001 |  |
|  |  |  |
| Early initiation of breastfeeding | -0.017 (-0.125, 0.091) | _ |
| (% infants) | 0.755 |  |
|  |  |  |
| Early initiation of breastfeeding # Year | -0.008 (-0.019, 0.003) | _ |
| (% infants) | 0.147 |  |
|  |  |  |
| **Inadequate care and health services** | | |
| Live births attended by Skilled birth attendants | 0.439 (0.326, 0.552) | 0.039 (-0.081, 0.159) |
| (% women) | <0.0001 | 0.522 |
|  |  |  |
| Live births attended by Skilled birth attendants # Year | -0.0003 (-0.011, 0.010) | _ |
|  | 0.952 |  |
|  |  |  |
| Antenatal care | 0.423 (0.304, 0.541) | 0.275 (0.162, 0.387) |
| (% women with at least 4 visits) | <0.0001 | <0.0001 |
|  |  |  |
| Antenatal care # Year | 0.002 (-0.011, 0.015) | _ |
|  | 0.785 |  |
|  |  |  |
| total number of government hospitals per 10000 population | _ | _ |
|  |  |  |
|  |  |  |
| total number of government hospitals per 10000 population # Year | _ | _ |
|  |  |  |
|  |  |  |
| total number of primary health care centers per 10000 population | _ | _ |
|  |  |  |
|  |  |  |
| total number of primary health care centers per 10000 population # Year | _ | _ |
|  |  |  |
|  |  |  |
| total number of health posts or lower level health facilities per 10000 population | _ | _ |
|  |  |  |
|  |  |  |
| total number of health posts or lower level health facilities per 10000 population # Year | _ | _ |
|  |  |  |
|  |  |  |
| Outreach clinics held per 10000 population. | _ | _ |
|  |  |  |
|  |  |  |
| Outreach clinics held per 10000 population # Year | _ | _ |
|  |  |  |
|  |  |  |
| Number of mother group meetings held per 10000 population. | _ | _ |
|  |  |  |
|  |  |  |
| Number of mother group meetings held per 10000 population # Year | _ | _ |
|  |  |  |
|  |  |  |
| **Unhealthy household environment** | | |
| Urbanization | -0.499 (0.378, 0.621) | 0.180 (0.047, 0.312) |
| (% of urban population) | <0.0001 | 0.008 |
|  |  |  |
| Urbanization # Year | 0.0004 (-0.010, 0.112) | _ |
|  | 0.936 |  |
|  |  |  |
| Open defecation | -0.344 (-0.465, -0.223) | _ |
| (% population ) | <0.0001 |  |
|  |  |  |
| Open defecation # Year | -0.001 (-0.012, 0.010) | _ |
|  | 0.851 |  |
|  |  |  |
| Water source - piped | 0.387 (0.279, 0.495) | _ |
| (% of population) | <0.0001 |  |
|  |  |  |
| Water source - piped # Year | -0.001 (-0.011, 0.009) | _ |
|  | 0.859 |  |
|  |  |  |
| Household crowding | -0.003 (-0.012, 0.006) | _ |
|  | 0.479 |  |
|  |  |  |
| Household crowding # Year | 0.00005 (-0.001, 0.001) | _ |
|  | 0.991 |  |
|  |  |  |
| **% of residual variance explained by covariates** | | **13.4%** |
| **Proximal level** | | |
| **Disease** | | |
| Acute Respiratory incidence/ reports | 0.178 (0.014, 0.343) | 0.147 (-0.008, 0.302) |
| (% under-5 population within last 2 weeks) | 0.033 | 0.064 |
|  |  |  |
| Acute Respiratory incidence # Year | -0.003 (-0.017, 0.012) | _ |
|  | 0.719 |  |
|  |  |  |
| Diarrhea incidence/reports | -0.105 (-0.212, 0.002) | _ |
| (% under-5 population within last 2 weeks) | 0.055 |  |
|  |  |  |
| Diarrhea incidence # Year | 0.006 (-0.004, 0.015) | _ |
|  | 0.275 |  |
|  |  |  |
| **Maternal characteristics** | | |
| Age | 0.005 (-0.003, 0.013) | 0.022 (0.007, 0.038) |
| (Mean, mothers 15-49) | 0.199 | 0.005 |
|  |  |  |
| Age# Year | 0.0004 (-0.0003, 0.001) | _ |
|  | 0.272 |  |
|  |  |  |
| Index births within last 5 years | -0.288 (-0.446, -0.130) | _ |
| (% mothers <18 years) | <0.0001 |  |
|  |  |  |
| Index birth within last 5 years # Year | -0.002 (-0.018, 0.014) | _ |
|  | 0.777 |  |
|  |  |  |
| Index births within last 5 years | -0.076 (-0.248, 0.096) | _ |
| (% mothers >= 35 years) | 0.387 |  |
|  |  |  |
| Index birth within last 5 years # Year | 0.004 (-0.012, 0.020) | _ |
|  | 0.608 |  |
|  |  |  |
| Anemia percentage among estimated pregnancies | _ | _ |
|  |  |  |
|  |  |  |
| Anemia percentage among estimated pregnancies # Year | _ | _ |
|  |  |  |
|  |  |  |
| BMI level | _ | _ |
| (Mean mothers 15-49 years) |  |  |
|  |  |  |
| BMI level # Year | _ | _ |
|  |  |  |
|  |  |  |
| Height | _ | _ |
| (Mean mothers 15-49 years) |  |  |
|  |  |  |
| Height # Year | _ | _ |
|  |  |  |
|  |  |  |
| Parity | -0.041 (-0.063, -0.019) | -0.066 (-0.111, -0.021) |
| (Total fertility rate) | <0.0001 | 0.004 |
|  |  |  |
| Parity # Year | -0.0003 (-0.002, 0.002) | _ |
|  | 0.762 |  |
|  |  |  |
| Interpregnancy interval | 0.004 (0.002, 0.006) | _ |
| (in months) | 0.001 |  |
|  |  |  |
| Inter pregnancy intervals # Year | 0.0001 (-0.0001, 0.0003) |  |
|  | 0.484 | -- |
|  |  |  |
| **% of residual variance explained by covariates** | | **15.8%** |
| **Time** | | |
| **Time** | | |
| Year | 0.008 (0.003, 0.013) | -0.028 (-0.029, 0.009) |
|  | 0.002 | 0.331 |
|  |  |  |
| * Adjusted for child age, sex, and province |  |  |

## **Supplementary Appendix Table 8**: Difference-in-differences multivariable regression for children under 6 months from 1992/93 - 2017

| **Domain/Indicator** | **Outcome = HAZ** | |
| --- | --- | --- |
|  | **(Height for age z-score for children under 6 months)** | |
|  | **Period 1992/93 - 2017** | |
|  | **Bivariate regression coefficient** | **Final multivariable regression coefficient*** |
|  | b estimate (95% CI) | b estimate (95% CI) |
|  | *p*-value | *p*-value |
|  |  |  |
| **Distal level** | | |
| **Basic causes & Income poverty** | | |
| Wealth Index (six components using PCA) | 0.042 (0.008, 0.077) | 0.017 (-0.008, 0.042) |
| (0 - 10) | 0.015 | 0.068 |
|  |  |  |
| Wealth Index#Year | 0.001 (-0.0005, 0.002) | _ |
| (0 - 10) | 0.319 |  |
|  |  |  |
| Mother years of schooling | 0.0167 (-0.009, 0.042) | _ |
|  | 0.203 |  |
|  |  |  |
| Maternal education # year | -0.002 (-0.003, -0.0004) | _ |
|  | 0.006 |  |
|  |  |  |
| Father years of schooling | 0.026 (0.005, 0.047) | _ |
|  | 0.014 |  |
|  |  |  |
| Father education # Year | -0.001 (-0.002, -0.0004) | _ |
|  | 0.006 |  |
|  |  |  |
| **% of residual variance explained by covariates** | | **2.5%** |
| **Intermediate level** | | |
| **Inadequate feeding practices and food insecurity** | | |
| Duration of breastfeeding | 0.053 (0.014, 0.091) | _ |
| (in months) | 0.007 |  |
|  |  |  |
| Duration of breastfeed # Year | -0.002 (-0.006, 0.002) | _ |
|  | 0.381 |  |
|  |  |  |
| Early initiation of breastfeeding | -0.037 (-0.230, 0.156) | _ |
| (% infants) | 0.705 |  |
|  |  |  |
| Early initiation of breastfeeding # Year | -0.005 (-0.025, 0.014) | _ |
| (% infants) | 0.601 |  |
|  |  |  |
| **Inadequate care and health services** | | |
| Live births attended by Skilled birth attendants | 0.156 (-0.052, 0.364) | _ |
| (% women) | 0.142 |  |
|  |  |  |
| Live births attended by Skilled birth attendants # Year | -0.007 (-0.023, 0.009) | _ |
|  | 0.365 |  |
|  |  |  |
| Antenatal care | 0.387 (0.142, 0.632) | 0.297 (0.094, 0.499) |
| (% women with at least 4 visits) | 0.002 | 0.004 |
|  |  |  |
| Antenatal care # Year | -0.009 (-0.033, 0.014) | _ |
|  | 0.443 |  |
|  |  |  |
| total number of government hospitals per 10000 population | _ | _ |
|  |  |  |
|  |  |  |
| total number of government hospitals per 10000 population # Year | _ | _ |
|  |  |  |
|  |  |  |
| total number of primary health care centers per 10000 population | _ | _ |
|  |  |  |
|  |  |  |
| total number of primary health care centers per 10000 population # Year | _ | _ |
|  |  |  |
|  |  |  |
| total number of health posts or lower level health facilities per 10000 population | _ | _ |
|  |  |  |
|  |  |  |
| total number of health posts or lower level health facilities per 10000 population # Year | _ | _ |
|  |  |  |
|  |  |  |
| Outreach clinics held per 10000 population. | _ | _ |
|  |  |  |
|  |  |  |
| Outreach clinics held per 10000 population # Year | _ | _ |
|  |  |  |
|  |  |  |
| Number of mother group meetings held per 10000 population. | _ | _ |
|  |  |  |
|  |  |  |
| Number of mother group meetings held per 10000 population # Year | _ | _ |
|  |  |  |
|  |  |  |
| **Unhealthy household environment** | | |
| Urbanization | 0.249 (0.009, 0.488) | _ |
| (% of urban population) | 0.042 |  |
|  |  |  |
| Urbanization # Year | 0.002 (-0.014, 0.018) | _ |
|  | 0.840 |  |
|  |  |  |
| Open defecation | -0.113 (-0.317, 0.091) | _ |
| (% population ) | 0.278 |  |
|  |  |  |
| Open defecation # Year | 0.009 (-0.007, 0.026) | _ |
|  | 0.274 |  |
|  |  |  |
| Water source - piped | 0.141 (-0.068, 0.350) | _ |
| (% of population) | 0.186 |  |
|  |  |  |
| Water source - piped # Year | 0.001 (-0.015, 0.017) | _ |
|  | 0.881 |  |
|  |  |  |
| Household crowding | 0.009 (-0.005, 0.024) | _ |
|  | 0.216 |  |
|  |  |  |
| Household crowding # Year | 0.0003 (-0.001, 0.001) | _ |
|  | 0.542 |  |
|  |  |  |
| **% of residual variance explained by covariates** | | **3.4%** |
| **Proximal level** | | |
| **Disease** | | |
| Acute Respiratory incidence/ reports | -0.050 (0.0295, 0.195) | _ |
| (% under-5 population within last 2 weeks) | 0.688 |  |
|  |  |  |
| Acute Respiratory incidence # Year | -0.0003 (-0.020, 0.020) | _ |
|  | 0.976 |  |
|  |  |  |
| Diarrhea incidence/reports | 0.131 (-0.114, 0.376) | _ |
| (% under-5 population within last 2 weeks) | 0.293 |  |
|  |  |  |
| Diarrhea incidence # Year | 0.006 (-0.014, 0.025) | _ |
|  | 0.574 |  |
|  |  |  |
| **Maternal characteristics** | | |
| Age | 0.006 (-0.005, 0.018) | _ |
| (Mean, mothers 15-49) | 0.273 |  |
|  |  |  |
| Age# Year | 0.0003 (-0.001, 0.001) | _ |
|  | 0.517 |  |
|  |  |  |
| Index births within last 5 years | -0.328 (-0.601, 0.054) | -0.235 (-0.478, 0.009) |
| (% mothers <18 years) | 0.019 | 0.059 |
|  |  |  |
| Index birth within last 5 years # Year | 0.004 (-0.018, 0.026) | _ |
|  | 0.726 |  |
|  |  |  |
| Index births within last 5 years | -0.133 (-0.376, 0.110) | _ |
| (% mothers >= 35 years) | 0.284 |  |
|  |  |  |
| Index birth within last 5 years # Year | 0.325 (-44.6, 45.3) | _ |
|  | 0.989 |  |
|  |  |  |
| Anemia percentage among estimated pregnancies | _ | _ |
|  |  |  |
|  |  |  |
| Anemia percentage among estimated pregnancies # Year | _ | _ |
|  |  |  |
|  |  |  |
| BMI level | _ | _ |
| (Mean mothers 15-49 years) |  |  |
|  |  |  |
| BMI level # Year | _ | _ |
|  |  |  |
|  |  |  |
| Height | _ | _ |
| (Mean mothers 15-49 years) |  |  |
|  |  |  |
| Height # Year | _ | _ |
|  |  |  |
|  |  |  |
| Parity | -0.009 (-0.047, 0.030) | _ |
| (Total fertility rate) | 0.664 |  |
|  |  |  |
| Parity # Year | 0.001 (-0.002, 0.004) | _ |
|  | 0.528 |  |
|  |  |  |
| Interpregnancy interval | 0.0004 (-0.003, 0.004) | _ |
| (in months) | 0.810 |  |
|  |  |  |
| Inter pregnancy intervals # Year | 0.200 (-0.378, 0.779) |  |
|  | 0.496 | -- |
|  |  |  |
| **% of residual variance explained by covariates** | | **3.7%** |
| **Time** | | |
| **Time** | | |
| Year | -0.003 (-0.011, 0.005) | -0.007 (-0.016, 0.001) |
|  | 0.420 | 0.102 |
|  |  |  |
| * Adjusted for child age, sex, and province |  |  |

# **Supplementary Appendix 6:** Program and Policies

## **Supplementary Appendix Table 9:** Description of Acts/Law/Regulations, Policies, and Program from 1990-Present

| **ACTS/LAWS/REGULATIONS** | | |
| --- | --- | --- |
| 1. Presidential Decree: National Commission for the Fight Against Malnutrition / Commission Nationale de Lutte Contre la Malnutrition (CNLM)   (1994-2001) | Description | Resulting from a Presidential Decree issued in June, 1994, the Commission for the Fight Against Malnutrition was in charge of identifying and implementing practical and effective solutions to address malnutrition in Senegal (80). This decree lead to the creation of the Community Nutrition Project (PNC) and was followed by the establishment of the Cellule de Lutte Contre la Malnutrition (CLM), the coordinating body for nutrition housed within the Prime Minister’s office from 2001 onward. |
|  | Importance | Very important as the Government’s first attempt to implement a multi-stakeholder nutrition coordinating body |
| 1. Presidential Decree on Mandatory Iodization of Edible Salt   (2001-present) | Description | Following the 1995 Prime Minister’s Decree, which set standards for iodized salt production and distribution in Senegal, the Presidential Decree on Mandatory Iodization of Edible Salt was issued in 2001. The decree aims to ensure mandatory, universal iodization of all salt consumed in the country. This undertaking has been led by the CLM since 2006, in order to increase support/resources for universal salt iodization. By 2014, a GAIN-UNICEF study found that nearly 92% of salt distributed and 83% at production level met iodization standards, however the women of reproductive age sampled continued to be iodine deficient. Poor monitoring and enforcement of this legislation has limited progress, and the gap/discrepancy between quality of salt in production and that being consumed indicates that low-quality salt continues to be leaked into the local market by small salt producers (81,82). |
|  | Importance | Likely not important for reducing child stunting |
| 1. Decree 2009-872 on Fortification of Refined Vegetable Oils and Flour   (2009-present) | Description | This decree requires that all imported and domestically produced refined vegetable oils (e.g., palm, cotton, palm kernel, peanuts, sesame, sunflower, canola, corn, and soybean oil) be enriched with Vitamin A (83). It also stipulates that all soft wheat flour (both domestically produced and imported) must be enriched with iron and folic acid. A 2013 survey found 96% of the flour samples collected contained added iron and 97% of the oil samples contained vitamin A (84). |
|  | Importance | Recent initiative with insufficient evidence on its role in reducing child stunting |

| **POLICIES/STRATEGIES/PLANS** | | |
| --- | --- | --- |
| 1. Eighth Orientation Plan for Economic and Social Development/ Huitème Plan d’Orientation pour le Développement Économique et Social   (1989-1995) | Description | Starting in 1961, the Government of Senegal began issuing a series of consecutive 4 year plans known as the Quadrennial Economic and Social Development Plans (Plan Quadriennal de Développement Economique et Social, or PQDES). These plans were meant to define, outline and grow the central government’s policies for developing the country. The Eighth Orientation Plan for Economic and Social Development presents an assessment of the Nutritional Protection of Vulnerable Groups/Protection Nutritionelle des Groupes Vulnerable (PPNS) and an evaluation of sanitation conditions. Two programs included in the Eighth Plan were: i) the Rehabilitation and Surveillance of Nutrition Program; and ii) the Maternal and Infant Health and Family Planning Health Program (85,86). |
|  | Importance | Very important to understanding the country’s historic and current nutrition strategies and establishing nutrition as a national priority in development agendas |
| 1. National Plan of Action for Nutrition/Plan National d’Action Pour la Nutrition (PNAN)   (1997-2002)*  *PNAN was never actually implemented | Description | This plan was the result of international commitments made by Senegal during the International Conference on Nutrition and the World Declaration on Nutrition and following the aftermath of the devaluation of the CFA franc. This policy was Senegal’s first effort for multi-sectoral action to address nutrition through improving food security. It was comprised of a set of short-term and long-term strategies, actions and accompanying measures aimed at reversing the alarming trends in indicators of children's nutritional status. These included: an increase in agricultural production to meet the food needs of the population and address the balance between supply and demand, implement a cereal processing policy to improve the purchasing power of both rural and urban populations, and improve the coordination, efficiency, and management of an alert and information system during emergencies. This policy however was never implemented due to inadequate resources(86,87). |
|  | Importance | Likely important as Senegal’s first effort for cross-sectoral collaboration for nutrition |
| 1. National Health Development Plan/ Plan National de Developpement Sanitaire (PNDS)   (1998-2008) | Description | In 1998, the National Health Development Plan (PNDS) dissolved the existing National Program on Family Planning (PNPF) and transferred responsibilities out of the Departmental Cabinet of Health into the Division of Reproductive Health in the Department of Primary Health Care (88). The main objective of the PNDS was to improve the health of the entire population of Senegal, with a priority given to reducing maternal, infant and child mortality and decreasing fertility. It aims to respond to health needs of the population by focusing on equal access to quality care. Key priority areas include: human resources, strengthening of the health system, promotion of prevention, improvement of access to quality health services for poor/vulnerable populations, strengthening and integrating priority health programs (e.g. reproductive health). The PNDS was implemented in two phases including the Five-Year Integrated Health and Development Program (PDIS) from 1998-2002, and the reformed PNDS Phase II from 2004-2008 (89). |
|  | Importance | Very important as an effort to strengthen the health system |
| 1. Policy Letter for the Education and Training Sector/Lettre de Politique Generale pour le Secteur de l’Éducation et de la Formation   (2000-2015) | Description | The Policy Letter for the Education and Training Sector aimed to achieve universal education in order to meet the Millennium Development Goals and address poverty in Senegal. Within this policy letter, elementary education was seen as the main priority for development, and improvement of education quality and decentralization represented key strategies for this development. A focus on vocational and technical training was also included, and aimed to improve the labour market, developing a skilled workforce that could help grow the economy (90). |
|  | Importance | Likely important in improving the education system |
| 1. National Action Plan for Education for All/Plan National d’Actions de l’ Éducation Pour Tous (PNA EPT)   (2000-2015) | Description | Senegal hosted the World Forum on Education for All in Dakar in April 2000 where the Dakar Framework for Action was adopted. Later that year, the Ten-Year Education and Training Plan (PDEF) was introduced. The National Action Plan for Education for All (PNA EPT) aims to integrate and strengthen efforts of this Ten-Year Education and Training Program (PDEF). The PNA EFA represents a tool to achieve universal basic education and to implement Senegal’s 2001 constitution regarding the right to education. The Plan has numerous objectives: Firstly, to meet the fourth objective of the Dakar Framework of Action on Education for All by ensuring equitable access to adequate programs for young people and adults in order to acquire knowledge and skills needed in daily life. Secondly, re-adjusting schooling dimensions of girls and capitalizing on political will for early child development. Thirdly, strengthening focus on excluded and marginalized populations. Fourthly, emphasizing basic education by mobilizing resources for different education components and supporting resource allocation. Lastly, it sought to reinforce and pilot structures established in the PDEF. The plan was successful in developing a concept for early childhood development and the establishment of new structures for toddlers, as well as preschool classes in elementary and day care centres; improving the gross enrolment ratio for primary and secondary education; and increasing training for staff. However, spatial and gender disparities in access to basic education and insufficient education structures remain. |
|  | Importance | Likely important in reducing illiteracy rates |
| 1. Nutrition Development Policy Letter/Lettre de Politique de Développement de la Nutrition (LPDN)   (2001–2014) | Description | This policy brief on nutrition aligns with Senegal's commitments at the global level, notably within the Scaling Up Nutrition (SUN) initiative. The Nutrition Development Policy Letter (LPDN) reflects a new approach in the fight against malnutrition, highlighting the multi-factorial nature of its causes. The key objectives of the LPDN aimed to address: immediate causes of malnutrition related to inadequate dietary intake and the health status of vulnerable groups; underlying causes related to household food insecurity, the level of sanitation of the living environment, and the level of functionality of health facilities; root causes related not only to the level of socioeconomic development within Senegal but also to the institutional aspects; and promotion of a multi-sectoral approach in the implementation of nutrition interventions (91)**.** |
|  | Importance | Very important as the first effort to define national nutrition policy and to outline specific strategies for programs and monitoring |
| 1. Poverty Reduction Strategy Paper I (PRSP I)/Stratégie de Reduction de la Pauvreté (DSRP I)   (2001 – 2005) | Description | The goal of the Poverty Reduction Strategy Paper I (PRSP I) was to ensure adequate social, health and nutritional coverage for children from vulnerable families in Senegal. In its framework, the Government of Senegal was committed to developing and implementing a targeted nutritional policy for children living in the most vulnerable households. This included the establishment of cafeterias and latrines in schools located in poor areas, and awareness-raising activities geared towards parents, promoting oral rehydration therapy and nutritional interventions to prevent diarrheal diseases, drug addiction and growth delays (92). |
|  | Importance | Likely important as the country’s first multi-sectoral strategic movement towards addressing poverty |
| 1. National Free Delivery and Caesarean Policy   (2005-present) | Description | Similar to other such schemes in West Africa, the National Free Delivery and Caesarean Policy was created to prioritize select services for universal, free access. It aimed to reduce financial barriers for the use of public maternal health services, increase skilled attendance at birth, and reduce maternal/perinatal/neonatal mortality rates nationally. This policy was introduced in five of the poorest regions of country in January 2005, and in January 2006 extended to the remaining five regions (excluding Dakar). Implementation of this policy was focused at the regional hospital level. An evaluation of the policy found a significant increase in service utilization for normal deliveries (from 40% to 44%) and caesarean rates (from 4.2% to 5.6%) in target areas, though increases were not observed in national data (93). |
|  | Importance | Likely not important to the stunting decline but was one of first major moves towards free maternal healthcare in the country |
| 1. Strategic Plan for the Fortification of Foods in Senegal/Plan Stratégique pour la Fortification des Aliments en Micronutriments au Sénégal (PSFAMS)   (2006-2011) | Description | Following a meeting in Accra, Ghana in October 2002 organized by West African Health Organization, Micronutrient Initiative and Helen Keller International, recommendations were made for West African countries to develop a country-level fortification process. The Strategic Plan for the Fortification of Foods in Senegal (PSFAMS) aims to reduce the prevalence of micronutrient deficiencies (iron, vitamin A, iodine) in children under-5 years of age and women of reproductive age over a 5 year period. It aimed to reach these goals by taking the following actions: having the milling industries fortify 95% of wheat flour with iron and folic acid; having 100% of oil mills fortify edible oils with Vitamin A; and ensuring salt producers adequately iodize 90% of the salt consumed (94). |
|  | Importance | Likely not important to the major stunting decline from the mid-1990s to 2005 due to its time of initiation |
| 1. Poverty Reduction Strategy Paper II (PRSP II)/Document de Stratégie pour la Croissance et la Réduction de la Pauvreté (DSRP II)   (2006-2010) | Description | The overall objectives of the Poverty Reduction Strategy Paper II (PRSP II) focus on the achievement of the MDGs (e.g. reducing poverty by half by 2015); strengthening human capital; reducing vulnerability and inequalities; improving the quality of public services; promoting good economic and judicial governance; and increasing/speeding up economic growth (e.g. achieving an average economic growth rate of 7-8%). PRSP II pursues a multi-sectoral nutrition policy as reflected in the Nutrition Development Policy Letter. The nutrition-related objectives for this period include: halving the prevalence of malnutrition in children 0-5 years old; sustainably eradicating disorders related to iodine deficiency and Vitamin A deficiency; reducing by one third the prevalence of anemia (especially iron deficiency anemia); and ensuring the availability and sustainability in access to food of sufficient quantity and quality for the entire population. The nutritional component of the DSRP is supported in the continuity of the Nutrition Enhancement Program (PRN) (95). |
|  | Importance | Likely important as the country’s multi-sectoral strategic movement toward addressing poverty (continuation from earlier policy) |
| 1. National Policy on Infant and Young Child Feeding (IYCF)/Politique Nationale sur l'Alimentation du Nourrisson et Jeune Enfant (ANJE)   (2006-present) | Description | This policy is a result of WHO and UNICEF recommendations and the Global Strategy for Infant and Young Child Feeding. It aims to improve the nutritional status, growth, development and health of children and young people. It also aims to increase the rate of exclusive breastfeeding to at least 80% by 2015, achieve 95% coverage of Vitamin A supplementation and deworming of children 6-59 months of age, and reduce the prevalence of underweight children by 50%. This policy advocates the promotion of appropriate nutrition for infants and young children through: exclusive breastfeeding before 6 months; the introduction of complementary foods after 6 months; and fortification of complementary foods and nutrition supplementation (85). |
|  | Importance | Insufficient evidence of its role in the stunting decline |
| 1. National Strategic Plan for Child Survival/Plan National de Survie de l’Enfant (PNSE)   (2007-2015) | Description | The adoption of a National Strategic Plan for Child Survival was part of a context of technical and political re-mobilization to achieve the MDGs, based on short, medium and long term planning. In Senegal, this plan focuses primarily on the care of pregnant women, parturient and newborn infants; infant and young child feeding (including micronutrient supplementation and deworming); malaria prevention and treatment; provision and promotion of immunization services to mothers and children; prevention of mother-to-child transmission of HIV; medical care for children exposed to or infected with HIV; and care for common childhood illnesses. It aimed to achieve these by: improving the availability and accessibility of the integrated package of quality interventions for maternal, newborn and child health; increasing demand and use of services by populations, especially among vulnerable groups; and creating institutional, regulatory and economically favourable environments for scaling up the intervention package (96). |
|  | Importance | Likely not important to major stunting decline from mid 1990s to 2005 but relevant as a first major consolidated effort around MNCH and child survival |
| 1. National Health Development Plan (NHDP)/Plan National de Developpement Sanitaire (PNDS)   (2009-2018) | Description | This is a continuation of the 1998-2007 National Health Development Plan (PNDS). The implementation of this plan is in line with the achievement of national and international health goals, including the objectives of the Poverty Reduction Strategy Paper and the MDGs. It specifically aims to: reduce the burden of maternal, infant and child morbidity and mortality; increase the sector's performance in disease prevention and control; sustainably strengthen the health system; and improve the governance of the health sector. This plan also includes a nutrition component, notably through the attachment of the government’s Nutrition and Food Division to the Prevention Department (97). |
|  | Importance | Very important as the country’s current main policy on the health care system |
| 1. Scaling up Nutrition (SUN)   (2011 – present) | Description | Scaling Up Nutrition (SUN) is a global push for action and investment in order to improve maternal and child nutrition outcomes. Key strategic objectives include bringing people together, coherent policy and legal frameworks, aligning programs around a common results framework, financial tracking and resource mobilization. The main aim of SUN is engaging civil society organizations in advocating and sustaining political will for government action in scaling up nutrition. Senegal joined in 2011 and by 2017 has achieved 69% progress in the four indicators based on SUN scoring (98). |
|  | Importance | Promising recent initiative, however likely not important to the major stunting decline from mid 1990s to 2005 |
| 1. National Strategy for Social and Economic Development /Stratégie Nationale de Développement Economique et Social (SNDES)   (2013 – 2017) | Description | This policy builds on the Poverty Reduction Strategy Paper II and aims for sustained growth, increased productivity and the creation of wealth in Senegal. Specifically, it aims to support food security by strengthening the food crisis prevention and management system and the early warning system, and strengthening the information system on agricultural markets. The specific objectives for nutrition and health include: improving the supply of health services, improving performance in disease prevention and control, improving the health of mothers and children, and improving health governance (29). |
|  | Importance | Likely important as the country’s multi-sectoral strategic movement toward addressing poverty (continuing efforts of the PRSP I and II) |
| 1. Strategic Plan for Developing Universal Health Coverage in Senegal   (2013-2017) | Description | The Strategic Plan for Developing Universal Health Coverage in Senegal demonstrated substantial political will/commitment to Universal Health Coverage (UHC) following the election of President Sall in 2012. The proposed objectives focus on improved accessibility of health services among formal/rural sectors, protection against financial risks, and improved quality/satisfaction of services. Overall objectives of this plan include: equal access**;** financial protection of households**;** population satisfaction**;** expanded package of services provided/covered**;** governance and sustainability of UHC**;** equity in the health system and reduction of vulnerability through expansion of coverage (101). |
|  | Importance | Likely not important to stunting decline but is a promising recent initiative toward Universal Health Care |
| 1. Emerging Senegal Plan/Plan Senegal Emergent (PSE)   (2014-2035) | Description | The Emerging Senegal Plan (PSE) works to address the limited poverty reduction Senegal has experienced in recent decades. Three pillars will guide initiatives including: structural transformation of the economy through continued support to areas of growth, and development of new sectors to create wealth, jobs, and social inclusion; improvement in wellbeing of the population, and sustained efforts to combat social inequality; and finally strengthening security, stability, governance, the protection of rights and liberties, and a consolidation of the rule of law to create better conditions for social peace and fulfilment of potential (102). |
|  | Importance | Likely not important but promising recent initiative toward strengthening state governance and development |
| 1. National Community Health Policy/ Politique Nationale de Santé Communautaire   (2014-2018) | Description | This policy was initiated as part of the National Strategy for Social and Economic Development (SNDES). The main vision of this policy is participation of the Senegalese population to ensure a Senegal where all individuals, all households and all communities have universal access to health promotional, preventive and curative services. The three main objectives include: introducing a socio-sanitary, legislative and regulatory framework that emphasizes community health promotion; creating favourable conditions for the engagement and participation of all stakeholders in community health; and improving the planning process, monitoring and evaluation of the implementation of community health services. Key components of the policy focus on reinforcing community participation, capacity building of community actors, development of adapted service packages (including preventive and curative interventions), promoting multi-sectoral collaboration, increasing funding for community health, improved governance, motivating community health actors, and development of sustainability measures. (103). |
|  | Importance | Likely not important to the major stunting decline from the mid 1990s to 2005 but is a promising recent initiative |
| 1. National Community Health Strategic Plan /Plan Stratégique National de Santé Communautaire   (2014–2018) | Description | The National Community Health Strategic Plan aims to overcome challenges including poor health service coverage; inequality in access to care; insufficient harmonization of service packages; poor integration of community health in the overarching health system structure; a lack of community health actor motivation; and ineffective supply systems for essential medicines and products. The three main objectives of the plan are to improve coverage and quality of community health services; to strengthen community participation in problem-solving of health issues; and to ensure sustainability of community health interventions. This plan includes a list of community health services and interventions that are in the process of being integrated into one package to be delivered at community sites, health huts, and health posts. The list specifies the types of community health provider responsible for each intervention, organized by health area, including MNCH, reproductive health, family planning, disease prevention, nutrition, HIV and AIDS, TB, WASH, and neglected tropical diseases. The Community Health Program (PSSC) is one of the flagship programs under this strategy. (104,105). |
|  | Importance | Likely not important to the major stunting decline from mid 1990s to 2005 but is a promising recent initiative |
| 1. National Nutrition Development Policy Document /Document de Politique Nationale de Développement de la Nutrition (PNDP)   (2015-2025) | Description | This is a reform of the National Nutrition Letter. The general objective of this policy is to ensure a satisfactory nutritional state for all, particularly children under-5 years, women of childbearing age, and adolescents. Intermediate objectives include: ensuring adequate coverage in essential nutrition services for children under-5 years, women of reproductive age and adolescents; improving access and use of quality health services; improving population nutritional knowledge for the adoption of favourable nutrition behaviours; promoting research and production of high nutritional value food; obtaining sufficient and long-lasting financing of nutrition interventions; and strengthening the coordination, monitoring and evaluation of nutrition interventions as part of the multi-sectoral approach. These objectives are to be reached through the following four pillars: production of food with high nutritional value, improving availability, food security and nutrition; processing, distribution and pricing of food; education, hygiene and sanitation; and essential health and nutrition services (106). |
|  | Importance | Likely not important to stunting decline but it is a promising recent initiative around multi-sectoral nutrition planning |

| **PROGRAMS/PROJECTS** | | |
| --- | --- | --- |
| 1. Nutrition Protection Program of Vulnerable Groups /Programme de Protection Nutritionnelle des Groupes Vulnérables (PPNS)   (1973-1988) | Description | In response to the Sahel drought from 1968-1974, the Nutrition Protection Program of Vulnerable Groups (Programme de Protection Nutritionnelle des Groupes Vulnérables, or PPNS) was Senegal’s first large scale nutrition program. The main aim of the program was prenatal nutrition and growth, to be achieved through malnutrition prevention and nutritional recovery via supplementation. Food assistance as part of this program was to be provided by Catholic Relief Services (CRS), complemented by the Government’s Health/Drought (Santé/Sécheresse) program, supported by WFP. Key components of this program included the delivery of nutrition services through health posts. Among these services were nutritional rehabilitation; distribution of food to malnourished children and pregnant and lactating women; pre- and post-natal consultations; growth monitoring of children under-5, and nutrition education (85,86). |
|  | Importance | Not important because it is an early (though long-standing) food supplementation program that had many deficiencies |
| 1. Expanded Program on Immunization (EPI)   (1979-present) | Description | The main objective of this program is to ensure full immunization of children against preventable diseases, especially among children 0-15 months of age. In 1979, the Expanded Program on Immunization (EPI) program vaccinated against seven diseases. Additional vaccines were added over time, including polio (1988), yellow fever (1988), pentavalent vaccine (diphtheria, tetanus, pertussis, haemophilius influenza b and Hepatitis B) (2005), pneumococcus conjugated vaccine (2013), measles & rubella (2014), rotavirus (2014), inactivated polio vaccine (2015), and hepatitis B (2016). Despite the substantial costs to fund the program, achieving high coverage rates continues to represent a challenge in Senegal. In 2010, immunization coverage dropped to 70% in some districts (with some areas as low as 50%) (107). |
|  | Importance | Likely important to stunting decline due to its role in vaccinating children |
| 1. Community Nutrition Project /Projet de Nutrition Communautaire (PNC)   (1995 – 2001) | Description | The Community Nutrition Project (PNC) was created in response to the alarming nutritional situation in Senegal, which was seen to result from the cumulative negative effects of climate hazards, structural adjustment, the global economic crisis and the devaluation of the CFA franc. The three main objectives of this project included: (i) halting further deterioration in the nutritional status of children under-3 years of age, as well as pregnant and lactating women in targeted poor urban neighbourhoods; (ii) providing portable water to under-serviced neighbourhoods targeted under the nutrition programme; and iii) enhancing food security among the poor urban population and in targeted poor rural areas during critical periods of vulnerability. This program focused on the most vulnerable groups in the impoverished population, including districts within the country that had high rates of poor households. A total of 1.2 million people were targeted to benefit from this project. This included an estimated total population of 469,000 receiving nutritional interventions, such as 230,000 malnourished children under-3 years and 120,000 nursing and pregnant women receiving food , growth-monitoring and IEC services; and 119,000 mothers and children receiving only growth-monitoring and IEC services (108). |
|  | Importance | Very important to stunting decline as an early & effective effort for consolidated community nutrition targeting |
| 1. Water Sector Project/ Projet du Secteur de l’eau   (1996-2004) | Description | As part of an overarching movement to reform and improve management of the water supply sector in Senegal, this program was the first of three planned initiatives aimed at increasing coverage and organization of water and sanitation. The overall objectives of this first program included achieving: i) sustainability through improvements in management, pricing, and cost recovery and a reduction of the Government subsidy for industrial, domestic, and irrigation water; ii) poverty alleviation and improved health through increased access to safe potable water and adequate/more affordable sanitation for the urban poor; and iii) private sector participation, by engaging a private company to manage urban water supply. Overall, World Bank donor reporting indicates that this project was seen as a success, and Senegal was praised as a model for the implementation of public-private partnerships in sub-Saharan Africa (109–112). |
|  | Importance | Likely important, WASH indicators improved modestly and reduction of open defecation was important to child stunting gains |
| 1. Vitamin A Supplementation Program   (1999-present) | Description | The main objective of this program is to deliver a high-dose of Vitamin A to children aged 6-59 months to reduce mortality and morbidity. Through Child Survival Days, a package of interventions is offered in Senegal twice annually, including: deworming, growth monitoring, screening and referral for severe malnutrition, screening and treatment for diarrhea, catch-up for vaccinations and civil registration for children under-5. Vitamin A supplementation coverage in the majority of Senegal is high with 98% coverage across 9 out of 14 regions in December 2015. In the remaining 5 regions using routine delivery in that same year, coverage was only 54% (113). |
|  | Importance | Likely important but insufficient evidence on scale and impact |
| 1. Ten-Year Education and Training Program/Programme Décennal d'Education et de Formation (PDEF)   (2000-2010) | Description | Ten-Year Education and Training Program (PDEF) resulted from several international gatherings related to education, including the 1990 Jomtien Education Conference and the 2000 World Conference on Education for All in Senegal’s capital Dakar. The main objective of the program is educational reform. Specific goals aim to increase access to universal quality education and training by 2010; introduce a relevant and high-quality education system at all levels; create a coordinating body for educational policies and programs; and rationalize mobilization and use of resources. Substantial achievements after just three years (as of 2004) were noted including achievements in standardized results for French/math, construction of classrooms, introduction of a minimum number of teaching hours (20 hours), improvements in the ratio of textbooks to students, and increased educational enrolment (114,115). |
|  | Importance | Likely important |
| 1. Coordination Unit for the Fight Against Malnutrition/Cellule de Lutte Contre la Malnutrition (CLM)   (2001-Present) | Description | The Coordination Unit for the Fight Against Malnutrition (CLM) represents a coordinating body for nutrition in Senegal, housed within the Prime Minister’s Office. The main objectives of the CLM include: providing technical assistance in defining and implementing national nutrition policy; examining and approving proposals for collaboration by ministries in the execution of the program; facilitating a consultative framework between technical ministries, stakeholders in charge of nutrition policies, NGOs, and community-based organizations; developing good synergy with other anti-poverty programs; promoting a policy of Behavior Change Communication and good practices in the fight against malnutrition; and supporting national capacity building for effective implementation of nutrition programs. The CLM has led to the coordination and engagement of multiple sectors including both nutrition-specific and –sensitive areas, resulting in increases in funding for nutrition**.** Current projects and programs under the CLM include: Nutrition Enhancement Program (PRN); Universal Iodization of Salt Project (PIUS); Fortification Enhancement Program in Senegal (PRF); Food Security Support Project for Vulnerable Households (PASAV); Demand-based financing component of the Health and Nutrition Financing Project (PFSN); Fight Against the Determinants of Malnutrition Project (PLDM) in the North-East of Senegal; a results-based financing project targeting demand for maternal care; and a project to improve the response of most vulnerable communities to nutritional and food crises in the areas of Podor, Ranerou, Matam and Kanel (116). |
|  | Importance | Very important as the coordinating body overseeing the PRN and other nutrition-sensitive initiatives |
| 1. Nutrition Enhancement Programme /Programme de Renforcement de la Nutrition (PRN I)   (2002-2006) | Description | Building on lessons learned from the Community Nutrition Project (PNC) which ran from 1995-2001, the Nutrition Enhancement Programme (PRN) focused on a commitment to improve nutrition in Senegal. It is a large-scale national program ran by the CLM. The two objectives of the PRN include: improving the nutritional status of populations, especially the growth of children under 3 years of age living in poor urban or rural areas; and strengthening the country's institutional and organizational capacity for nutrition for the implementation and evaluation of nutrition policy. It included the following community–based nutrition and growth promotion activities: monitoring and promotion of growth, with monthly weighing sessions and home visits to vulnerable children (who did not participate or showed insufficient weight gain); nutrition and health group education for dissemination of key messages; IMCI, including the promotion of healthy behaviors and the design and implementation of guidelines for the management of severe cases of malnutrition in health facilities; provision of basic health services, including basic commodities (micronutrients, deworming tablets, insecticide-treated mosquito nets and Oral Rehydration Salts) and promotion of antenatal care and other health services; and grants for community nutrition projects. Other components include capacity building and monitoring and evaluation, as well as project management and reporting (117,118). |
|  | Importance | Very important as the main national nutrition program that used a multi-sectoral approach |
| 1. Long-Term Water Supply Project/Projet Eau à Long Terme (PLT)   (2002-2005) | Description | The Long-Term Water Supply Project (PLT) was part of an overarching movement to reform and improve management of the water supply sector in Senegal. This was the second of three planned initiatives. This project aims to make sustainable improvements to the delivery of water and sanitation services in urban areas within Senegal, including under-served/low income areas of Dakar and other cities. In the areas covered by the project, 1,415,000 people benefited from access to drinking water (725,000 in Dakar and 690,000 in other urban centers of in the interior). This represented 141% of the initial target of 1 million people with access.  Access to sanitation reached 103% of the target (144,500 people connected from an initial target of 140,000), and 146% achieved for the target of autonomous and semi-collective sanitation (583 000 people served from an original goal of 400,000) (119–123). |
|  | Importance | Likely important, WASH indicators improved modestly and reduction of open defecation was important to child stunting gains |
| 1. Water and Sanitation Millennium Program/Programme d’Eau Potable et d’Assainissement du Millénaire (PEPAM)   (2005-2015) | Description | The Water and Sanitation Millennium Program (PEPAM) was part of an overall plan to reform water and sanitation services in Senegal. It was the third of a set of three initiatives in this reform effort, following the Water Sector Program (1996-2004) and Long-Term Water Supply Project (2002-2005). It was the means by which the Government worked to achieve the MDGs related to water and sanitation, creating a global framework to coordinate interventions in the sector. The main objective was to increase access to sustainable water and sanitation services in selected rural and urban areas within Senegal. The key components were: improving rural water supply (i.e. access to drinking water in the regions of Saint-Louis and Matam, and the district of Bakel in the Senegal River basin); improving urban water supply; improving rural sanitation by increasing access to water sanitation services in the regions of Saint-Louis and Matam and the district of Bakel; improving urban sanitation; and institutional strengthening and capacity building by supporting activities associated with ongoing water sector reforms. According to the World Bank, this project was seen to be highly effective. This was due to factors including strong design and implementation, achievement or exceeding of set targets; and high efficiency within the program (124,125). |
|  | Importance | Likely important, WASH indicators improved modestly and reduction of open defecation was important to child stunting gains |
| 1. National Program for Local Development/Programme National de Développement Local (PNDL)   (2007-2012) | Description | The purpose of the National Program for Local Development (PNDL) is to effectively, efficiently and sustainably promote the provision of basic services to the Senegalese population. It aims to set up a framework for participatory local government, decentralization, resource mobilization/transfer to local governments and communities in order for more effective service delivery in Senegal’s rural and poorest areas. The objectives are to: increase access to basic social infrastructure and services through direct and indirect interventions by at least 50% over 5 years; increase the access of the poorest populations to financial resources for the development of income-generating activities, with the goal of at least a 25% improvement of the incomes of 75% of the poorest households within 5 years; strengthen the capacity of local actors, with at least 75% of the targeted local actors implementing their reinforcement plans at 70% after 5 years; and strengthen the State’s capacity to implement the Poverty Reduction Strategy (126,127). |
|  | Importance | Likely important as further effort for the decentralization of social services including health |
| 1. Nutrition Enhancement Programme II Programme de Renforcement de la Nutrition (PRN II)   (2007-2014) | Description | The Nutrition Enhancement Programme II (PRN II) is a continuation of the first phase of the program, the PRN I. The objectives remain the same as the PRN I: to improve the nutritional status of the population, especially children under-5 years of age living in poor urban or rural areas; and to strengthen the country's institutional and organizational capacity for nutrition in the implementation and evaluation of nutrition policy. Program coverage was increased and additional project activities were scaled up to continue the work towards the objectives of the programme (117,128,129). |
|  | Importance | Very important as the current main national nutrition program that uses a multi-sectoral approach |
| 1. Rapid Response Child-Focused Nutrition and Social Transfers Project/ Programme de Nutrition Ciblée sur l’Enfant et Transferts Sociaux (NETS)   (2009-2011) | Description | This program provides benefits to mothers of children ages 0-5 years in poor areas of Senegal, in order to help offset the cost of rising food prices. The main objectives of NETS include: reducing the risk of nutritional insecurity for vulnerable populations by intensifying the Nutrition Enhancement Programme (e.g. community nutrition monitoring, promotion activities and other services leading to improved nutrition knowledge that will lead to changes in dietary practices and behaviors); and reducing the risk of nutritional insecurity for vulnerable populations by transferring cash to mothers of vulnerable children under-5, thereby improving their ability to access essential food and other investments for the well-being of their children. The program involved 50,000 mothers of children aged 0-5 living in a poor households (130). |
|  | Importance | Likely not important to long-term stunting decline, but promising first-effort towards using cash/social transfer methods to target at risk populations (women and children) |
| 1. Child Nutrition and Food Security Project/Projet Nutrition Enfant et Sécurité Alimentaire (NESA)   (2009-2012) | Description | Child Nutrition and Food Security Project (NESA) was created following Senegal’s experience with droughts in 2006 and 2007, as well as rising food prices. The main objectives of this project included: reinforcement of the capacities of local communities (especially vulnerable groups) as part of the fight against child malnutrition; implementation of the Integrated Management of Childhood Illness (IMCI) Program and Community and Acute Malnutrition Management in all target districts; reinforcement of Behavior Change Communication programming related to health, and improvement of the early warning system. This project was implemented in seven of the regions in Senegal most affected by the food crisis, including Tambacounda, Kolda, Matam, Diourbel and Louga, Kedougou and Sedhiou. It targeted a total population of 4,300,000 inhabitants, including: 817,000 children under-5 years old; and pregnant and lactating women (131,132). |
|  | Importance | Likely not important to long-term stunting decline, but effective effort towards alleviating burden from short-term food shocks due to 2006/2007 drought |
| 1. Bajenu Gox Project   (2009-Present) | Description | This initiative was launched in 2009 by former Senegalese President Abdoulye Wade in order to combat the country’s high maternal and child morbidity and mortality rates. The Bajenu Gox Initiative, whose name is derived from the term « godmother » in Senegal’s Wolof language, works to train women to be leaders in reproductive health, supporting other women in the pre-natal, delivery and post-natal periods, as well as providing advice on care of children under-5. Bajenu Gox provide counselling services, home visits, and lead women’s groups. They consult with women about utilizing health centres; giving birth in health facilities; the importance of attending pre-natal check ups; vaccinating their children; and family planning/birth spacing. According to the Government of Senegal, the Bajenu Gox initiative has contributed to reducing maternal deaths from 410 to 315 per 100,000 between 2008 and 2015, while infant deaths fell from 59 to 50 per 1,000 during the same time period (105,133,134)**.** |
|  | Importance | Likely not important to long-term stunting decline, but promising efforts towards expanding community-based health care within existing system |
| 1. Universal Salt Iodization project (PIUS)/Projet d’Iodation du Sel   (2009-2014) | Description | To fight against iodine deficiency disorders, the Government of Senegal adopted the universal strategy of salt iodization which was led by the CLM beginning in 2006. However, following a 2010 national survey showed relatively low levels (47.7%) of appropriately iodized salt (≥15ppm) consumption in Senegal, renewed efforts were made by the CLM with support from Nutrition International, the Global Alliance for Improved Nutrition (GAIN) and UNICEF. This project has the goal of a sustainable elimination of iodine deficiency disorders, with the basic strategy of universal iodization of salt for human and animal consumption. Specific aims include: achieving the goal of 90% of households consuming iodized salt and increasing the annual production of iodized salt by small producers by 80,000 tonnes. It plans to reach these goals by targeting small salt producers (250,000 to 200,000), strengthening political commitment for salt iodization, supporting the management activities of artisanal salt farmers, and ensuring a functional monitoring system to guide the action of stakeholders (135,136). |
|  | Importance | Likely not important to stunting decline |
| 1. Food Fortification Enhancement Program/Programme de Renforcement de la Fortification des Aliments   (2010-2015) | Description | The CLM launched the Food Fortification Enhancement Program to combat micronutrient deficiencies. The main objective of this program is the fortification of all wheat, oil and flour consumed in Senegal in order to reduce micronutrient deficiencies within the population (137). |
|  | Importance | Likely not important to stunting decline, but promising recent initiative around fortification |
| 1. USAID/Yaajeende Agriculture and Nutrition Development Program for Food Security/ Programme de Développement Agricole - Sécurité Alimentaire USAID-Yaajeende   (2010-2017) | Description | This project is part of Feed the Future/USAID's new global strategy against hunger and food insecurity, which aims to work with host governments and development partners to address the root causes of hunger and poverty in the world. This project aligns with the National Health Strategy Plan and the National Agricultural Investment Plan. It aims to engage in a country-led integrated approach to accelerate the participation of 500,000 of Senegal’s rural poor in growth, and to address malnutrition in children under-5 by reducing the rate of underweight and stunted children by 30% each. Project activities are divided into five major areas: increasing the availability of food by improving the diversity and sustainability of agricultural production and by promoting sustainable land management; increasing and diversifying revenues from agriculture by stimulating key agricultural markets and value chains; reducing undernutrition and ensuring a healthy diet through improved utilization of foods; improving capacity for local governance of food-related resources; and cross-cutting activities. The midterm evaluation shows that USAID/Yaajeende has been very successful at reaching its target groups and beneficiaries, i.e. the poor and the vulnerable, especially women but did not evaluate sustainability (138). |
|  | Importance | Likely not important to long-term stunting decline, but effective donor funded project targeting the most at-risk populations under-5 in rural communities of select regions |
| 1. National Agricultural Investment Program /Programme Nationale d’Investissement Agricole (PNIA)   (2011-2015) | Description | Senegal developed National Agricultural Investment Program (PNIA) to translate into action commitments made under the Economic Community of West African States (ECOWAS) Regional Agricultural Policy for West Africa (ECOWAP), and the Comprehensive Africa Agriculture Development Program (CAADP). It also aligns with Senegal’s current policy and strategy documents for agriculture. The PNIA aims to ensure modern, sustainable, productive and competitive agriculture in intra-community or international markets, based on the efficiency of family farms and the promotion of agricultural enterprises through involvement of the private sector. Its key components include reducing climate risk through water management; preserving and sustainably managing other natural resources; boosting production and enhancing natural resources; increasing production and greatly improving overall factor productivity; adding value to agricultural produce through processing; improving marketing of agricultural produce through better market access; strengthening research to generate and transfer new technologies in production, processing and marketing; significantly strengthening the capacities of the various stakeholders; and ensuring effective coordination and steering of investment program implementation (139,140). |
|  | Importance | Likely not important to stunting decline from mid 1990s to 2005 but is a promising recent initiative |
| Integrated Education and Nutrition Program/Programme Integre Sante Education Nutrition (PISEN)  (2012-2016) | Description | The Integrated Education and Nutrition Program (PISEN) aims to improve the health and wellbeing of the people of Senegal, with a specific focus on women, children and vulnerable groups. The key goals/outcomes for this program are to: improve the food and nutrition security of populations (both men and women) in a sustainable manner; ensure children, adolescents, adults and the most vulnerable populations receive basic education and quality training; and ensure that within intervention areas, mothers, children, and the most vulnerable benefit from a comprehensive package of quality health interventions. Services are delivered in four regions: Sedhiou, Kolda, Kedougou & Dakar (141). |
|  | Importance | Likely not important to long-term stunting decline, but promising multilateral donor-funded project targeting the most at-risk women and children in select regions |
| 1. The National Family Safety Scholarship Program (PNBSF)/ Programme Nationale de Bourses de Sécurité Familiale (PNBSF)   (2013-2019) | Description | The National Family Safety Scholarship Program (PNBSF) aims to contribute to the fight against poverty and social exclusion of the poor and vulnerable in Senegal. It provides conditional cash transfers for using education and health services, working to strengthen the development of human capital in children in order to stop the intergenerational transmission of poverty. The program has three main focus activities/areas: registering and maintaining children in school; immunization cards for children 0-5 years old; and registering children at the registry office. The total number of beneficiary households that have received cash transfers since the program began in 2013 is close to 200,000 households (50,000 households in 2013, 50,000 households in 2014 and 100,000 households in 2015). An evaluation in 2018 indicated that it had reached up to 300,000 (142,143). |
|  | Importance | Likely not important to long-term stunting decline, but promising first efforts at national social safety program targeting at-risk families in the entire country |
| 1. Community Health Program (PSSC)   (2016-2021) | Description | The Community Health Program (PSSC) is one of the flagship programs of the National Community Health Strategic Plan. It is a reform of the Senegal Health Project First Phase (2006-2011) and Senegal Health Project Second Phase (2011-2016). The previous projects focused on implementing a package of integrated services to impact the public health of the Senegalese population, focusing on family planning/reproductive health, maternal/child health, nutrition, malaria, TB, and HIV/AIDS (awareness) at community-based health huts. This new phase of the Community Health Program is focused on continuing to provide the package of services from Phases 1 and 2, but to a larger geographic area and with greater emphasis on sustainability. This component is working in all 14 regions and 72 health districts. It’s objectives are to improve the health status of Senegal’s population, make strategic investments to build country capacity, and have a sustainable impact on maternal, neonatal, and child mortality and other public health priorities (105,144). |
|  | Importance | Likely not important but promising initiative for strengthening community-based healthcare |
| 1. Multisectoral Strategic Plan for Nutrition in Senegal/Plan Strategique Multisectoriel de la Nutrition du Senegal (PSMN)   (2017-2021) | Description | The Multisectoral Strategic Plan for Nutrition in Senegal (PSMN) aims to operationalize the National Nutrition Development Plan (PNDN), which is focused on ensuring that Senegal is a country where every individual has an optimal nutritional status through the adoption of appropriate behaviours. It also aims to provide a situational analysis of nutrition in Senegal over the last five years and analyze key determinants of malnutrition, including regional disparities and contributions from diverse sectors. It would like to highlight key achievements and challenges for nutrition in Senegal and outline the vision towards 2025 and strategic objectives for nutrition. It contains five main axes: preventing malnutrition and non-communicable diseases related to nutrition; fight against micronutrient deficiencies (iron, folic acid, iodine, zinc and vitamin A); strengthen the availability and accessibility of diverse food with high nutritional values; research and innovation; and governance of nutrition(145). |
|  | Importance | Recent promising initiative for multi-sectoral nutrition action |

# **Supplementary Appendix 7:** Qualitative Results

**Qualitative Inquiry – full results**

Results from the in-depth interviews and focus group discussions are organized according to type of stakeholder, by national and community-level perspectives. Results are summarized according to key themes including basic (contextual) factors, nutrition-specific and –sensitive policies and programs, intermediate and immediate causes. Supporting evidence and quotes were selected to demonstrate a range of participants’ diverse perspectives on the determinants, and policies and programs.

**National Stakeholder Perspectives**

In-depth interviews were conducted with 21 national key informants and stakeholders working in the health and nutrition sectors (**Supplementary Appendix** **Table 10A**). These State and non-State actors were identified and recruited based on their substantial expertise and experience working in nutrition-specific and –sensitive sectors, institutions and programs in Senegal and include multilateral, bilateral, government (national and subnational), academic/research, as well as local/international non-governmental organizations. Key informants’ responses were categorized into several major drivers based on our conceptual framework including basic or distal causes, nutrition-specific or sensitive programs, intermediate causes, and immediate or proximate causes.

## **Supplementary Appendix Table 10A**: Summary of national stakeholders

| **Participant #** | **Organization** |
| --- | --- |
| Participant 1 | Monitoring & Evaluation Officer, Cellule de Lutte Contre la Malnutrition, CLM (Unit Fighting Against Malnutrition) |
| Participant 2 | Institute of Social Pediatrics |
| Participant 3 | Direction of Maternal and Child Health, Ministry of Health |
| Participant 4 | UNICEF |
| Participant 5 | WHO |
| Participant 6 | Retired Professor (Pediatrics & Nutritionist) |
| Participant 7 | Professor of Pediatrics, Faculty of Medicine |
| Participant 8 | Head of Nutrition Division, Direction of Maternal and Child Health, Ministry of Health |
| Participant 9 | Medical Doctor, Cellule de Lutte contre la Malnutrition, CLM (Unit Fighting Against Malnutrition) |
| Participant 10 | Sociologist, Hellen Keller International |
| Participant 11 | Nutritionist, Hellen Keller International |
| Participant 12 | Nutritionist, Hellen Keller International |
| Participant 13 | Medical Doctor, Former Director of Hellen Keller International |
| Participant 14 | Nutritionist, Intrahealth, Neema/ Intrahealth-Nema |
| Participant 15 | Intrahealth-Neema |
| Participant 16 | Nutrition International (Former Micronutrient Initiative) |
| Participant 17 | Action against Hunger (ACF) |
| Participant 18 | Institute of Food Technology (ITA) |
| Participant 19 | Food Security Executive Secretariat |
| Participant 20 | Director, Ministry of Woman, Family and Gender |
| Participant 21 | Social protection and national solidarity Agency |

***Contextual Factors***

Key contextual factors that facilitated gains achieved in the reduction of stunting include political stability and peaceful democratization, improved education/women’s empowerment, urbanization, as well as moderate reduction of poverty and increased accumulation of wealth.

*Social Political Context*

The 1990-2000s period featured low economic and social conditions, with the majority of the population living below the poverty line. Further, in 1994, structural adjustment reforms resulted in the rapid devaluation of the CFA franc by the IMF, as well as inflation, decreased commodity (particularly agricultural/crop) prices, increased food insecurity and civil unrest in largely the urban regions, including Zinguinchor (86). Low-level internal conflict in the Casamance region (southwestern Senegal composed of Zinguinchor and Kolda) has been ongoing since 1982 and was initiated by the separatist *Mouvement des forces democratiques de la Casamance* (MFDC). An estimated 3,000-5,000 people have died during the conflict and the number of internally displaced persons during the peak of insurgency, during late 1980s, was estimated at over 60,000 individuals, largely from the Zinguichor region (146,147). Despite this longstanding internal conflict, key informants highlighted that low violence and general political stability have represented driving forces and provided a supportive policy environment for public investment in education, health and nutrition.

*"Senegal has not experienced any major crises, war, or unrest, which has kept the country calm despite the political upheavals that have never prevented the country from functioning. The institutions are in place and fully operating and people live well; there is nothing comparable to a country in war. So when we have an environment of peace, of political stability in my opinion, the social indicators improve, children will go more to the schools, with the opening of the health structures, the populations have better access to healthcare, and with the efforts in the field of hydraulics, people have better access to drinking water and sanitation. These are advances that have led Senegal to huge improvements in nutritional status.”* [Former Country Director of Hellen Keller International]

*Poverty Reduction*

Poverty has reduced somewhat in Senegal between 1991 and 2011, as the poverty headcount ratio at $1.90 a day was almost halved from 68% to 38%, respectively (1). The Multidimensional Poverty Index (MPI) in Senegal has also shown some improvement over time as it decreased from 0.440 in 2010 to 0.293 in 2017. However, substantial inequities in the MPI exist between rural (0.434) and urban (0.119) areas (148,149). National respondents indicated that high rates of poverty contributed to the initial burden of stunting, and that declines in poverty and improved standards of living were related to observed decreased chronic child malnutrition. Experts felt that poverty reduction was largely a result of investments by the State through initiatives, including the PNC and PRN, with integrated elements of social protection to support resilience during the economic challenges related to the currency devaluation.

“*As you can see over the years there is some progress in some factors that actually...that actually impacted the level of stunting. Because there is a small evolution in the levels of knowledge; it has not remained at the same level because the level of education of the populations is increasing over time. The standard of living of some populations has also contributed in slightly affecting stunting. Indeed, a thorough analysis of stunting indicates that it is higher in the quintiles with the highest levels of poverty.”*[UNICEF Representative]

*Education & Women’s Empowerment*

Overall, net enrolment in primary school has increased from 49.2% in 1996 to 72.3% in 2016 (1). Disparities in education by gender have decreased, as the gender parity index of primary school comparing enrolment of girls and boys has increased gradually between 1971 and 2016 from 0.594 to 1.08, respectively. In addition, female literacy (aged 15-24 years) has increased from 41% in 2002 to 59% in 2011, however a decline to 51% in 2013 was observed (1). Further, the Gender Development Index (GDI) increased from 0.781 in 1995 to 0.911 in 2017, and a slight improvement in the Gender Inequality Index (GII) was achieved over the same time period from 0.644 to 0.515 (150). The proportion of seats held by women in the national parliament has increased dramatically since the late 1990s, from 11.7% in 1997 to 41.8% in 2017 (1), achieving substantial progress towards achieving gender parity in national decision making structures. Increased overall educational attainment and improved literacy were recognized by national respondents as driving factors in improvements in nutrition. In particular, targeted efforts in the education sector focusing on girls’ education has led to a significant increase in the proportion of girls in the school population. Programs that focused on health and nutrition promotion also contributed to an increased knowledge among women relating to nutrition.

“*The higher the level of education, the easier it is for the person to become aware, the more quickly they adopt the practice, according to what is noticed on the field. Accompanying women who have a fairly high level of education are much easier to raise awareness with whom it is easier to negotiate with the desired practices.*” [USAID Representative]

*“And so this [PISEN] is a program that has really helped to educate women nutritionally, to teach them how to transform certain foods, what foods are rich, what foods can be put together to enrich the diet and especially they have been taught to fortify food.”* [WHO Representative]

*Urbanization*

Senegal has experienced a consistent increase in urbanization over time, from 39.2% of total population living in urban areas in 1992 to 46.7% in 2017 (1). Despite this moderate increase in urbanization over time, this did not represent a key driver of stunting decline according to national respondents. Despite this moderate increase in urbanization over time, no national respondents identified urbanization as a key driver of stunting decline in Senegal.

*Remittances & Labour Migration*

Remittance inflows due to labour migration increased from $142 million USD in 1990 to $2,238 million USD in 2017 (151). Further, the inflows as a proportion of GDP have gradually increased over time from 2.5% of GDP in 1990 to 13.7% in 2017 (152). Key destinations for international migration from Senegal included France, Gambia, Italy, Spain and Mauritania (153), with the majority migrating for labour and employment opportunities (154). More than half of remittances received in Senegal are spent on food, education and health care (human capital) (155). Further, according to the Senegal 2009 Migration and Remittances Household Survey, households spent over 80% of remittances on food (156). Despite these trends, remittances and labour migration were not discussed by any national and community-level respondents as driving factors of nutrition or stunting improvements in Senegal.

**Basic Causes: Intermediate I**

National respondents indicated that several nutrition-specific and –sensitive policy efforts demonstrated political will and commitment towards improving nutrition in the country. Challenges faced during the implementation of initiatives were also outlined.

***Nutrition-Specific Policies & Programs***

Significant political will and commitment by the Government of Senegal to global targets (e.g., the MDGs), was identified by several national key informants as a critical driver to improvements in nutrition and the reduction of stunting.

“*We must start by saying that these are political commitments that Senegal has made in relation to charters at the global level. At the global level too, there have been commitments that states have implemented to fight against malnutrition and all forms of malnutrition because people knew that it is this malnutrition that will lead us to adult chronic diseases. And so, with all these commitments that Senegal has ratified and to respond to all this at the political and hierarchical level, some policy documents and strategic plans have been developed, and a cell has been set up to centralize all the sectors of development for a better inclusion of nutrition.”* [WHO representative]

*Community Nutrition Project (PNC, Project de Nutrition Communautaire)*

The PNC (1995-2002) was a pivotal initiative to address nutritional challenges in Senegal due to environmental conditions, as well as poor socioeconomic climate and poverty due to the structural adjustments and devaluation of the FCFA (85). Funded by the World Bank and implemented by the Agency for Public Works and Employment (AGETIP), this program targeted poor urban areas (86) and was piloted for 6 months in three cities and subsequently implemented in almost all urban areas nationally over five years (157). Key components of the PNC included a focus on information, education and communication, growth monitoring of children under-3 years, provision of food supplements, referrals for health services and home visits for participating children. Improved drinking water and food security were also targeted through the PNC (85). Despite substantial challenges to implementation including high costs, inadequate targeting of efforts and capacity building of stakeholders, as well as a lack of coordination and ownership of nutrition by the Ministry of Health, the PNC represented a catalytic initiative in the nutrition sector (86). The adoption of a community-based and multi-sectoral approach to address causes of stunting at grassroots level and moving away from a more curative approach was outlined by national respondents as a key contribution of the program. Further, the PNC was a critical effort to catalyze the prioritization of nutrition at the national level, and contributed substantially to the initiation to the establishment of the CLM.

*"An approach where one really had to go to the community level, work with the community, to change nutrition behaviours. This is the difference with the PNC that was [not] just about distributing flour to the mother and child. It was much more about a development approach that involves working with communities to reduce stunting, developing knowledge and changing behaviors. There was a need for a multi-sectoral approach.”* [UNICEF Representative]

*"So when there was this community nutrition program; in 2000 this program was evaluated. Now we thought, after the evaluation of the program, we now have to move forwards for nutrition. This is how the CLM was created. And when the CLM was created the Nutrition Enhancement Program came ..."* [UNICEF Representative]

*Nutrition Coordinating Body: Cellulue de Lutte Contre la Malnutrition (CLM)*

In 1994, the *National Committee for the Fight Against Malnutrition* (*Commission Nationale de Lutte contre la Malnutrition, CNLM*) was initiated by a Presidential decree, largely to provide oversight for the implementation of the PNC (86). In 2001, the *Cellule de Lutte contre la Malnutrition* (CLM) was established (and replaced the CNLM) with the support of development partners, including the World Bank, USAID, UNICEF and WHO. The CLM represents a coordinating body for nutrition, and has been instrumental in catalyzing increased political will and prioritization of nutrition by decision-makers over time, including increased financial allocation of resources (86). Key programs led by the CLM include the PRN, Universal Salt Iodization Project, Fortification Enhancement Program (PRF), Food Security Support Project for Vulnerable Households (PASAV), demand-based financing component of the health and nutrition financing project (PFSN), results-based financing project for maternal care, and projects focused on nutritional/food crises in Podor, Ranerou, Matam and Kanel (132). The CLM has substantially contributed to the institutionalization of nutrition through the establishment of a framework and implementation of efforts across all regions of the country. The positioning of the CLM in the Prime Minister’s office, its concerted effort to coordinate nutrition across multiple sectors, increased financial resource allocation and prioritization of nutrition and the revision of the national nutrition policy represented critical achievements by the CLM to improve nutrition and stunting in Senegal. Several national respondents felt that the establishment of the CLM represented a substantial contribution towards ensuring nutrition represented a priority across sectors and levels.

“*...The government set up since 200[1] a structure to promote a multi-sectoral approach in the fight against malnutrition. This unit brings together all the technical ministries that have a role to play in the fight against the determinants of malnutrition. This structure is highly set up at the level of the Prime Minister's Office, which facilitates the coordination of sectoral interventions. So the state of Senegal has [from] very early [on] understood the advantage of this multi-sectoral approach to the fight against malnutrition, as each sector uses its comparative advantages to identify interventions to combat malnutrition. The other important element for me is that the State of Senegal has dedicated an important budget line to nutrition. And so the state regularly increases its share in the fight against malnutrition within their budget. Partners also support the state in its efforts to fight against malnutrition. I think these are very important elements, but additionally the Government recently revised its nutrition development national policy letter to involve more the other sectors, in particular the private sector in the fight against malnutrition through food enrichment and fortification of food; the government has also enhanced hydraulics, sanitation, and fishing to increase the availability of access to drinking water and the availability of aquatic products to fight against micronutrient deficiencies.”* [CLM representative]

*“I think it's mostly multisectoral policy! I think that the CLM, the community actors contributed a lot to that.”* [Institute of Social Pediatrics]

*“Now it is in 2001 with the creation of the CLM, we said that nutrition is a question especially of several sectors. And so it's a matter of development itself.”* [UNICEF representative]

*Nutrition Enhancement Program* *(Program de Renforcement de la Nutrition, PRN)*

The PRN was implemented during 2002 to 2006, with a subsequent reform introduced in 2007 and ended in 2014. It employed a multisectoral approach and aimed to improve the nutritional status of vulnerable populations, particularly children under five years of age living in poor urban and rural areas, as well as pregnant and lactating women (117). The PRN represented a key program implemented by the CLM and also focused on strengthening institutional and organizational capacity to implement and evaluate nutrition interventions. National respondents felt that this paradigmatic shift in nutritional approaches and the institutionalization of nutrition across sectors and multiple levels of governance represented a key contribution of this nutrition program.

“*The context was favorable with a paradigm and orientation shift; a multisectoral approach, a large-scale preventive approach etc., and that's how we set up the PRN.”* [CLM representative]

*"The big difference the PRN has made is that nutrition is institutionalized, first at the sector level and then through working with the community to change behaviours.”* [UNICEF representative]

*Mandatory Fortification of Salt, Flour & Oil*

In 2009, the Government of Senegal issued a national decree, following the Accra Conference, requiring that imported and domestically produced refined vegetable oils (palm, cotton, palm kernel, peanuts, sesame, sunflower, canola, corn and soybean) be enriched with vitamin A. In addition, imported and domestically produced soft wheat flour must be enriched with iron and folic acid (83,84). In 1995, a Prime Minister’s decree was established to set standards for iodized salt production and distribution. A Presidential decree initiated mandatory iodization of all edible salt in 2001. This program has been led under the CLM since 2006. The Government of Senegal adopted the universal strategy of salt iodization (135). National respondents felt that long-standing initiatives to iodize salt led by the CLM, represented an important national and private sector collaboration to improve nutrition, however the number of private salt producers continues to represent a challenge to achieving substantial progress in this area.

*"It was the commitment of the state. It is because the state is committed that it has implemented the decree; for as long as it is not mandatory, it is difficult to work with the private sector. So, there was already a lot of enthusiasm among the partners who were very much in favour of this program implementation, but I think that the decisive factor was the decree that made the fortification mandatory. And therefore, the industrialists should and could only abide by this decree because enrichment was mandatory. I think that this was the first factor of success*.” [UNICEF representative].

*“…It is mainly the fact that the production of oil and flour is basically centralized: you have deal with a few producers. While for salt iodization you are dealing with thousand and one producers. And so these people are hard to say they do not deviate from the average.”* [UNICEF representative]

*“But also, within the framework of the CLM there is also the question of salt iodization, there is also the question of small agriculture within the framework of this program, there are also questions of fortification of food of this program and institutionally.”* [UNICEF Representative]

*Food Security Programs*

Several key informants felt that the *Child Nutrition and Food Security Project (NESA, Project Nutrition Enfant et Securite Alimentaire)* introduced in 2009 represented a key contribution towards addressing extreme poverty and hunger, and meeting the MDGs in Senegal. This was achieved through the introduction of cash transfers and social safety nets for vulnerable households including women and children. Effective targeting of vulnerable regions and strong donor funding/support were identified by key informants as factors that enabled programmatic success. Another program identified by national experts included the *Integrated Education and Nutrition Program* (PISEN) – which aimed to improve the health and wellbeing of children and women through sustainable food and nutrition security, basic education and training, as well as delivery of a comprehensive package of quality health interventions. This initiative was implemented over 2012-2016 in four high-burden regions in Senegal (Sehiou, Kolda, Kedougou and Dakar) and represented a coordinated effort among multilateral donors (141).

"*It's a program [PISEN] that has done two things: first, to get the operational level to work, to involve the operational level in the planning of priority interventions. Secondly, to involve communities, administrative authorities and local communities in taking into account their own health. And it has also made it possible jointly to evaluate the contribution of the United Nations system in the intervention zones.*” [WHO representative]

*"The idea was to go beyond small curative projects...rather than reacting, we had to have a large-scale, multisectoral, preventive approach to malnutrition. So, the goal was to expand the coverage of community nutrition interventions in Senegal... but the idea was really to target the areas where the need was greatest, where there was the most prevalence of malnutrition. So, it is within this framework that this program has been implemented with several components and has had the participation of several departments in its steering committee and the implementation of activities by these departments.”* [CLM Representative]

One national respondent also highlighted agricultural transition as a potential underlying factor, including diversification of production including increased sources of animal protein.

*"Since there are more and more efforts being made to diversify agricultural production. Diversification of agricultural production in all the sectors, eh? When I speak of the agricultural sector, agriculture strictly speaking - breeding, horticulture, aquaculture, fishing etc. everything is included in it."* [Retired Professor in Pediatrics and Nutrition]

***Nutrition-Sensitive Policies & Programs***

Long-standing efforts to implement cross- and multi-sectoral collaboration are evident and nutrition has represented a national development priority across sectoral policy letters of ministries and directorates including health, agriculture, hydraulics and sanitation, and early childhood development. Improvements across other sectors including education, health and access to water have supported gains to addressing underlying determinants of chronic malnutrition among children. Several specific nutrition-sensitive policies and program efforts were discussed by national key informants.

*“We now have nutrition-specific interventions, but there are as well sectors that develop nutrition-sensitive interventions. And as these nutrition-sensitive interventions address the determinants of malnutrition, I think, we can explain this decline by the increase of the volume or mass of nutrition.”* [CLM representative]

*“The interventions to be implemented are first and foremost based on the multi-sectoral approach. Sectors need to have nutrition goals, get the right budget, and follow their nutrition goals. This means that today, the fishing sector must have nutrition objectives including the* quality *of products and their greater distribution and accessibility. So first of all, the sectors must take ownership of nutrition issues and institutionalize them at the sectoral level. I think that if we succeed in so doing, we will have achieved a great deal to the extent that it is the sectors that will be in charge of everything. Now, in the meantime, we must also proceed with the programs that have been proven, namely, nutrition programs, health programs, vaccination programs, vitamin A supplementation programs, to be able to further reduce stunting and also to ensure prevention through a good nutrition of women, a good knowledge of nutrition, a good evolution in habits, a good evolution in beliefs and an elimination of a few barriers.”* [UNICEF Representative]

***Contributions of Community-Based Actors & Donors***

National respondents recognized the role of strong community-based actors and stakeholders, as well as grassroots activities as drivers of success in nutrition efforts. Earlier successes of local level implementation of nutrition programs, as well as an increased number of nutrition-focused experts were specifically highlighted as contributing factors.

*“What made the whole process easy is that in Senegal there was already a strong experience in nutrition at the community level. Indeed, there were already NGOs with their strong experience in program implementation in the nutrition intervention areas. We have a critical mass of nutrition stakeholders, people who were trained in that field as well as people who were trained in other fields but who were interested in nutrition, and nutritionists with an experience in getting people to act”.* [CLM representative]

In addition, the importance and contribution of diverse bilateral and multilateral donors in the implementation of nutrition-related efforts was recognized by national key informants.

"*So I think it was an effort has allowed a little to improve this indicator. Both the contribution of the state which was decisive from the political point of view as well as from the financial point of view; the intervention of donors such as the World Bank, Canada, the United States but also the United Nations system which has ensured an important leading in particular. I want to mention a little UNICEF in the questions of nutrition and child health but also civil society organizations like Nutrition International, like Helen Keller International, Save The Children, Child Fund that have implemented ..., because even in some areas they are executing agencies of the CLM programs and all of these interventions projects and programs helped to move stunting.”* [UNICEF Representative]

***Challenges to Implementation***

Several challenges were discussed by national key informants that may limit effective implementation of nutrition-specific and –sensitive efforts. These included:

- Inequitable coverage and access of programs;
- Competition and dynamics between actors and institutions;
- Limited monitoring and accountability of community actors to ensure accountability and implementation of nutrition programs;
- Limited involvement of local authorities limited sustainability;
- Inadequate support and training for nutrition experts;
- Challenges with health promotion and social behaviour change communication at community level (e.g., promotion of breastfeeding);
- Lack of focus of adolescent girls within nutrition programs;
- Sustainability of development initiatives; and
- Failure by donors to align with national priorities.

***Underlying Causes***

Underlying factors discussed by national key informants included moderate increases in access to basic resources including drinking water and sanitation facilities. The role of community health providers and the increased training and availability of nutrition experts were also underlying factors that may have facilitated improved access to health services.

*Improved household environment*

Modest improvements in access to improved drinking water were observed overtime, as the proportion of the population that obtains their drinking water from an improved source increased from 73.3% in 1992/93 to 78.5% in 2017. However, access to piped water did not vary substantially overtime. The proportion of the population with improved, non-shared toilet facilities increased from 22% in 1992/93 to 55.1% in 2017. These trends towards improve access to water and sanitation were also linked to improved nutritional status by key informants. Efforts to improve WASH were largely achieved through implementation of the multisectoral PRN program, and national respondents indicated that this has supported improvements in access to handwashing and open defecation

*"So that changed, there was also a strong tendency to promote latrines in urban and rural areas. That also had an impact - I think that's the case! There were several initiatives on handwashing, on everything about water, hygiene and sanitation too. There have been many in Senegal and there is also the fact that we have improved drinking water supply."* [CLM Representative]

"…*with the efforts being made in the field of hydraulics, people have better access to drinking water and sanitation. These are what led Senegal to huge improvements in nutritional status.*" [Hellen Keller International]

*“This is where the [PRN] program has a WASH component, so WASH is hygiene, water, sanitation. it is water, sanitation...so it's water-sanitation, a water-sanitation program that allows households to have water points so that they can wash their hands at critical moments; but also so that households no longer open defecation for whom they can have access to latrines.”* [CLM Representative]

*“Efforts in sanitation, now it started a long time ago with the PRN, with the program of strengthening in nutrition and the other projects like YAJJENDE which have important parts the "set-setals", every week, the sanitation weeks, where we sweep the whole village or the neighborhood, that's effort. The fact, for example to put garbage out of the village, you also see it is aspects and even to build latrines to allow to end the defecation in the open air and it is very important these State and NGO efforts*.” [Helen Keller International Representative]

*Increased access to health services*

The availability of health providers in Senegal has increased with the number of physicians increasing from 594 to 1,066, and the number of nursing and midwifery personnel showing modest increases from 3,287 to 4,822 between 2004 and 2016 respectively. Despite this progress, as of 2016, the density per 1,000 for both physicians and nursing/midwifery personnel remains 0.068 and 0.309 per 1,000 population (158). These numbers are far below the WHO recommendation for countries to meet the minimum threshold of 1 physician per 1,000 population. Provision of health services at the community level has supported accessibility and use of promotional, preventive and curative services through matrons, community health agents (ASC, *agents de sante communautaires*) and home-base care providers (DSDOM, *dispensateurs de sante a domicile*). Community health providers including community volunteers (*relais communautaires*) and godmothers (*bajenu gox*) provide referrals, promotional and preventive health services (159). Although the estimated number of community health providers remains below the recommended numbers, national respondents indicated that these lower cadre health providers have facilitated health promotion and referrals for health services, particularly for maternal and child health.

*"There was a very good collaboration between what is done between the health system and at the community level it is what has contributed more to the success of the project with the involvement of the communities, because that in each community leaders of the persons responsible for forming support groups, they facilitated the registration of children in the program so just after the birth there is the head of district or his representative or the bajenu gox will inform the relays you have to enlist such child in your programs, the child is enlisted the mother is followed in relation to home visits on exclusive breastfeeding and at 6months she starts to participate in the communication activities at the center of screening then she has his package of 30 bags of liquid based Nutrient Supplements (LNS)."* [Representative from Micronutrient Initiative]

In addition, one national respondent felt that the introduction of advanced bachelor and masters level university programs focusing on nutrition have been introduced since 2009 and have helped to substantially increase the capacity, knowledge and availability of specialized human resources for health.

*"I come back again to the question of the critical mass of nutritionists. Such programs need, say, highly qualified human resources, there is an insufficient critical mass of high level nutritionists to manage the overall implementation of the different activities.”* [Retired Professor of Pediatrics and Nutrition]

Overall, key informants indicated an improvement in availability of basic services including health.

*"The evolution of basic social services such as education, health, also access to drinking water have been decisive elements that have helped to progress a little bit...stunting rates at the Senegal level and allowing a significant reduction in stunting in recent years."* [UNICEF Representative]

*Improved food security & Feeding Practices*

Food security represented a substantial concern in relation to stunting and nutrition overall, and one key informant indicated that improved food security was due to decreased poverty, environmental and climate shocks, seasonality, as well as increased education. No national respondents indicated changes or improvements in food security over time.

*"The problem is the ability of poor households to access the market sufficiently to ensure diversified and varied food. There is a double barrier that arises - both the economic aspect, the ability of households to have money throughout the year to access this, we know that seasonally, there is a more difficult period than another, which is between the month of June and the month of September...the food use is the result of many factors, cultural, individual and factors of influence and education."* [Professor of Pediatrics and Nutrition]

*Breastfeeding Promotion*

Rates of exclusive breastfeeding among children under five months, demonstrated increases since 1985 from 4.6% to 42.1% in 2017. Further, early initiation (within one hour of birth) increased from 22.6% in 2005 to 33.6% in 2017. Despite the promotion of breastfeeding, the provision of a prelacteal feed (often *“tokantal”* or holy water) is a commonly reported cultural practice in Senegal, with moderate increases observed over time from 47.9% in 2005 to 52.3% in 2017 of children (160). Although rates of exclusive breastfeeding continue to be low, several national key informants indicated that efforts to promote exclusive breastfeeding have led to some improvements in chronic malnutrition and stunting.

"*And breastfeeding is being promoted. Good reason that since anyway, more and more mothers are breastfeeding children. Because there is the age, until 6 years...until six months, yes. I think it's being respected. The promotion of exclusive breastfeeding is a major determinant in the fight against stunting and there has been a whole campaign for the promotion of exclusive breastfeeding that is benefit*." [Maternal and Child Health Department, Ministry of Health

***Immediate Causes***

National respondents felt that decreased fertility, increased spacing between births and the reduction of communicable infections has supported gains in child nutrition. However, further efforts are needed to address micronutrient deficiencies, such as iodine deficiency and anaemia.

*Maternal Characteristics*

The gradual decline in fertility has contributed in some ways in reducing stunting in children. The total fertility, has decreased from 6.53 births per woman in 1990 to 4.77 in 2016 (1). Increased use of modern methods of contraception by women has increased inter-reproductive intervals or spacing. National key informants felt that this allowed women to prioritize children’s health and to avoid competition for children's access to food resources.

*"I'll say more or less, they played [a role] because family planning has advanced, interventions in relation to family planning have made progress in this country. Normally these are factors that impact on children malnutrition."*  [Nutritionist, Helen Keller International]

*"Among the malnutrition prevention elements it is the spacing of births through family planning that plays an important role because it allows mothers to space births, to have more time for themselves, and more time to devote to their children's diet."* [Division of Reproductive Health, Mother and Child's Health, Ministry of Health Representative]

*Reduced burden of child diseases*

Overall, national key informants felt that infections, including diarrhea, measles, malaria, have decreased over time and that this reduction in childhood illness has benefited and supported gains in child malnutrition and growth. These trends observed by respondents are corroborated by published evidence on communicable diseases. According to data from the DHS the prevalence of children with diarrhea in the five years preceding the survey has decreased modestly from 20.4% in 1992/93 to 17.5% in 2017. Declines were also observed for malaria prevalence (according to microscopy) from 2.9% in 2010/11 to 0.4% in 2017 (160). Drastic achievements were made to eradicate measles in Senegal as the number of cases declined from 15,367 in 1992 to 11 cases in 2017 (161).

*"As far as the other indicators are concerned, we must also take into account the global environment where there has been an improvement, especially in the area of ​​health, in relation to the childhood diseases, which have had an impact in the past. Nutritional status of children including severe epidemic diseases such as measles and malaria...but have almost disappeared! There is no longer an epidemic of malaria, no more measles epidemic. So these are the factors that are there and even the...in fact apart from diarrhea I think that most of the childhood diseases that worsened the nutritional status of these children started to disappear or to have an effect less compared to what we had in the past years. The improvement of the care of these children and the prevention of the most deadly and morbid pathologies may help to improve this."* [Professor/Academic]

*Improved dietary intake*

Substantial improvements in nutrition were associated with efforts and investments made by the State, with the support of donors, including international organizations and grassroots activities. National respondents did not discuss drivers of change relating to dietary intake directly. Despite progress, continuing nutritional challenges include high rates of anaemia among women and children, as well as iodine deficiency.

*"The only concern is the lack of micronutrients, particularly anaemia...So we have fairly high levels of anaemia, and of course for children as well as women, it is almost 66% of 0 to 5 year-old children who are anaemic and that is a real problem. We also have some difficulties with iodine deficiency. Because we realized that there were only 37% of women of reproductive age in Senegal who have satisfactory iodine status.*” [CLM Monitoring Evaluation and Officer]

*Child Characteristics*

Child characteristics including birth weight and intergenerational factors were not highlighted by national key informants as contributing to stunting declines among children in Senegal.

**Regional Stakeholder Perspectives**

In-depth interviews were conducted with 19 key informants in Diourbel, Louga and Kaolack regions including community health workers, religious leaders, traditional leaders and chiefs, teachers and educators, nurses and midwives, and NGO representatives.

## **Supplementary Appendix Table10B**: Summary of regional stakeholders

| **Participant #** | **Organization** |
| --- | --- |
| Diourbel Participant 1 | Chair of Committee Health Post, Pattar |
| Diourbel Participant 2 | Community health worker, Diourbel City |
| Diourbel Participant 3 | Teacher, Kindergarten, Diourbel City |
| Diourbel Participant 4 | Imam, Diourbel City |
| Diourbel Participant 5 | Caregiver at Recovery and Nutrition Education Centre, Diourbel City |
| Diourbel Participant 6 | Chief of Pattar Village |
| Diourbel Participant 7 | Community health worker, Pattar health post |
| Louga Participant 1 | Community health worker, Niomre |
| Louga Participant 2 | Deputy Director, Kindergarten, Louga City |
| Louga Participant 3 | Imam, Louga city |
| Louga Participant 4 | Chief Project, Action Against Hunger (ACF), Non-Governmental Organization |
| Louga Participant 5 | Midwife, Niomre Health Post |
| Louga Participant 6 | Treasurer, Committee Health Post, Niomre |
| Kaolack Participant 1 | Nurse, Sibassor health post |
| Kaolack Participant 2 | Community health worker, Pattar Health Post |
| Kaolack Participant 3 | Community health worker, ASBEF NGO (Senegalese Family Planning Association) |
| Kaolack Participant 4 | Director, Kindergarten, Diourbel City |
| Kaolack Participant 5 | Community health worker, Dialene Health Post |
| Kaolack Participant 6 | Supervisor, ASBEF NGO |

***Basic/Contextual Factors***

Poverty reduction and improvements in maternal education were distal factors recognized by regional respondents that have contributed to the reduction in stunting among children.

*Poverty Reduction*

One regional respondent felt that efforts to reduce poverty are necessary, in order to adequately address food security and availability, particularly protein. However, no respondents indicated that reductions in poverty were observed and implicated in the reduction of stunting.

*“I am advocating for an improvement of the living conditions and especially the access to food because I know by experience that some people stay months without eating fish*.” [Community Health Worker, Diourbel]

*Education*

Two regional key informants identified improvement in education and knowledge among mothers as a key factor that has driven improvements in nutrition and child mortality.

*"There are many things that did not exist but exist now. There is a decline in child mortality. The education of mothers has also played a considerable role and there are now more competence.”*[Assistant Director for the House of Toddlers, Louga]

*“Most moms do not ask for advice about breastfeeding. This is due to their ignorance. On the other hand, young mothers start asking questions about exclusive breastfeeding, because they have a higher level of education.”* [WHO?]

***Nutrition-specific and –sensitive programs/policies***

Nutrition-specific and –sensitive efforts in Diourbel, Louga and Kaolack are largely implemented by State and non-State actors (e.g., Plan International, Action against Hunger, the Red Cross, USAID, Caritas, etc.). Implementation of the PRN and other donor-led efforts were identified by regional respondents as contributing to the improvements in nutrition trends and reduction of child mortality. One respondent felt that efforts since the 2000s have been effective and supported gains in child growth.

*"There is a Nutrition Enhancement Program (PRN) and Action Against Hunger that work for malnutrition at Niomeré. The results of the PRN are satisfactory but we noted a slow down last year but currently the results are satisfactory.”* [Community Health Educator (PRN) of Niomeré].

*"There are three programs intended for improving child malnutrition. There is Caritas, the Nutrition Enhancement Program and the Red Cross. There are very good results especially the Nutrition Enhancement Program.”* [Community Relay of Pattar]

*"Red Cross has a program that improves the child's nutrition and there is also the House of the Nuns."* [Imam, Louga]

*"There is a very good trend because there has been a reduction in child malnutrition. The programs of the 2000s are better. There are indicators that explain the improvement of child malnutrition.*” [Community Health Worker, Kaolack]

The CLM was also identified as an effort implemented at national level. However, one regional key informant could not evaluate the successes or contributions of this effort.

*"There is the anti-malnutrition cell that is put in place by the state. I cannot give any results in relation to this program.”* [ASBEF NGO Supervisor, Kaolack]

***Underlying causes***

Improved access to health services and hygiene practices at household level were identified by these individuals as underlying determinants of nutrition and stunting gains.

*Increased access to health services*

The increased availability of health facilities and services at community level was recognized by one regional informant.

*“There are enough health posts now.”* [Nurse, Sibassor Health Post]

*“We recommend that mothers go to the health post for antenatal consultations…Yes, those recommendations have changed over the time.”* [Community Health Worker, Sibasor]

In particular, availability and distribution of vitamin A and other micronutrient supplementation (e.g., “plumpy nut”) occurred at community health posts was also recognized by several regional respondents as contributing to improvements in child health and nutrition.

*"I know the importance of vitamin A and in that sense there are bags that are given in health posts like plumpy nuts, it's very good for the child’s growth.”* [Treasurer health committee of Niomeré, Teacher]

*"Vitamin A is available as supplementation."* [Caregiver at the recovery and nutritional education unit]

*"There is vitamin A and possibility of de-worming for children."* [Community health worker, Member of the health committee]

Continuing challenges for access to health services identified by regional respondents included long distances for women to travel to reach health posts, and some women may not receive adequate antenatal care.

*Improved household environment*

Improved hygiene practices among households, largely handwashing, were identified by several regional respondents as contributing to improvements in health and nutrition.

*"They practice some rudimentary rules. They know all the rules of hygiene but do not apply them. Some give food to their children without washing their hands with soap and water.”* [Community Health Worker, Niomre, Louga]

*"There are families who know and apply the rules of hygiene inside the house. There are others who apply little or no hygiene, but there have been changes in the meantime."* [Health Post, Dialene, Kaolack]

*"Some families respect the hygiene rules but others don’t. It's either due to a lack of information or to negligence. There are families who practice good hygiene rules but others neglect* it.” [Community Health Worker, Sibassor Health Post, Kaolack]

***Immediate Causes***

Respondents felt that limited dietary diversity due to cultural norms and practices, poverty and affordability of food continue to impede nutrition gains. Regional respondents outlined specific recommendations regarding exclusive breastfeeding for women, however cultural traditions around prelacteal feeding also need to be addressed to ensure continued progress and improve low rates of exclusive breastfeeding.

*Dietary Intake*

Health providers described recommendations provided to communities regarding exclusive breastfeeding and complimentary feeding. Overall, recommendations appear to be in line with global guidelines as they emphasized exclusive breastfeeding for the first six months, early initiation, no provision of a prelacteal feeding and the introduction of complimentary foods after six months.

*"The children can consume the three meals of the family.”* [Community health worker, Diourbel]

*"The child should not consume anything from birth to 6 months except the breast milk, that is to say, the mother is asked to practice exclusive breastfeeding. From this age, the child receives rich and varied complementary foods such as porridge, beans and cow's milk.*” [Community health worker, Sibassor Health Post, Kaolack]

Limited variety in children’s diets and a lack of protein, in particular, were highlighted by regional respondents as key challenges. In addition, several regional respondents felt that the affordability of food, high rates of poverty and/or lack of education/knowledge hampered adequate dietary intake practices.

*"Most of the time, the children eat rice in the houses, there is no variation for the food. Meat is for adults, because it's not enough for the whole family. It is the lack of means that justifies this situation for children."* [Community health worker, Member of the health committee, Diourbel]

*"Children only eat rice with fish or couscous.”* [Chief of Pattar Village]

*"Even their mothers do not eat enough meat, let alone children. As for fruits and vegetables, they receive their portions but not enough partly because most children do not like vegetables. To see a mother who buys bananas for her child is rare. It's because they do not have the means of providing for that and there is also a mentality problem. Perhaps they also believe that the child should not eat fruit.”*[Nurse, Sibassor Health Post, Kaolack]

*“Children eat only what their parents can afford. They do not eat fruit. The consumption of meat depends on the size of the household and the means of the family. They eat enough vegetables. Families do not have enough money to provide good nutrition for children.”* [Chief of Pattar Village]

*"There are cases when even mothers have difficulties to follow the recommendations. There are several causes for not following the recommendation such as poverty and the mother's level of education."* [Physician, Project Leader at Action Against Hunger (ACF) NGO]

Many regional key informants highlighted recommendations given to women regarding exclusive breastfeeding including early initiation after birth, avoidance of a prelacteal feed and exclusively breastfeeding for six months, and generally these were in line with global recommendations. However, several also outlined that failure to adhere to these recommendations regarding exclusive breastfeeding occur frequently due to pervasive sociocultural practices and behaviours.

*"We recommend that the mother give the breast to the baby right after its birth and practice exclusive breastfeeding, which is of paramount importance for the child because the colostrum contains vitamins that protect the child against a lot of infections. We also advise her not to give the child water and not to mix breast milk with other foods for up to 6 months. This protects the child against certain diseases. The child will have a normal weight and will have a rapid growth.”* [Community Health Worker]

*"...We tell them to do exclusive breastfeeding for up to 6 months. Give the breast to the newly born baby within 30 minutes. This should not exceed 30 minutes. Midwives and matrons are used to these practices."* [Community Health Educator (CHE), Niomre, Louga]

*“Mothers are advised to provide food to their children starting at 6 months. This practice often faces socio-cultural issues that result in children receiving liquid and solid food before that age.”* [ASBEF NGO Supervisor, Kaolack]

The provision of prelacteal feeding, including holy water (“tokantal”) is a custom practiced by many women and may represent a barrier to achieving high rates of exclusive breastfeeding.

*"We recommend in our customs that the child be given holy water before the breast of its mother and that practice exists till now.*" [Chairman Pattar Health Committee]

A focus on behaviour change to transform dietary and culinary behaviours represented a potential area requiring further focus in order to improve diet diversity and nutritional gains at community level. Overcoming cultural barriers that do not allow children to reach to the middle of the communal bowl where vegetables, meat and fish are found is needed as this is considered rude/impolite.

*"In terms of food it is not everyone who comes from a rich family. The parents cannot satisfy the food needs of their children because of a lack of means."* [Imam, Louga]

*"Parents often forbid their children to eat fish."* [Nurse, Head of Sibassor Health Post]

**Mothers in Communities Perspectives**

A comparison of older and young mothers across three regions was conducted to understand the nutrition transition in Senegal at household and community levels. Mothers with children under-5 born in 1992-1997 period were selected as this represented a period prior to substantial gains in stunting reduction. Mothers with children born in the five years preceding the study (2012-2017), contrarily had the opportunity to benefit from sustained political will, as well as several, focused and community-based nutrition-relevant initiatives in Senegal, particularly those implemented in communities. Key basic, underlying and immediate drivers of stunting reduction were analyzed and compared by community and whether women had children born between 1992-1997 or 2012-2017. During FGDs in both community settings, mothers recognized multiple driving factors of improvements to the health and nutritional status of their children, affirming the need for a multifaceted, multilevel approach to target nutrition in Senegal. **Supplementary** **Appendix Table 10C** highlights a summary comparison of communities (rural/urban), and mothers with children between 1992-1997 and 2012-2017, highlighting key topics and trends across multiple drivers of change over time.

## **Supplementary Appendix Table10C:** Summary and comparison of mothers in communities

| **Location** | **Drivers** | **Mothers of children born in 1992-1997** | **Mothers of children born in 2012-2017** |
| --- | --- | --- | --- |
| **Diourbel Region (rural)** | **Distal Causes** *(For example: political context/stability/conflict, poverty reduction, education, women’s empowerment, urbanization, labour migration/remittances)* | - Poverty represents a concern | - Reduction of poverty was observed |
|  | **Basic Factors** *(Nutrition-Specific & -Sensitive Policies & Programs)* | - Vaccination is widespread | - Vaccination is widespread |
|  | **Underlying Causes** *(e.g., improved feeding practices and food security, improved care and health services, improved household environment/WASH)* | - Access to drinking water represents a concern - Poor crop/agriculture production - Lack of sanitation | - Improved food availability - Improved food quality - Existence of a health post (advice and packet of vitamins) has supported improvements |
|  | **Immediate Causes** *(e.g., improved dietary intake (infant and young child, dietary diversity, etc.), disease, maternal characteristics (parity, interpregnancy intervals, maternal age, maternal height) and child characteristics (low birthweight)* | - Good knowledge of infant and young child feeding recommendations - 4 consultations on average - Introduction of complementary foods usually from 6 months - Weaning (or cessation of breastfeeding) between 18 and 24 months - Children consume 3 to 6 meals a day children - Vitamin A supplementation is common | - Women attend 2 to 3 prenatal visits - Weaning (or Cessation of breast feeding) between 18 and 24 months - Women prepare the child's diet separately - Children consume 3 to 4 meals a day - Vitamin supplementation and packet of powder (provided by health facilities) is common - Children drink primarily water and fresh milk |
| **Diourbel Region (urban)** | **Distal Causes** | - Poverty represents a concern - Salinization of the groundwater (impacts the cultivation of vegetables) | N/A |
|  | **Basic Factors (Policies & Programs)** | - Vaccination - Existence of a nutrition center in the neighborhood - Food based on cereals | - Vaccination is widespread |
|  | **Underlying Causes** | - Lack of sanitation - Import of food products | - Sanitation and hygiene continue to represent concerns - Improved food availability - Women attend at least 4 prenatal consultations - Separate meals are prepared for the child - Food based on cereals and vegetables in orange colors - Mineral water used as a drink |
|  | **Immediate Causes** | N/A | - Improvement of diet in the last five years observed by women in communities |
| **Kaolack (rural)** | **Distal Causes** | - Poverty represents a concern | N/A |
|  | **Basic Factors (Policies & Programs)** | - Vaccination is widespread | - Vaccination is widespread - The Family Safety Aid Program *(Programme des bourses de sécurité familiale)* has helped reduce poverty among families |
|  | **Underlying Causes** | - Improved dietary intake - The existence of fluorosis - Access to drinking water represents a concern | - Separate preparation of meals for children - Availability of food products - Quality of care received at hospital - Advice provided by the health post |
|  | **Immediate Causes** | - Women attend 3 to 4 prenatal consultations - Women practice early initiation into breastfeeding - Introduction of complementary foods from 4 months - Weaning (or cessation of breastfeeding) from 18 months - Feeding the child from the family meal 4 to 6 meals a day - Vitamin A supplementation drink: water and milk | - Women attend 3 to 5 prenatal consultations between 3 and 5 - Women practice early initiation into breastfeeding - Children consume 3 to 4 meals a day, vitamins and supplements with blue and red capsules - Children drink primarily water and fresh milk - Women expressed a good knowledge of child feeding standards |
| **Kaolack Region (urban)** | **Distal Causes** | N/A | N/A |
|  | **Basic Factors (Policies & Programs)** | N/A | - Vaccination is widespread |
|  | **Underlying Causes** | N/A | - Improved food availability - Presence of the health post has supported improvements - Sanitation represents a concern |
|  | **Immediate Causes** | N/A | - Introduction of complementary foods from 6 months - Weaning between 18 and 24 months Separate preparation for the child |
| **Louga Region (rural)** | **Distal Causes** | N/A | N/A |
|  | **Basic Factors (Policies & Programs)** | - Lack of drinking water | - Vaccination of children - Fight against malaria (advice for sleeping under mosquito nets) |
|  | **Underlying Causes** | - Talk with (or lecture by) bajenu gox | - Sanitation represents a concern - Increased availability of a health post and support by midwives - Improved education - Food availability |
|  | **Immediate Causes** | - Number of CPNs between 3 and 4 - Very few post-natal visits - Food based on cereals Vitamin supplementation of the child | - Advice during antenatal visits about the importance of a rich and varied diet - Feeding the child based on cereals and vegetables - Improved diet in the last five years |
| **Louga Region (urban)** | **Distal Causes** | N/A | - Poverty represents a concern |
|  | **Basic Factors (Policies & Programs)** | - Vaccination of children | N/A |
|  | **Underlying Causes** | - Access to drinking water - Lack of sanitation | - Lack of sanitation - Improved food availability - Availability of health worker |
|  | **Immediate Causes** | - Food based on cereals (porridge, sombi, etc.) - Vitamin supplementation via community relays - Few control on what the child eats - Decline in diet in the last five years | - Advice on breastfeeding Advice during prenatal visits - Varied diet with vegetables Recommendations for complementary feeds based on vegetables and local dishes (mbaxal packet of powder) - Availability of godmothers or bajenu gox - Improved child nutrition in the last five years |

*N/A = Topic/issue was not discussed by mothers in FGD*

***Contextual/distal factors***

Mothers in both rural and urban regions, as well as of younger and older children outlined poverty as an important contextual/distal factor. Despite some mothers claiming that poverty reduction gains were made, they concur that it continues to represent a pervasive influence on nutrition and health among all mothers participating in the FGDs.

*Poverty*

Several women across regions and groups indicated that poverty had a substantial influence on nutrition and children’s health, particularly as it relates to food availability and security. A few women noted poverty alleviation gains, however the majority highlighted that further efforts to reduce poverty are critical.

*“…There is a problem with poverty that makes people precarious and impacts the health and nutrition of children.”* [Mother FGD Louga, urban, 2012-2017]

*“Food availability and poverty alleviation have impacted children’s nutrition and health”* [Mother FGD Diourbel, urban, 1992-1997]

*“The health post and the reduction of poverty have impacted nutrition and the health of children.”* [Mother FGD Diourbel, rural, 2012-2017].

***Underlying Factors***

Some improvements in food security and feeding practices and the availability of health services at health posts were observed by women in communities. Mothers felt that these represented underlying contributors to improvements in health and nutrition. Diverse challenges relating to water, sanitation and hygiene were observed across all regions and subgroups, and may represent an area of future efforts to ensure continued progress in reducing child stunting.

*Increased food security & feeding practices*

Several mothers (e.g., women in Diourbel with children born 1992-1997) indicated that the availability of food has substantially influenced the health and nutrition of children in their communities. Variation of produce by geographic location and seasonality also reportedly impacted the availability and consumption of fruits and vegetables.

*"For me hygiene and the availability of food have really impacted nutrition and children's health"* [Mother, FGD 1992-1997 Group, Diourbel]

*“In each season there are foods that are available and must be given to the child that are available during the season.”* [Mother, FGD 1992-1997 Group, Kaolack]

Many women reported receiving advice regarding exclusive breastfeeding and nutrition from midwives, family members (e.g., mother and mother-in-law) and local community health providers, specifically the bajenu gox (godmothers).

*“I listen to the bajenu gox.”* [Mother, FGD Kaolack, urban, 2012-2017]

*"The midwife advised me to wash the breast very well before giving it to the baby and to begin with the right breast before giving the left breast and allow the baby to suck enough milk before stopping. They also advise to give the baby the breast exclusively for six months and avoid giving the baby water for the first six months and if the baby cries to first verify if it is hungry, clean and if the diaper is clean and dry.”* [Mother, FGD 2012-2017, Louga Town]

*"My mother-in-law gave me advice by telling me to give the child breast every time he cries and when he wakes up.”* [Mother, FGD 2012-2017, Louga Town]

Despite this guidance from various health providers, many women across all regions and age groups reported providing newborns with a prelacteal feeding of tokantal (holy water) before breastfeeding, in accordance with local religious and cultural practice.

*“I gave them holy water because these are writings from the Quran.”* [Mother, FGD 1992-1997, Diroubel rural]

*“I gave them holy water first, because my mother-in-law told me to wait until going back to the family home to give them holy water.”* [Mother, FGD 1992-1996, Diourbel rural]

*“I gave them the "tokantal" first because it's our tradition*.” [Mother, FGD 2012-2017, Kaolack urban]

*Improved access to health services*

The availability of local health posts was highlighted by some women as an important contribution to health and nutrition of their children. Most FGD participants had attended more than four prenatal visits and received advice from health providers regarding their health and nutrition. Key nutrition-related recommendations provided by health provided included eating iron rich foods and vitamins, eating fruits and vegetables (e.g., bananas, oranges, carrots), and to pay attention to food that is high in sodium. Further, advice on women’s overall health and wellbeing were provided including resting, avoiding hard work and lifting heavy objects, sleep under an insecticide treated mosquito net, avoiding a lot of bending, avoid sitting for long periods of time and to establish walking as a habit.

*“For me, sanitation and our health post have really impacted nutrition and the health of our children.”* [Mother, FGD 2012-2017, Niomre, Louga]

*“During my last pregnancy, I went for five times to hospital for antenatal consultations. The midwife advised me to avoid any heavy tasks, to take three pills and to eat iron-rich foods.”* [Mother, FGD 2012-2017, Niomre Village, Louga]

The majority of women participating in all FGDs were assisted during delivery by skilled health providers, primarily midwives. However, several women (e.g., in Diourbel region women with children born 2012-2017) reported receiving assistance during delivery by non-skilled health providers including matrons, traditional birth attendants or bajenu gox.

*"When my child had diarrhea I did not bring him to the hospital, I followed the advice of the bajenu gox who advised me to make a solution made of water, salt and sugar, I did it for two days and the diarrhea stopped."* [Mother Group Discussion of Mothers 2012-2017, Louga Town]

*Household environment: Water, sanitation & hygiene*

Concerns were raised by some women regarding the lack of adequate sanitation facilities and stagnant water and garbage in streets. The availability of safe drinking water varied by region, and many women in Kaolack region reported substantial challenges with access to water. In Diourbel region, the salinization of the groundwater was reported by women to be a concern and it also impacts the agriculture and crops.

*“We really have problems with drinking water supply.”* [Mother, FGD 1992-1997, Kaolack]

*"The lack of public pit in the street, all the dirty water is poured into the street and our children play in the street and these waters contain microbes that give diseases to our children."* [Mother from 2012-2017 Group, Louga Town]

*“We cannot have good health without good sanitation, also in terms of food it is difficult for us to find fruits and vegetables, garbage in the street is also a problem, it is difficult to find fruit. The salinization of water is [also] a problem in this region.”* [Mother, FGD 1992-1997 Diourbel, urban]

***Immediate Causes***

Adhering to recommendations from health providers regarding children’s diet and vaccination schedules were outlined by mothers in FGDs as potential immediate causes of improved nutrition among children.

*Dietary Intake*

Recommendations by health providers relating to children’s dietary intake focused on encouraging nutritious and non-spicy food including mashed potatoes, “mbaxal”, “lakhoubissap”, ndambé, mbaxal kethiak, potato, fish, millet porridge and boiled carrots.

“*I visited the health centre the day after the baby's baptism and the midwife recommended that I give the child mashed potatoes, fish, "mbaxal saloum" because of the vitamins contained in the peanut that is an ingredient of the dish.”*[Mother, FGD with Mothers 2012-2017, Louga Town)

*"My child I prepare him to eat alone, I give him food such as mashed potatoes, vegetables and porridge that is to say all that the child must eat except rice. Yes I am very satisfied with the quality of the food I give to the child.”* [Mother, FGD 2012-2017, Louga urban]

Women reported introducing complementary food from three to five months, and few followed health providers’ recommendations regarding exclusive breastfeeding for up to six months.

*"I started giving my baby complementary food from three months beginning with the "rouye" (porridge) and adding carrots."* [Mother, FGD 2012-2017, Louga Tow]

*Decreased infection and diseases*

Vaccination of children was widespread among FGD participants across regions and age categories, and women reported that immunization sessions represented an opportunity for sharing recommendations or guidance on nutrition for children.

*“The diet has improved because during immunization sessions, you are advised the foods that must be given to the child.”* [Mother, FGD 1992-1997, Kaolack]

**Conclusion**

This qualitative analysis highlighted diverse contextual/distal, policy/program efforts, underlying and immediate causes of stunting decline according to national and community level respondents. At the national level, sustained political will in nutrition, including increased budgetary resources and the establishment of a high-level multisector coordinating body, were identified as substantial drivers to the stunting decline. In addition, the introduction of community-based efforts (e.g., PNC and PRN), as well as multisectoral collaborations have helped to institutionalize nutrition as a priority across levels and sectors. At the regional level, respondents emphasized improvements in poverty and education, as well as increased accessibility of health services at community level and provision of recommendations relating to nutrition to women. Mothers in communities highlighted some changes in terms of poverty reduction, access to health services, and widespread vaccination of children. Women reported continuing challenges with access to safe drinking water and adequate local sanitation facilities, including disposal of waste.

# **Supplementary Appendix References**

1. World Bank. World Bank Indicators [Internet]. 2018 [cited 2018 Nov 28]. Available from: https://data.worldbank.org/indicator

2. Joint Monitoring Programme for Water, Supply, Sanitation and Hygiene: Senegal [Internet]. JMP. 2017. Available from: https://washdata.org/data/household#!/

3. UNDP. Human Development Reports: Gender Development Index (GDI) [Internet]. 2018. Available from: http://hdr.undp.org/en/indicators/137906#

4. Black RE, Allen LH, qar Bhutta ZA, Caulfi eld LE, de Onis M, Ezzati M, Mathers C, Rivera J. Maternal and Child Undernutrition 1 Maternal and child undernutrition: global and regional exposures and health consequences. Lancet [Internet]. 2008 [cited 2018 Aug 21];371:243–60. Available from: www.thelancet.com

5. McSweeney C. A Climate Trend Analysis of Senegal. Famine Early Warn Syst Network—Informing Clim Chang Adapt Ser [Internet]. 2012;1–4. Available from: http://pubs.usgs.gov/fs/2012/3123/

6. The World Bank. Local Development, Institutions and Climate Change in Senegal [Internet]. 2010. Available from: http://digitalmedia.worldbank.org/slideshows/senegal-fr/]

7. WorldBank. Agriculture, forestry, and fishing, value added (% of GDP). 2017.

8. WorldBank. Employment in agriculture (% of total employment) (modeled ILO estimate). 2017.

9. Simondon KB, Benefice E, Simondon F, Delaunay V, Chahnazarian A. SEASONAL-VARIATION IN NUTRITIONAL-STATUS OF ADULTS AND CHILDREN IN RURAL SENEGAL. Ulijaszek SJ, Strickland SS, editors. Seasonality and Human Ecology. Cambridge: Cambridge Univ Press; 1993. 166–183 p.

10. Lazzaroni S, Wagner N. Misfortunes never come singly: Structural change, multiple shocks and child malnutrition in rural Senegal. Econ Hum Biol [Internet]. Elsevier B.V.; 2016;23:246–62. Available from: http://dx.doi.org/10.1016/j.ehb.2016.10.006

11. Kampman H, Park S, Rawat R, Becquey E, Zongrone A. Stories of Change Report Draft. 2016;

12. Smith LC, Ruel MT, Ndiaye A. Why is child malnutrition lower in urban than in rural areas? Evidence from 36 developing countries. World Dev. 2005;33:1285–305.

13. Kennedy G, Nantel G, Brouwer ID, Kok FJ. Does living in an urban environment confer advantages for childhood nutritional status? Analysis of disparities in nutritional status by wealth and residence in Angola, Central African Republic and Senegal. Public Health Nutr. 2006;9:187–93.

14. Maire B, Delpeuch F, Cornu A, Tchibindat F, Simondon F, Massamba JP, Salem G, Chevassus-Agnès S. Urbanisation et transition nutritionnelle en Afrique sub-saharienne : les exemples du Congo et du Sénégal. Rev Epidemiol Sante Publique. 1992;40.

15. Gupta N, Gehri M, Stettler N. Early introduction of water and complementary feeding and nutritional status of children in northern Senegal. Public Health Nutr [Internet]. 2007;10:1299–304. Available from: http://journals.cambridge.org/action/displayJournal?jid=PHN

16. Fernald LCH, Kariger P, Hidrobo M, Gertler PJ. Socioeconomic gradients in child development in very young children: Evidence from India, Indonesia, Peru, and Senegal. Proc Natl Acad Sci. 2012;109:17273–80.

17. Buttarelli E, Chapuis-Lucciani N, Badiane NS, Gueye L. Nutritional status among young children (0-3 years) in the Pikine-Dagoudane urban area in Senegal. Bull Mem Soc Anthropol Paris [Internet]. 2013;25:83–98. Available from: https://www.scopus.com/inward/record.uri?eid=2-s2.0-84875394877&doi=10.1007%2Fs13219-012-0066-2&partnerID=40&md5=82794e53f4a8a19b893d7b88a1edc443

18. Linnemayr S, Alderman H, Ka A. Determinants of malnutrition in Senegal: individual, household, community variables, and their interaction. Econ Hum Biol [Internet]. 2008;6:252–63. Available from: http://www.sciencedirect.com/science?_ob=ArticleURL&_udi=B73DX-4SH6BBV-1&_user=6686535&_coverDate=07%2F31%2F2008&_rdoc=7&_fmt=high&_orig=browse&_srch=doc-info(%23toc%2311482%232008%23999939997%23694344%23FLA%23display%23Volume)&_cdi=11482&_sort=d&_docanch

19. Nawrotzki RJ, Schlak AM, Kugler TA. Climate, migration, and the local food security context: introducing Terra Populus. Popul Environ [Internet]. 2016;38:164–84. Available from: http://rd.springer.com/article/10.1007/s11111-016-0260-0

20. Coly AN, Milet J, Diallo A, Ndiaye T, Benefice E, Simondon F, Wade S, Simondon KB. Preschool stunting, adolescent migration, catch-up growth, and adult height in young Senegalese men and women of rural origin. J Nutr [Internet]. 2006;136:2412–20. Available from: http://www.nutrition.org/

21. UNICEF. UNICEF Annual Report 2013 – Senegal. 2013.

22. Headey D, Hoddinott J, Park S. Accounting for nutritional changes in six success stories: A regression- decomposition approach. Glob Food Sec [Internet]. 2017 [cited 2018 Jan 26];13:12–20. Available from: https://ac.els-cdn.com/S2211912416300992/1-s2.0-S2211912416300992-main.pdf?_tid=9209ebf8-02c0-11e8-b418-00000aab0f6c&acdnat=1516988862_c5e6fc22372b9d1e863bd7c2f40686bc

23. Ntab B, Simondon KB, Milet J, Cisse B, Sokhna C, Boulanger D, Simondon F. A young child feeding index is not associated with either height-for-age or height velocity in rural Senegalese children. J Nutr [Internet]. 2005;135:457–64. Available from: http://www.nutrition.org/

24. Desai S, Alva S. Maternal education and child health: Is there a strong causal relationship? Demography. 1998;35:71–81.

25. Lépine A, Strobl E. The Effect of Women’s Bargaining Power on Child Nutrition in Rural Senegal. World Dev. 2013;45:17–30.

26. Smith LC, Ramakrishnan U, Ndiaye A, Haddad L, Martorell R. The importance of women’s status for child nutrition in developing countries. Food and Nutrition Bulletin. 2003. 287–288 p.

27. Gartner A, Kameli Y, Traissac P, Dhur A, Delpeuch F, Maire B. Has the first implementation phase of the Community Nutrition Project in urban Senegal had an impact? Nutrition [Internet]. 2007;23:219–28. Available from: http://www.sciencedirect.com/science/journal/08999007

28. Alderman H, Ndiaye B, Linnemayr S, Ka A, Rokx C, Dieng K, Mulder-Sibanda M. Effectiveness of a community-based intervention to improve nutrition in young children in Senegal: a difference in difference analysis. Public Health Nutr [Internet]. 2009;12:667–73. Available from: http://journals.cambridge.org/action/displayJournal?jid=PHN

29. Remans R, Pronyk PM, Fanzo JC, Chen J, Palm CA, Nemser B, Muniz M, Radunsky A, Abay AH, Coulibaly M, et al. Multisector intervention to accelerate reductions in child stunting: an observational study from 9 sub-Saharan African countries1–4. Am J Clin Nutr. 2011;94:1632–42.

30. Taren D, Almony C, Tecle S, Navarette L, Ernst K, Menard S, Diop M, Wele A. Growth of children receiving a dehydrated potato-soy protein concentrate or corn-soy blend as part of a food aid program in northern Senegal. African J Food, Agric Nutr Dev [Internet]. 2011;11:4912–33. Available from: http://www.ajfand.net/Volume11/No4/Taren10335.pdf

31. Salif Ndiaye, Papa Demba Diouf, Mohamed Ayad. Enquête Démographique et de Santé au Sénégal (EDS-II) 1992/93. 1994 [cited 2018 Jun 14]; Available from: https://dhsprogram.com/pubs/pdf/FR55/FR55.pdf

32. DHS. Sénégal. 2017;

33. WorldBank. Immunization, measles (% of children ages 12-23 months). 2017.

34. Seck I, Faye A, Mbacké Leye MM, Bathily A, Camara MD, Ndiaye P, Dia AT. [Measles epidemic and response in the region of Dakar (Senegal) in 2009]. Sante Publique [Internet]. [cited 2019 Jan 14];24:121–32. Available from: http://www.ncbi.nlm.nih.gov/pubmed/22789117

35. Garenne M. Effect of Edmonston-Zagreb high-titre vaccine on nutritional status [4]. Lancet [Internet]. 1994;344:261–2. Available from: http://libaccess.mcmaster.ca/login?url=http://ovidsp.ovid.com/ovidweb.cgi?T=JS&CSC=Y&NEWS=N&PAGE=fulltext&D=emed6&AN=24229022

36. WTF. WFP Senegal Country Brief. 2018; Available from: https://insight.wfp.org/wfp-food-

37. Magueye Marame Ndao Ingeborg Maria Breuer. Climate risk and food security in Senegal: Analysis of climate impacts on food security and livelihoods [Internet]. Senegal; 2013. Available from: https://wca.humanitarianresponse.info/fr/system/files/documents/files/Climate risk impacts on food security and livelihoods in Senegal.pdf

38. USAID. Senegal : Nutrition Profile. 2018;10:1–6.

39. Hathie I, Seydi B, Sakho-Jimbira S. Ending Rural Hunger: The Case of Senegal. 2017.

40. Simondon KB, Benefice E. Nutritional status and irrigated rice cultivation in Northern Senegal: a five-year follow-up. Ecol Food Nutr [Internet]. 2001;40:33–52. Available from: http://ovidsp.ovid.com/ovidweb.cgi?T=JS&CSC=Y&NEWS=N&PAGE=fulltext&D=caba5&AN=20013061333

41. Benefice E, Simondon K. Agricultural development and nutrition among rural populations: a case study of the middle valley in Senegal. Ecol Food Nutr [Internet]. 1993;31:45–66. Available from: http://ovidsp.ovid.com/ovidweb.cgi?T=JS&CSC=Y&NEWS=N&PAGE=fulltext&D=caba3&AN=19941411769

42. DHS. Sénégal Enquête Démographique et de Santé Continue (EDS-Continue) 2016 [Internet]. 2016. Available from: https://dhsprogram.com/pubs/pdf/FR331/FR331.pdf

43. Simondon KB. Children’s height, health and appetite influence mothers’ weaning decisions in rural Senegal. Int J Epidemiol [Internet]. 2001;30:476–81. Available from: http://ije.oxfordjournals.org.proxy.bib.ucl.ac.be:8888/content/30/3/476.long

44. Simondon KB, Simondon F, Costes R, Delaunay V, Diallo A. Breast-feeding is associated with improved growth in length, but not weight, in rural Senegalese toddlers. Am J Clin Nutr [Internet]. 2001;73:959–67. Available from: http://ovidsp.ovid.com/ovidweb.cgi?T=JS&CSC=Y&NEWS=N&PAGE=fulltext&D=caba5&AN=20013074480

45. Simondon KB, Simondon F. Age at introduction of complementary food and physical growth from 2 to 9 months in rural Senegal. Eur J Clin Nutr. 1997;51:703–7.

46. Simondon KB, Gartner A, Berger J, Cornu A, Massamba JP, San Miguel JL, Ly C, Missottee I, Simondon F, Traissac P, et al. Effect of early, short-term supplementation on weight and linear growth of 4-7-mo-old infants in developing countries: a four-country randomized trial. Am J Clin Nutr [Internet]. 1996;64:537–45. Available from: http://ovidsp.ovid.com/ovidweb.cgi?T=JS&CSC=Y&NEWS=N&PAGE=fulltext&D=caba4&AN=19961410571

47. Simondon KB, Simondon F. Infant feeding and nutritional status: the dilemma of mothers in rural Senegal. Eur J Clin Nutr [Internet]. 1995;49:179–88. Available from: http://ovidsp.ovid.com/ovidweb.cgi?T=JS&CSC=Y&NEWS=N&PAGE=fulltext&D=caba3&AN=19951403857

48. Simondon KB, Simondon F. Mothers prolong breastfeeding of undernourished children in rural Senegal. Int J Epidemiol [Internet]. 1998;27:490–4. Available from: http://ovidsp.ovid.com/ovidweb.cgi?T=JS&CSC=Y&NEWS=N&PAGE=fulltext&D=caba4&AN=19981416806

49. Caulfield LE, Bentley ME, Ahmed S. Is prolonged breastfeeding associated with malnutrition? Evidence from nineteen demographic and health surveys. Int J Epidemiol [Internet]. 1996;25:693–703. Available from: http://ovidsp.ovid.com/ovidweb.cgi?T=JS&CSC=Y&NEWS=N&PAGE=fulltext&D=caba4&AN=19971407574

50. Agne-Djigo A, Kwadjode KM, Idohou-Dossou N, Diouf A, Guiro AT, Wade S. Energy intake from human milk covers the requirement of 6-month-old Senegalese exclusively breast-fed infants. Br J Nutr [Internet]. 2013;110:1849–55. Available from: http://journals.cambridge.org/action/displayJournal?jid=BJN

51. Bork KA, Diallo A. Boys are more stunted than girls from early infancy to 3 years of age in rural Senegal. J Nutr [Internet]. 2017;147:940–7. Available from: http://jn.nutrition.org/content/147/5/940.abstract

52. Thwing J, Eckert E, Dione DA, Tine R, Faye A, Ye Y, Ndiop M, Cisse M, Ndione JA, Diouf MB, et al. Declines in Malaria Burden and all-cause child mortality following increases in control interventions in Senegal, 2005-2010. Am J Trop Med Hyg. 2017;97:89–98.

53. Fillol F, Cournil A, Cames C, Sokhna C, Simondon KB. Active malaria morbidity management has limited impact on height status of preschool Senegalese children. J Nutr [Internet]. 2010;140:625–9. Available from: http://jn.nutrition.org/cgi/content/abstract/140/3/625

54. Ntab B, Cisse B, Boulanger D, Sokhna C, Targett G, Lines J, Alexander N, Trape JF, Simondon F, Greenwood BM, et al. Impact of intermittent preventive anti-malarial treatment on the growth and nutritional status of preschool children in rural Senegal (West Africa). Am J Trop Med Hyg [Internet]. 2007;77:411–7. Available from: http://www.ajtmh.org

55. Tine RC, Ndiaye M, Hansson HH, Ndour CT, Faye B, Alifrangis M, Sylla K, Ndiaye JL, Magnussen P, Bygbjerg IC, et al. The association between malaria parasitaemia, erythrocyte polymorphisms, malnutrition and anaemia in children less than 10 years in Senegal: a case control study. BMC Res Notes [Internet]. 2012;5:565. Available from: http://libaccess.mcmaster.ca/login?url=http://ovidsp.ovid.com/ovidweb.cgi?T=JS&CSC=Y&NEWS=N&PAGE=fulltext&D=med7&AN=23057857

56. Tine RC, Faye B, Ndour CT, Sylla K, Sow D, Ndiaye M, Ndiaye JL, Magnussen P, Alifrangis M, Bygbjerg IC, et al. Parasitic Infections among Children under Five Years in Senegal: Prevalence and Effect on Anaemia and Nutritional Status. ISRN Parasitol [Internet]. 2013;2013:272701. Available from: http://libaccess.mcmaster.ca/login?url=http://ovidsp.ovid.com/ovidweb.cgi?T=JS&CSC=Y&NEWS=N&PAGE=fulltext&D=prem&AN=27335851

57. Diouf S, Diallo A, Camara B, Diagne I, Sy Signate H, Sarr M, Fall M. Parasitoses Intestinales De L’Enfant en Zone Rurale Sénégalaise (Khombole). Dakar Med [Internet]. 2000 [cited 2019 Jan 10];47. Available from: http://www.academia.edu/32251225/Parasitoses_Intestinales_De_LEnfant_en_Zone_Rurale_Sénégalaise_Khombole_

58. Diouf S, Diallo A, Camara B, Diagne I, Tall A, Signate HS, Moreira C, Sall MG, Sarr M, Fall M. Protein-caloric malnutrition with children less than five years old in rural Senegal (Khombole). Med Afr Noire [Internet]. 2000;47:225–8. Available from: http://ovidsp.ovid.com/ovidweb.cgi?T=JS&CSC=Y&NEWS=N&PAGE=fulltext&D=caba5&AN=20001416834

59. Carlier C, Moulia-Pelat JP, Ceccon JF, Mourey MS, Ameline B, Fall M, N’Diaye M, Amedee-Manesme O. Prevalence of malnutrition and vitamin A deficiency in the Diourbel, Fatick, and Kaolack regions of Senegal: Epidemiological study. Am J Clin Nutr. 1991;53:70–3.

60. Carlier C, Etchepare M, Ceccon JF, Amedee-Manesme O. Annual assessment of the vitamin A and nutritional status of children during two cross-sectional surveys. Int J Vitam Nutr Res [Internet]. 1992;62:216–20. Available from: http://ovidsp.ovid.com/ovidweb.cgi?T=JS&CSC=Y&NEWS=N&PAGE=fulltext&D=caba3&AN=19922099261

61. Rankins J, Green NR, Tremper W, Stacewitcz-Sapuntzakis M, Bowen P, Ndiaye M. Undernutrition and vitamin A deficiency in the Department of Linguere, Louga Region of Senegal. Am J Clin Nutr. 1993;58:91–7.

62. WorldBank. Children (0-14) living with HIV. 2017.

63. Jesson J, Koumakpaï S, Diagne NR, Amorissani-Folquet M, Kouéta F, Aka A, Lawson-Evi K, Dicko F, Kouakou K, Pety T, et al. Effect of Age at Antiretroviral Therapy Initiation on Catch-up Growth Within the First 24 Months Among HIV-infected Children in the IeDEA West African Pediatric Cohort. Pediatr Infect Dis J [Internet]. NIH Public Access; 2015 [cited 2019 Jan 14];34:e159-68. Available from: http://www.ncbi.nlm.nih.gov/pubmed/25955835

64. Cames C, Pascal L, Diack A, Mbodj H, Ouattara B, Diagne NR, Diallo NF, Msellati P, Mbaye N, Sy Signate H. Risk factors for growth retardation in HIV-infected Senegalese children on antiretroviral treatment: the ANRS 12279 MAGGSEN Pediatric Cohort Study. Pediatr Infect Dis J [Internet]. 2017;36:e87–92. Available from: http://journals.lww.com/pidj/Abstract/2017/04000/Risk_Factors_for_Growth_Retardation_in.13.aspx

65. Bork K, Cames C, Barigou S, Cournil A, Diallo A. A Summary Index of Feeding Practices Is Positively Associated with Height-for-Age, but Only Marginally with Linear Growth, in Rural Senegalese Infants and Toddlers. J Nutr [Internet]. 2012;142:1116–22. Available from: http://jn.nutrition.org/cgi/doi/10.3945/jn.112.157602

66. Hetherington JB, Wiethoelter AK, Negin J, Mor SM. Livestock ownership, animal source foods and child nutritional outcomes in seven rural village clusters in Sub-Saharan Africa. Agric Food Secur [Internet]. 2017;6. Available from: http://agricultureandfoodsecurity.biomedcentral.com/articles/10.1186/s40066-016-0079-z

67. Camara B, Diouf S, Dia A, Diagne I, Ousseynou Faye E, Sall G, Ba M, Sow D, Niouky F, Coumba Faye P. Etat Nutritionnel des femmes et des enfants dans la communaute rurale de Mpal (District de St. Louis - Senegal). Dakar Med [Internet]. 1999 [cited 2019 Jan 10];46. Available from: http://www.academia.edu/3765172/ETAT_NUTRITIONNEL_DES_FEMMES_ET_DES_ENFANTS_DANS_LA_COMMUNAUTE_RURALE_DE_MPAL_DISTRICT_de_ST_LOUIS_-_SENEGAL

68. Victora C G, Huttly, S R, Fuchs, S C, Olinto, M T. The role of conceptual frameworks in epidemiological analysis: a hierarchical approach. Int J Epidemiol. 1997;26:224–7.

69. Jann B. The Blinder–Oaxaca decomposition for linear regression models. Stata J. 2008;8:453–79.

70. Headey D, Hoddinott J, Park S. Accounting for nutritional changes in six success stories: A regression- decomposition approach. Glob Food Sec. 2017;13:12–20.

71. Headey DD, Hoddinott J. Understanding the Rapid Reduction of Undernutrition in Nepal. PLoS One. 2015;10:e0145738.

72. Headey D, Hoddinott J, Park S. Drivers of nutritional change in four South Asian countries: A dynamic observational analysis. Matern Child Nutr. 2016;12:210–8.

73. Woodruff BA, Wirth JP, Bailes A, Matji J, Timmer A, Rohner F. Determinants of stunting reduction in Ethiopia 2000 – 2011. Matern Child Nutr. 2017;13.

74. Alderman H, Headey D. The timing of growth faltering has important implications for observational analyses of the underlying determinants of nutrition outcomes. PLoS One. 2018;13:e0195904.

75. Restrepo-Méndez MC, Barros AJ, Black RE, Victora CG. Time trends in socio-economic inequalities in stunting prevalence: analyses of repeated national surveys. Public Health Nutr. 2014;18:2097–104.

76. Sandelowski M. Focus on Qualitative Methods Sample Size in Qualitative. Res Nurs Heal. 1995;18:179–83.

77. Green J, Browne J. Principles of Social Research. Green, J. & Browne J, editor. Maidenhead: Open University Press; 2009.

78. ANSD & Senegal Data Portal. Census Data. 2013.

79. Black RE, Victora CG, Walker SP, Bhutta ZA, Christian P, De Onis M, Ezzati M, Grantham-Mcgregor S, Katz J, Martorell R, et al. Maternal and child undernutrition and overweight in low-income and middle-income countries. Lancet. 2013;382:427–51.

80. Republique de Senegal. Presidential Decree 1994: National Commission for the Fight Against Malnutrition. 1994.

81. GAIN. Summary Report National survey on household utilisation of iodised salt and bouillon and on iodine status of women of reproductive age in Senegal 2014.

82. Iodization BFP early brain development through salt. UNICEF & GAIN [Internet]. 2018. Available from: https://www.unicef.org/nutrition/files/brighter-future_Protecting-early-brain-development-through-salt-iodization-web-final.pdf

83. Food US, Exports A, Guide E, Lagos JE, Sylla F. Senegal Post Dakar Decree 2009-872 Fortification of Refined Vegetable Oils and Soft Wheat Flour. 2017;

84. GAIN. Oil and flour fortification contributing to micronutrient intake in Senegal. 2014.

85. Ndiaye AI. COMPRENDRE LES FACTEURS POLITIQUES ET INSTITUTIONNELS CONDUISANT AU CHANGEMENT DES POLITIQUES DE NUTRITION: Le cas de Senegal. 2010.

86. Spray AL. Evolution of Nutrition Policy in Senegal.

87. Ministère de l’Agriculture et Ministère de la Santé Publique et de l’Action Sociale. Plan National d’Action pour la Nutrition [Internet]. 1996. Available from: https://extranet.who.int/nutrition/gina/fr/node/8505

88. Wickstrom, P.J., DIagne, A., & Smith A. Etude du cas du Senegal : Des débuts prometteurs , des progres inegaux. 2006.

89. Ministère de la Santé. Plan National de développment Sanitaire (PNDS) - Phase II: 2004-2008. 2004;59.

90. Senegal R du. Lettre de Politique Generale pour le Secteur de l’Education et de la Formation. 2005.

91. Republique du Senegal. Lettre de politique de developpement de la nutrition. 2001.

92. République du Sénégal. document de stratégie de réduction de la pauvreté. 2002.

93. Witter S, Dieng T, Mbengue D, Moreira I, De Brouwere V. The national free delivery and caesarean policy in Senegal: evaluating process and outcomes. Health Policy Plan [Internet]. 2010 [cited 2018 Jun 17];25:384–92. Available from: https://academic.oup.com/heapol/article-abstract/25/5/384/609864

94. Justification CET. Plan Stratégique pour la Fortification des Aliments en micronutriments au Sénégal Annexe. 2011;2006–11.

95. République du Sénégal. Document de stratégie pour la croissance et la réduction de la pauvreté 2006-2010. 2006.

96. Gouvernement du Sénégal. Plan national stratégique pour la survie de l’enfant. 2007.

97. Ministère de la santé et de la prévention. Plan National de Developpement Sanitaire 2009-2018 Sénégal. 2009.

98. SUN. Scaling up Nutrition: Senegal [Internet]. 2017 [cited 2018 Nov 7]. Available from: https://scalingupnutrition.org/sun-countries/senegal/

99. Republic of Senegal. National Strategy for Economic and Social Development [Internet]. Washington, D.C; 2012. Available from: http://www.imf.org

100. UNSCN. Impact des Systèmes Agro-Alimentaires sur la Nutrition Sénégal. 2013.

101. Tine, Justin, Sophie Faye, Sharon Nakhimovsky and LH. Universal Health Coverage Measurement in a Lower-Middle-Income Context: A Sengalese Case Study. 2014; Available from: https://www.hfgproject.org/wp-content/uploads/2014/03/UHC-Measurement-in-Senegal-Case-Study.pdf

102. Senegal R du. Plan Senegal Emergent. 2014.

103. Republique du Senegal. Politique nationale de sante communautaire. 2014.

104. Ministère de la Santé et de l’Action sociale. PLAN STRATÉGIQUE NATIONAL DE SANTÉ COMMUNAUTAIRE. 2014.

105. Advancing Partners & Communities. Community Health Systems Catalog Country Profile: Senegal - November 2016 [Internet]. 2016. Available from: https://www.advancingpartners.org/sites/default/files/catalog/profiles/senegal_chs_catalog_profile_0_0.pdf

106. Senegal R du. Document De Politique Nationale De Developpement De La Nutritition - Draft. 2015;1–94. Available from: http://www.eoren.ch/fileadmin/sites/eoren/files/mail/documents/2013-14/Ecole-Familles.pdf

107. Abdou M, Mbengue S, Sarr M, Faye A, Badiane O, Bintou F, Camara N, Mboup S, Dieye TN. Determinants of complete immunization among senegalese children aged 12– 23 months: evidence from the demographic and health survey. BMC Public Health [Internet]. 2017 [cited 2018 Jun 17];17. Available from: https://bmcpublichealth.biomedcentral.com/track/pdf/10.1186/s12889-017-4493-3?site=bmcpublichealth.biomedcentral.com

108. GINA. Community Nutrition Program (CNP)-Growth monitoring and promotion-Infants and young children/Preschool-age children (Pre-SAC). 2014.

109. Water and Sanitation Program. Water Supply and Sanitation in Senegal: Turning Finance into Services for 2015 and Beyond [Internet]. 2011. Available from: https://www.wsp.org/sites/wsp.org/files/publications/CSO-senegal.pdf

110. USAID. Senegal: Water and Sanitation Profile [Internet]. 2010. Available from: http://www.onas.sn/

111. The World Bank. Water Sector Project [Internet]. 2019. [cited 2019 Jan 11]. Available from: http://projects.worldbank.org/P002346/water-sector-project?lang=en&tab=overview

112. The World Bank. Water Sector Project - Implementation Completion Report [Internet]. 2004. Available from: http://documents.worldbank.org/curated/en/219531468759900469/pdf/30800.pdf

113. Horton, S., Blum L.S., Diouf, M., Ndiaye, B., Ndoye, F., Niang, K., Greig A. Delivering Vitamin A Supplements to Children Aged 6–59 Months: Comparing Delivery through Campaigns and through Routine Health Services in Senegal. Curr Dev Nutr. 2018;2:1–10.

114. L’Education R du SM de. Le developpment de l’education: Rapport National du Senegal. 2004.

115. Boubacar, N. & Francois R. Country Profile commised for the EFA Global Monitoring Report 2007: Strong Founations: early childhood care and education: Senegal Country Case Study. 2007.

116. CLM. Cellule de Lutte Contre la Malnutrition [Internet]. Available from: http://www.clmsn.org/

117. IEG & World Bank. SENEGAL A Decade of World Bank Support to Senegal ’ s Nutrition Program. Washington, D.C.; 2016.

118. The World Bank. Implementation Status & Results, Senegal, Nutrition Enhancement Program II (P097181). 2011;

119. PEPAM. LONG TERM WATER PROJECT (PLT) OF SENEGAL RECEIVES WORLD BANK EXCELLENCE AWARD [Internet]. 2010 [cited 2019 Jan 14]. Available from: http://www.pepam.gouv.sn/actu.php?rubr=news&id=220&type=1&lang=fr

120. The World Bank. LONG TERM WATER SECTOR PROJECT [Internet]. 2001. Available from: http://documents.worldbank.org/curated/en/203031468763827831/pdf/multi-page.pdf

121. Jean-Luc Pigeon. Projet d’approvisionnement en eau potable a long terme/Long term water supply project: Etude d’impact [Internet]. 1999. Available from: http://documents.worldbank.org/curated/en/854441468113961158/pdf/multi-page.pdf

122. The World Bank. Senegal Long Term Water Supply Project [Internet]. 1999. Available from: http://documents.worldbank.org/curated/en/221741468777918307/pdf/multi0page.pdf

123. The World Bank. Implementation Completion and Results Report for the Long Term Water Sector Project [Internet]. 2009. Available from: http://documents.worldbank.org/curated/en/177221468103761411/pdf/ICR13520P041521C0Disclosed011131101.pdf

124. The World Bank. Implementation Completion and Results Report - Water and Sanitation Millenium Project [Internet]. 2015. Available from: http://documents.worldbank.org/curated/en/326971468185396749/pdf/ICR3447-ICR-P109986-OUO-9-Box394828B.pdf

125. Independent Evaluation Group. Implementation Completion Report: Water and Sanitation [Internet]. 2010. Available from: http://documents.worldbank.org/curated/en/547871467034515357/pdf/ICRR-Disclosable-P109986-06-27-2016-1467035413857.pdf

126. The PDNL - National Program for Local Development [Internet]. [cited 2018 Oct 23]. Available from: http://www.pndl.org/-Le-PDNL-.html

127. IEG ICR Review Independent Evaluation Group. Participatory Local Development Program [Internet]. 2015. Available from: http://documents.worldbank.org/curated/en/231001467997913612/pdf/ICRR14620-P088656-Box393183B-PUBLIC.pdf

128. Ferl K and, Twigg J. ICR Review: Nutrition Enhancement Program II [Internet]. 2015. Available from: http://documents.worldbank.org/curated/en/585111468178156945/pdf/ICRR14714-P087860-Box393183B-PUBLIC.pdf

129. Health N and PGP (GHNDR). Implementation Completion and results Report: Nutrition Enhancement Project II [Internet]. 2014. Available from: http://documents.worldbank.org/curated/en/529021468296713488/pdf/ICR24490P097180IC0disclosed01050150.pdf

130. CLM. dossier de presse -lancement officiel de nutrition et transferts sociaux ciblés sur l’enfant /NETS. 2009.

131. Damiba, Andre E. Evaluation finale indépendante du programme conjoint « nutrition, enfant et securité alimentaire » (NESA) au Sénégal. 2013.

132. Cellule de Lutte contre la Malnutrition. Child Nutrition and Food Security Project (NESA) of the Malnutrition Cell [Internet]. [cited 2018 Sep 27]. Available from: http://clmsn.org/index.php/projet-et-programmes/projet-nutrition-enfant-et-securite-alimentaire-de-la-cellule-de-lutte-contre-la-malnutrition

133. IDRC. Bajenu Gox: A Community Approach to Maternal and Child Health in Senegal (IMCHA) | CRDI - Centre de recherches pour le développement international [Internet]. 2017. [cited 2019 Jan 16]. Available from: https://www.idrc.ca/fr/node/15412

134. IDRC. Maternal and child health is a community affair in Senegal | IDRC - International Development Research Centre [Internet]. 2017 [cited 2019 Jan 16]. Available from: https://www.idrc.ca/en/stories/maternal-and-child-health-community-affair-senegal

135. Cellule de Lutte contre la Malnutrition. Salt iodization project [Internet]. [cited 2018 Sep 27]. Available from: http://clmsn.org/index.php/projet-et-programmes/projet-nutrition-enfant-et-securite-alimentaire-de-la-cellule-de-lutte-contre-la-malnutrition/9-les-programmes-et-projets/90-projet-iodation-sel

136. Cellule de Lutte contre la Malnutrition, GAIN, Micrnutrient Initiative, UNICEF, Ministère de la Santé et de l’Action Sociale. National survey on household utilisation of iodised salt and bouillon and on iodine status of women of reproductive age in Senegal 2014 [Internet]. 2016. Available from: https://www.gainhealth.org/wp-content/uploads/2018/03/National-survey-on-household-utilisation-of-iodised-salt-and-bouillon-and-on-iodine-status-of-women-od-reproductive-age-in-Senegal-August-2016.pdf

137. Cellule de Lutte contre la Malnutrition. Food Fortification Strengthening Program [Internet]. [cited 2018 Oct 23]. Available from: http://clmsn.org/index.php/projet-et-programmes/programme-de-renforcement-de-la-fortification-des-aliments

138. USAID. Senegal - Mid-term Performance Evaluation of Yaajeende Agricultural Development Project 2014.pdf. 2014.

139. Peuple U, But U, Foi U. République du Sénégal L’ECOWAP/PDDAA PROGRAMME NATIONAL D’INVESTISSEMENT AGRICOLE (PNIA) [Internet]. Available from: http://www.ipar.sn/IMG/pdf/senegal_caadp_post-compact_agricultural_sector_investment_plan.pdf

140. Nsimpasi, Luyaku Loko and Chicca LF. Republic of Senegal: Country Strategic Opportunities Programme [Internet]. 2010. Available from: https://maintenance.ifad.org/documents/38711624/40234870/senegal_cosop2010.pdf/c9dd4a89-0bfa-4b94-aea9-04a7ca3714eb

141. System G of S& UN. Programme Integre Sante Education Nutrition (PISEN). 2012;

142. Republique du Senegal. National Family Safety Scholars Program (PNBSF) | Government of Senegal [Internet]. [cited 2018 Sep 27]. Available from: https://www.sec.gouv.sn/programme-national-de-bourses-de-sécurité-familiale-pnbsf

143. Organisation des Nations Unies pour l’alimentation et l’agriculture. Évaluation qualitative et prospective du Programme national de bourses de sécurité familiale au Sénégal. 2018.

144. USAID. USAID/Senegal Health Project 2016-2021 [Internet]. Available from: http://pdf.usaid.gov/pdf_docs/pa00kd87.pdf

145. Cellule de Lutte contre la Malnutrition. Plan Stratégique Multisectoriel de la Nutrition du Sénégal , 2017-2021 Sommaire. 2017;2017–21.

146. Evans M. Senegal: Mouvement des Forces Démocratiques de la Casamance (MFDC). 2004;

147. Fall A. Understanding The Casamance Conflict : A Background. Kaiptc Monogr. 2010;1–39.

148. Oxford Poverty & Human Development Initaitive. MPI: Country Level Analysis [Internet]. 2017 [cited 2018 Nov 28]. Available from: https://ophi.org.uk/multidimensional-poverty-index/databank/country-level/

149. Alkire, S., Roche, J.M., & Vaz A. Multidimensional Poverty Dynamics: Methodology and Results for 34 countries [Internet]. Oxford; 2014. Available from: https://ophi.org.uk/multidimensional-poverty-index/mpi-resources/

150. UNDP. UNDP Data Indicators [Internet]. [cited 2018 Nov 28]. Available from: http://hdr.undp.org/en/data

151. World Bank. Annual Remittances Data April 2018 [Internet]. 2018 [cited 2018 Nov 28]. Available from: http://www.worldbank.org/en/topic/migrationremittancesdiasporaissues/brief/migration-remittances-data

152. World Bank. Global Financial Development: World Bank Indicator [Internet]. 2018 [cited 2018 Nov 29]. Available from: http://databank.worldbank.org/data/reports.aspx?source=1250&series=GFDD.OI.13

153. United Nations D-PD and U. Migration Profiles - Common Set of Indicators [Internet]. 2014 [cited 2018 Dec 12]. Available from: https://esa.un.org/miggmgprofiles/indicators/indicators.htm

154. Ratha D, Mohapatra S, Ozden C, Plaza S, Shaw W, Shimeles A. LEVERAGING MIGRATION for AFRICA Remittances, Skills, and Investments [Internet]. Washington; 2011. Available from: https://siteresources.worldbank.org/EXTDECPROSPECTS/Resources/476882-1157133580628/AfricaStudyEntireBook.pdf

155. POPULATION REFERENCE BUREAU. Remittances From Sub-Saharan African Migrants Pay for Schooling, Health Care – Population Reference Bureau [Internet]. 2012 [cited 2018 Dec 12]. Available from: https://www.prb.org/remittances-subsaharan-africa/

156. Plaza S, Navarrete M, Ratha D. Migration and Remittances Household Surveys in SubSaharan Africa: Methodological Aspects and Main Findings [Internet]. 2011. Available from: http://microdata.worldbank.org/index.php/catalog/534

157. Gartner A, Maire B, Kameli Y, Traissac P, Delpeuch F. Process evaluation of the Senegal-Community Nutrition Project: An adequacy assessment of a large scale urban project. Trop Med Int Heal. 2006;11:955–66.

158. WHO. Global Health Observatory: Health Workforce Absolute Numbers & Density per 1,000. 2018.

159. Devlin K, Farnham Egan K, Pandit-Rajani T. Community Health Systems Catalog Country Profile: Senegal. 2017;

160. DHS. DHS Stat Compiler [Internet]. [cited 2018 Dec 7]. Available from: https://www.statcompiler.com/en/

161. 2017. Global Health Observatory: Measles Reported Cases by Country.
